# Safety and immunogenicity of the Euvichol-S oral cholera vaccine for prevention of *Vibrio cholerae* O1 infection in Nepal: an observer-blind, active-controlled, randomised, non-inferiority, phase 3 trial

**DOI:** 10.1016/S2214-109X(24)00059-7

**Published:** 2024-04-11

**Authors:** Katerina Rok Song, Ram Hari Chapagain, Dipesh Tamrakar, Rajeev Shrestha, Piush Kanodia, Shipra Chaudhary, T Anh Wartel, Jae Seung Yang, Deok Ryun Kim, Jinae Lee, Eun Lyeong Park, Haeun Cho, Jiyoung Lee, Patchara Thaisrivichai, Sridhar Vemula, Bo Mi Kim, Birendra Gupta, Tarun Saluja, Ruchir Kumar Pansuriya, Ravi Ganapathy, Yeong Ok Baik, Young Jin Lee, Suhi Jeon, Youngran Park, Howard L Her, Youngshin Park, Julia A Lynch

**Affiliations:** a Clinical, Assessment, Regulatory, Evaluation Unit, International Vaccine Institute, Seoul, South Korea; b Science Unit, International Vaccine Institute, Seoul, South Korea; c Department of Biostatistics and Data Management, International Vaccine Institute, Seoul, South Korea; d Department of Program Management, International Vaccine Institute, Seoul, South Korea; e Vaccine Process Development Unit, International Vaccine Institute, Seoul, South Korea; f International Vaccine Institute, Stockholm, Sweden; g Graduate School of Public Health, Yonsei University, Seoul, South Korea; h Department of Pediatric Medicine, Kanti Children's Hospital, Kathmandu, Nepal; i Center for Clinical Trial Studies, Dhulikhel Hospital, Kathmandu University Hospital, Dhulikhel, Nepal; j Department of Pediatrics and Neonatology, Nepalgunj Medical College, Nepalgunj, Nepal; k Department of Pediatrics and Adolescent Medicine, BP Koirala Institute of Health Sciences, Dharan, Nepal; l Research and Development, Hilleman Laboratories, Singapore; m Research and Development Division, EuBiologics, Seoul, South Korea; n Business Division, EuBiologics, Seoul, South Korea; o Production Division, EuBiologics, Seoul, South Korea

## Abstract

**Background:**

In October, 2017, WHO launched a strategy to eliminate cholera by 2030. A primary challenge in meeting this goal is the limited global supply capacity of oral cholera vaccine and the worsening of cholera outbreaks since 2021. To help address the current shortage of oral cholera vaccine, a WHO prequalified oral cholera vaccine, Euvichol-Plus was reformulated by reducing the number of components and inactivation methods. We aimed to evaluate the immunogenicity and safety of Euvichol-S (EuBiologics, Seoul, South Korea) compared with an active control vaccine, Shanchol (Sanofi Healthcare India, Telangana, India) in participants of various ages in Nepal.

**Methods:**

We did an observer-blind, active-controlled, randomised, non-inferiority, phase 3 trial at four hospitals in Nepal. Eligible participants were healthy individuals aged 1–40 years without a history of cholera vaccination. Individuals with a history of hypersensitivity reactions to other preventive vaccines, severe chronic disease, previous cholera vaccination, receipt of blood or blood-derived products in the past 3 months or other vaccine within 4 weeks before enrolment, and pregnant or lactating women were excluded. Participants were randomly assigned (1:1:1:1) by block randomisation (block sizes of two, four, six, or eight) to one of four groups (groups A–D); groups C and D were stratified by age (1–5, 6–17, and 18–40 years). Participants in groups A–C were assigned to receive two 1·5 mL doses of Euvichol-S (three different lots) and participants in group D were assigned to receive the active control vaccine, Shanchol. All participants and site staff (with the exception of those who prepared and administered the study vaccines) were masked to group assignment. The primary immunogenicity endpoint was non-inferiority of immunogenicity of Euvichol-S (group C) versus Shanchol (group D) at 2 weeks after the second vaccine dose, measured by the seroconversion rate, defined as the proportion of participants who had achieved seroconversion (defined as ≥four-fold increase in *V cholerae* O1 Inaba and Ogawa titres compared with baseline). The primary immunogenicity endpoint was assessed in the per-protocol analysis set, which included all participants who received all their planned vaccine administrations, had no important protocol deviations, and who provided blood samples for all immunogenicity assessments. The primary safety endpoint was the number of solicited adverse events, unsolicited adverse events, and serious adverse events after each vaccine dose in all ages and each age stratum, assessed in all participants who received at least one dose of the Euvichol-S or Shanchol. Non-inferiority of Euvichol-S compared with Shanchol was shown if the lower limit of the 95% CI for the difference between the seroconversion rates in Euvichol-S group C versus Shanchol group D was above the predefined non-inferiority margin of –10%. The trial was registered at ClinicalTrials.gov, NCT04760236.

**Findings:**

Between Oct 6, 2021, and Jan 19, 2022, 2529 healthy participants (1261 [49·9%] males; 1268 [50·1%] females), were randomly assigned to group A (n=330; Euvichol-S lot number ES-2002), group B (n=331; Euvichol-S ES-2003), group C (n=934; Euvichol-S ES-2004]), or group D (n=934; Shanchol). Non-inferiority of Euvichol-S versus Shanchol in seroconversion rate for both serotypes at 2 weeks after the second dose was confirmed in all ages (difference in seroconversion rate for *V cholerae* O1 Inaba –0·00 [95% CI –1·86 to 1·86]; for *V cholerae* O1 Ogawa –1·62 [–4·80 to 1·56]). Treatment-emergent adverse events were reported in 244 (9·7%) of 2529 participants in the safety analysis set, with a total of 403 events; 247 events were reported among 151 (9·5%) of 1595 Euvichol-S recipients and 156 events among 93 (10·0%) of 934 Shanchol recipients. Pyrexia was the most common adverse event in both groups (57 events among 56 [3·5%] of 1595 Euvichol-S recipients and 37 events among 35 [3·7%] of 934 Shanchol recipients). No serious adverse events were deemed to be vaccine-related.

**Interpretation:**

A two-dose regimen of Euvichol-S vaccine was non-inferior to the active control vaccine, Shanchol, in terms of seroconversion rates 2 weeks after the second dose. The simplified formulation and production requirements of the Euvichol-S vaccine have the potential to increase the supply of oral cholera vaccine and reduce the gap between the current oral cholera vaccine supply and demand.

**Funding:**

The Bill & Melinda Gates Foundation.

**Translation:**

For the Nepali translation of the abstract see Supplementary Materials section.

## Introduction

Cholera is an acute diarrhoeal infection caused by ingesting food or water contaminated with *Vibrio cholerae* bacteria. Although the majority of cholera cases are not reported to the WHO due to concerns about the negative impact on national tourism and agricultural trading, studies have estimated 2·86 million cases and 95 000 deaths occur annually.^1^ Climate change together with other factors (ie, political conflict, forced migration, and economic and social disruptions induced by the COVID-19 pandemic) have further propagated cholera outbreaks in the past 3 years and as of Jan 31, 2024, a new surge in infections had been reported in at least 30 countries.^2^, ^3^, ^4^ Notably, many reporting countries were either non-endemic countries (eg, Lebanon and Syria) or had not been affected by cholera for a long period of time (eg, Haiti and Dominican Republic) and the global case fatality ratio in 2021 was 1·9%, which is higher than the accepted target rate of less than 1%, with a similar trend observed in 2022 and 2023. In January, 2023, WHO raised the global cholera crisis to a grade 3 emergency, the highest level of the grading system.^5^

**Research in context**

**Evidence before this study**

The first inactivated whole-cell, bivalent (*Vibrio cholerae* O1 and O139) oral cholera vaccine, Shanchol, which contains five components, including heat-inactivated and formalin-inactivated O1 Inaba and Ogawa serotypes, was registered and prequalified by WHO on the basis of the results of a randomised, placebo-controlled trial, in which the vaccine had a 5-year cumulative efficacy of 65% against the O1 serotype in all ages. Euvichol and Euvichol-Plus, which contain five identical antigenic components and are both inactivated using heat and formalin methods, were registered and prequalified in 2015 and 2017, respectively, based on safety and immunogenicity studies. Protection against the O139 serogroup was not established in any clinical trials because of its limited distribution and infrequency of isolation and it is no longer considered a major public health threat. We searched PubMed from database inception to Nov 2, 2023, for clinical trials evaluating alternative formulations of inactivated whole-cell oral cholera vaccines using the search terms “oral cholera vaccine” and “OCV” without language restrictions. The search yielded 28 results that were then manually screened for relevance. All trials were of bivalent, five-component vaccines developed using the same technology (Shanchol, Euvichol, Euvichol-Plus, and Cholvax), with one trial of Hillchol, a monovalent whole-cell oral cholera vaccine with a formalin-inactivated single Hikojima strain coexpressing Inaba and Ogawa O1 antigens. In the phase 2/3 trial, Hillchol was found to be non-inferior to Shanchol on the basis of vibriocidal titre seroconversion rate to O1 antigens in all ages. No studies have evaluated simplified formulations of monovalent (O1) two-component (Inaba and Ogawa) vaccines inactivated by a single method (ie, formalin).

**Added value of this study**

Euvichol-S contains two formalin-inactivated components, *V cholerae* O1 Inaba El Tor strain Phil 6973 and O1 Ogawa Classical strain Cairo 50. Compared with existing inactivated, whole-cell oral cholera vaccines such as Shanchol, Euvichol, or Euvichol-Plus, the number of vaccine components was reduced from five to two and the inactivation process was simplified from two methods to one method. This change has the potential to lower production cost and complexity and increase production capacity that could increase vaccine availability, an important achievement considering the global cholera vaccine supply shortage. Our trial is the first to demonstrate the non-inferiority of Euvichol-S to Shanchol in terms of immunogenicity and safety.

**Implications of all the available evidence**

Considering the success of oral cholera vaccine in outbreak response, in 2017 WHO launched an ambitious plan, Ending Cholera - A Global Roadmap to 2030, which proposes a 90% reduction in cholera deaths by 2030 through preventive oral cholera vaccine campaigns and improvements in Water, Sanitation, and Hygiene. As currently modelled by the Global Alliance for Vaccines and Immunisation (Gavi), the strategy could avert 66–659 000 deaths and 2–27 million cases of cholera by 2035, which would require an estimated 670 million doses of oral cholera vaccine. Current vaccine production capacity falls notably short of the current and forecasted vaccine demand. Based on the manufacturers analysis, the simplified formulation might help to increase vaccine production capacity at a lower production cost, potentially increasing global supply toward the vaccine demand estimated by Gavi.

At present, three killed whole-cell bivalent (*V cholerae* O1 and O139) oral cholera vaccines, Shanchol (Sanofi Healthcare India, Telangana, India) and Euvichol and Euvichol-Plus (EuBiologics, Seoul, South Korea) are prequalified by WHO and have been made available to prevent and control cholera infection in endemic areas through Global Alliance for Vaccines and Immunisation funding.^6^, ^7^, ^8^ These vaccines, developed at the International Vaccine Institute (Seoul, South Korea) contain two heat-inactivated components (*V cholerae* O1 Inaba classical strain Cairo 48 [300 lipopolysaccharide ELISA unit (LEU)] and O1 Ogawa classical strain Cairo 50 [300 LEU]) and three formalin-inactivated components (*V cholerae* O1 Inaba El Tor strain Phil 6973 [600 LEU], O1 Ogawa Classical strain Cairo 50 [300 LEU], and O139 4260B [600 LEU]). Inaba and Ogawa are the two main serotypes for serogroup O1 and are prevalent in cholera-endemic areas. The global supply capacity of oral cholera vaccine in 2022 was 36 million doses, which was less than the 70 million doses required per year.^2^ Due to the limitations in vaccine supply and the surge in cholera outbreaks with high case fatality rates in the middle of 2021, on Oct 19, 2022, the International Coordinating Group on Vaccine Provision announced temporary suspension of the standard two-dose cholera vaccination regimen and adoption of a single dose approach.^2^, ^9^ Although the single dose strategy will allow more people to be vaccinated in a worsening global cholera situation, protection provided by the single dose strategy is shorter in duration and is limited in children, and thus not considered a long-term solution.^9^

Euvichol-S, a killed whole-cell monovalent (O1) oral cholera vaccine, which contains two formalin-inactivated components (*V cholerae* O1 Inaba El Tor strain Phil 6973 [900 LEU] and O1 Ogawa classical strain Cairo 50 [600 LEU]), was developed to optimise the oral cholera vaccine manufacturing process by simplifying the inactivation and reducing the number of components. By reducing the number of vaccine components to two, and with a rebalanced composition to achieve the same antigenic quantity of the two O1 serotypes (900 LEU of O1 Inaba and 600 LEU of O1 Ogawa per dose; appendix 2 p 92), the manufacturer's (EuBiologics, Seoul, South Korea) preliminary analyses indicated that Euvichol-S is expected to elicit immune responses supporting the current label indication (ie, prevention of cholera caused by *V cholerae*) while decreasing the cost of production and increasing vaccine production capacity. Removal of serogroup O139 is justified by its low prevalence, which never allowed the establishment of clinical efficacy against this variant and the absence of cross-reactivity against the prevalent O1 serotype.^1^, ^10^

The aim of this study was to assess the immunogenicity and safety of Euvichol-S compared with Shanchol in participants of various ages in Nepal, a cholera endemic country at risk of outbreaks.^1^, ^11^, ^12^, ^13^

## Methods

### Study design and participants

We did an observer-blind, active-controlled, randomised, non-inferiority, phase 3 trial at four sites in Nepal: Kanti Children's Hospital (Kathmandu), Dhulikhel Hospital, Kathmandu University Hospital (Dhulikhel), BP Koirala Institute of Health Sciences (Dharan), and Nepalgunj Medical College (Nepalgunj). Eligible participants were healthy, individuals aged 1–40 years. Individuals with a history of hypersensitivity reactions to other preventive vaccines, severe chronic disease, previous cholera vaccination, receipt of blood or blood-derived products in the past 3 months or other vaccine within 4 weeks before enrolment, and pregnant or lactating women were excluded. Full details of the inclusion and exclusion criteria and study procedures are provided in the protocol (appendix 2 pp 31–38, 40–42). The trial protocol was approved by Nepal Health Research Council, the ethics committees of all four sites, and the institutional review board of the International Vaccine Institute. All participants, parents, or legal guardians provided written informed consent before participation.

### Randomisation and masking

Eligible participants were randomly assigned to one of four study groups (groups A–D): participants in group A were assigned to receive Euvichol-S (lot number ES-2002), participants in group B to receive Euvichol-S (lot number ES-2003), participants in group C to receive Euvichol-S (lot number ES-2004); and participants in group D to receive Shanchol (lot number SCN034A20). Group C and D were stratified by age group (1–5, 6–17, and 18–40 years).

The non-inferiority of immunogenicity and safety of Euvichol-S versus Shanchol was assessed by comparison of group C and D, which consisted of all three age strata. Lot-to-lot consistency comparison was made in group A, B, and C in the 18-40 years age strata only. Randomisation was 1:1 between groups C and D in all ages combined and within each age stratum (1–5, 6–17, and 18–40 years) for immune non-inferiority assessment and was 1:1:1 for adults among the group A, B, and C for the assessment of lot-to-lot consistency. Multiple block size based on allocation ratio by group was applied to prevent potential unmasking. Block sizes of 2 or 4 were used for participants aged 1–5 years and 6–17 years, and block sizes of 4 or 8 were used for participants aged 18–40 years. Randomisation was done using the Medidata Rave randomisation and trial supply management system (version 1.4; Medidata, New York, NY, USA), which was preuploaded to the system with a randomisation list containing sequential numbers unique to each participant generated by an independent statistician. All participants and site staff (with the exception of those who prepared and administered the study vaccines) were masked to group assignment.

### Procedures

Euvichol-S (EuBiologics) is a killed whole-cell monovalent (O1) oral cholera vaccine and Shanchol (Sanofi Healthcare India) is a killed whole-cell bivalent (O1 and O139) oral cholera vaccine. Both vaccines were administered in 1·5 mL doses and had a yellow to yellowish appearance. Euvichol-S is contained in plastic tubes and Shanchol in glass vials. The vaccines were aspirated into a standard 3 mL syringe and administered orally by unmasked study staff to the participants to ensure masking. After obtaining written informed consent, all baseline data were collected. The participant's name, sex, and date of birth were verified by the participant's identification card. Eligible participants were randomly assigned to receive two doses of either Euvichol-S or Shanchol vaccine with a 2 week interval between doses. All participants were observed for 30 min at the site after each dose to evaluate any immediate adverse events. Participants were provided with diary cards to record any adverse events including fever, nausea or vomiting, diarrhoea, headache, fatigue, myalgia, and anorexia or loss of appetite that occurred within 7 days of dose administration. On day 7 and day 21, 7 days after the first and second dose of vaccination, there was a home visit or phone call follow-up to ensure that all adverse events were recorded accurately. Unsolicited adverse events were recorded within 28 days after each dose, and concomitant medications and serious adverse events were documented throughout the trial period (ie, up to 24 weeks after the second dose). Physical examination and vital sign measurements were performed at screening and before and after each dose of vaccination. Blood samples for immunogenicity assessment were obtained at baseline before vaccination and 2 weeks after each dose of vaccination (ie, day 0, 14, and 28). Serum samples were stored below –20°C and shipped to the International Vaccine Institute for assessment of vibriocidal antibody responses using validated assays.

For vibriocidal antibody responses, *V cholerae* O1 Inaba (T19479) and O1 Ogawa (X25049) were cultured in a brain heart infusion broth for 1·5 to 2 h reaching a mid-log phase at 37°C with a shaking incubator. The bacteria were harvested by centrifugation and cells were resuspended in sterile normal saline. A mixture containing the cultured bacteria and guinea pig complements was prepared and the bacterial mixture was added to a 96-well microtitre plate with properly diluted test serum samples in saline. After incubation for 60 min at 37°C the bacterial culture media (brain heart infusion) was added to each well of the microtitre plate and incubated at 37°C for an additional 4 h. Bacterial growth was measured at an optical density of 600 nm with a microplate reader and vibriocidal titre was defined as the highest dilution of serum that provided complete inhibition of *V cholerae* growth.^14^ The lower limit of quantitation (LLOQ) was 2·5 and below LLOQ was defined as 1·25 (half of LLOQ) for statistical analysis.

### Outcomes

The primary immunogenicity endpoint was non-inferiority of immunogenicity of the Euvichol-S vaccine (group C) versus the Shanchol vaccine (group D) at 2 weeks after the second vaccine dose in all ages combined, measured by the seroconversion rate, defined as the proportion of participants who had achieved seroconversion (defined as a ≥four-fold increase in *V cholerae* O1 Inaba and Ogawa titres compared with baseline). The primary safety endpoint was the number of solicited adverse events, unsolicited adverse events, and serious adverse events after each dose in all ages combined, and within each age stratum. Secondary immunogenicity endpoints were: geometric mean titre (GMT) for *V cholerae* O1 Inaba and Ogawa at 2 weeks after the second vaccine dose in all ages combined and within each age stratum; seroconversion for *V cholerae* O1 Inaba and Ogawa at 2 weeks after the second dose within each age stratum; and GMT for *V cholerae* O1 Inaba and Ogawa at 2 weeks after the second dose of three lots of Euvichol-S in adults. Exploratory immunogenicity endpoints were the proportion of participants who had seroconversion for *V cholerae* O1 Inaba and Ogawa at 2 weeks after the second dose of three lots of Euvichol-S in adults, and the seroconversion rate and GMT for *V cholerae* O1 Inaba and Ogawa at 2 weeks after the first dose in all ages combined and within each age stratum.

### Statistical analysis

We calculated a sample size of 2530 participants would provide around 90% power to detect non-inferiority of seroconversion rates in Euvichol-S compared with Shanchol in all ages combined (primary endpoint) and immune equivalence of three lots of Euvichol-S (secondary endpoint). Based on clinical studies conducted in India, a seroconversion rate of 71% for Shanchol in all ages was estimated.^15^ If the lower limit of two-sided 95% CI of the difference of seroconversion rates and GMT ratio of Euvichol-S and Shanchol were greater than the predefined non-inferiority margin of –10% and 0·67, respectively, Euvichol-S would be considered non-inferior. Assuming a one-tailed α of 2·5% and a 10% dropout rate, we estimated a target of 935 participants would be required for each of group C (Euvichol-S) and group D (Shanchol). The sample size of 330 participants per lot of Euvichol-S in adults would provide more than 90% power for equivalence tests of GMT ratio of immunogenicity among three lots of Euvichol-S (group A *vs* group B, group B *vs* group C, and group C *vs* group A) with overall two-sided significance level of 0·05. The equivalence margin for lot consistency assessed by seroconversion rate was –10% to 10% and assessed by GMT ratio was 0·5–2·0 based on a previously published trial of Euvichol and other oral vaccine trials;^16^, ^17^, ^18^, ^19^ the true GMT ratio was assumed to be 1 and the coefficient of variation on titre of immunogenicity was assumed to be 2·0 on the basis of oral cholera vaccine immunogenicity data.^16^, ^17^, ^18^, ^19^ All analyses were performed using SAS (version 9.4). Statistical significance was compared using a 2-sample *t* test or a Wilcoxon rank-sum test for comparing two continuous variables (eg, age, height, weight, BMI, and vital signs), and a χ^2^ test or a Fisher's exact test for categorical variables (eg, sex, childbearing potential, marital status, and adverse events). The primary immunogenicity endpoint analysis was done by generalised linear model for binomial distribution with treatment group as covariate, adjusting for age stratification and the baseline titres. All participant data were anonymised.

The primary immunogenicity endpoint was analysed in the per-protocol analysis set, which included all participants who received two vaccine administrations, had no important protocol deviations , and who provided blood samples for all immunogenicity assessments. The primary safety endpoint was assessed in all participants who received at least one dose of the Euvichol-S or Shanchol vaccine. Lot-to-lot consistency was analysed in the full analysis set, which included all participants who received at least one dose of Euvichol-S or Shanchol and provided at least one blood sample for immunogenicity assessment (modified intention-to-treat analysis). Missing immunogenicity data were not imputed for the analysis. The trial was registered at Clinicaltrials.gov, NCT04760236.

### Role of the funding source

The study funder had no role in study design, data collection, data analysis, data interpretation, or writing of the report.

## Results

Between Oct 6, 2021, and Jan 19, 2022, 2605 were screened for eligibility, of whom 2530 participants were enrolled and 2529 were randomly assigned (figure). 330 participants (aged 18–40 years) were assigned to group A (Euvichol-S [lot number ES-2002]), 331 participants (aged 18–40 years) to group B (Euvichol-S [ES-2003]), 934 participants (245 participants aged 1–5 years, 360 aged 6–17 years, and 329 participants aged 18–40 years) to group C (Euvichol-S [ES-2004]), and 934 participants (244 participants aged 1–5 years, 360 participants aged 6–17 years, and 330 participants aged 18–40 years) to group D (Shanchol; figure).

**Figure:**
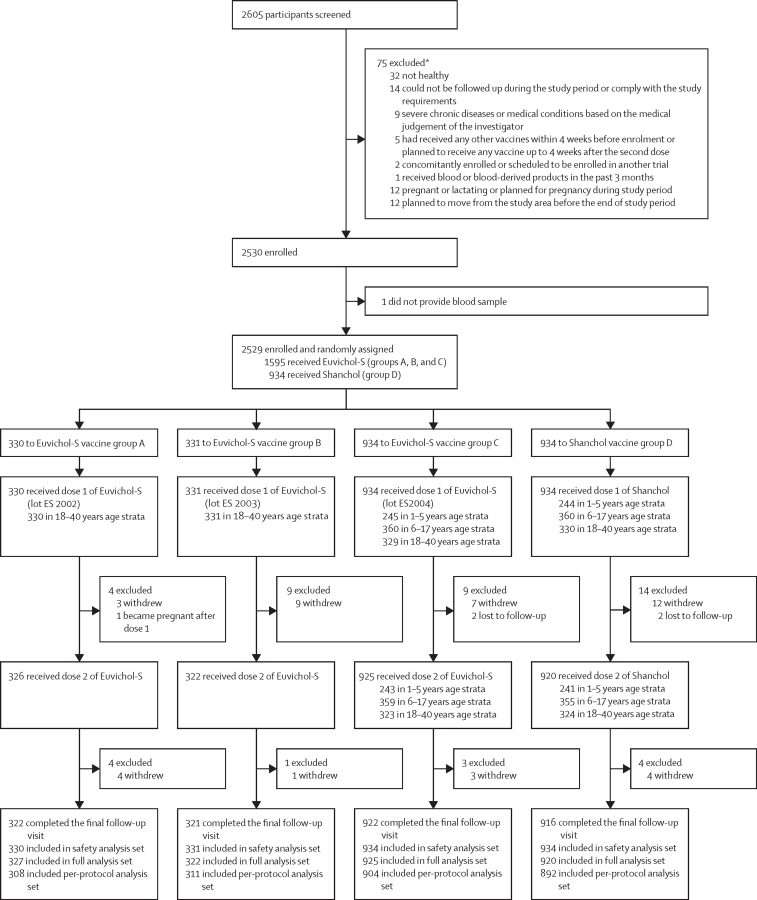
Trial profile *12 patients fulfilled two exlcusion criteria, and are included twice.

All randomly assigned participants (330 participants in group A, 331 participants in group B, 934 participants in group C, and 934 participants in group D) received at least one dose of Euvichol-S or Shanchol and were included in the safety analysis set. From the safety analysis set, 2494 participants received at least one dose of either vaccine and provided at least one blood sample for immunogenicity assessment after vaccination and thus were included in the full analysis set (327 participants in group A, 322 participants in group B, 925 participants in group C, and 920 participants in group D). From the full analysis set, 2415 participants (308 participants in group A, 311 participants in group B, 904 participants in group C, and 892 participants in group D) received both their planned doses of vaccine, provided blood samples for all immunogenicity assessments, and had no important protocol deviations and thus were included in the per-protocol set for the primary analysis of immunogenicity endpoints.

Characteristics of the full analysis set for each treatment group are presented in table 1.

**Table 1:** Demographic characteristics (full analysis set)

	**Total (n=2494)**	**Group A (n=327)**	**Group B (n=322)**	**Group C (n=925)**	**Group D (n=920)**
**Sex, n (%)**					
Male	1234 (49·5%)	147 (45·0%)	159 (49·4%)	439 (47·5%)	489 (53·2%)
Female	1260 (50·5%)	180 (55·0%)	163 (50·6%)	486 (52·5%)	431 (46·8%)
**Age, years**					
Mean (SD)	17·16 (10·82)	25·55 (6·35)	26·50 (6·00)	14·21 (10·57)	13·89 (10·16)
Median (IQR)	18·00 (7·00–25·00)	24·00 (20·00–31·00)	26·00 (21·00–31·00)	11·00 (5·00–21·00)	11·00 (5·00–20·00)
**Height, cm**					
Mean (SD)	139·81 (27·04)	158·78 (8·99)	158·87 (8·68)	133·22 (28·15)	133·02 (28·06)
Median (IQR)	150·00 (120·00–160·00)	158·00 (152·00–165·00)	158·00 (153·00–165·00)	142·00 (109·00–156·00)	142·00 (109·00–156·00)
**Weight, kg**					
Mean (SD)	41·66 (19·83)	56·67 (10·88)	57·66 (11·29)	36·51 (19·51)	35·89 (19·18)
Median (IQR)	45·00 (21·70–56·50)	55·50 (48·40–62·40)	56·10 (50·00–64·90)	35·40 (18·40–52·00)	35·00 (18·00–50·55)
**BMI, kg/m^3^**					
Mean (SD)	19·68 (4·57)	22·48 (3·96)	22·82 (4·01)	18·79 (4·42)	18·49 (4·16)
Median (IQR)	18·92 (16·02– 22·46)	21·92 (19·47–24·73)	22·14 (19·94–24·96)	17·60 (15·48–21·03)	17·53 (15·32–20·69)

For continuous variables (ie, age, height, weight, and BMI) p values were calculated using a two sample *t* test for means and Wilcoxon rank-sum test for medians. For categorical variables (ie, sex), the χ^2^ test was used.

Immune responses to *V cholerae* O1 Inaba and Ogawa following administration of vaccines for the per-protocol analysis set are provided in table 2 and table 3. Regarding the primary immunogenecity endpoint, the pre-defined non-inferiority margin (lower bound of the CI of seroconversion rate greater than –10%) was met for both serotypes. Non-inferiority in seroconversion rate for both serotypes at 2 weeks after the second dose was confirmed in all ages and each age stratum (difference in seroconversion rate for *V cholerae* O1 Inaba –0·00 [95% CI –1·86 to 1·86] for all ages, –1·41 [–5·20 to 2·38] for age 1–5 years, 0·92 [–1·74 to 3·58] for age 6–17 years, and –1·71 [–6·36 to 2·95] for age 18-40 year; difference in seroconversion rate for *V cholerae* O1 Ogawa –1·62 [–4·80 to 1·56] for all ages, –1·08 [–5·07 to 2·91] for age 1–5 years, –1·58 [–9·66 to 6·50] for age 6–17 years, and –2·43 [–5·84 to 0·98] for age 18–40 years; table 2). The exploratory endpoint of seroconversion rate at 2 weeks after the first dose showed the same pattern as that observed after the second dose and met the non-inferiority margin for both serotypes for all ages combined. Non-inferiority in seroconversion rate for both serotypes in each age stratum at 2 weeks after the first dose of Euvichol-S was confirmed, with the exception of *V cholerae* O1 Ogawa in the 1–5 age strata (difference in seroconversion rate –4·43 [95% CI –10·38 to 1·53]; table 2).

**Table 2:** Seroconversion rates of vibriocidal antibodies against *Vibrio cholerae* O1 Inaba and O1 Ogawa (per-protocol analysis set)

	**O1 Inaba**				**O1 Ogawa**			
	Group C	Group D	Adjusted difference in seroconversion rate (95% CI)*	p value†	Group C	Group D	Adjusted difference in seroconversion rate (95% CI)*	p value†
**All ages**								
n‡	904	892	..	..	904	892	..	..
2 weeks after first dose	776 (85·35%; 83·22 to 87·49)	767 (85·37%; 83·21 to 87·53)	−0·02 (−2·75 to 2·72)	0·9904	718 (79·16%; 76·70 to 81·62)	743 (82·46%; 80·21 to 84·71)	−3·30 (−6·34 to −0·27)	0·0329
2 weeks after second dose	794 (87·27%; 85·51 to 89·03)	782 (87·27%; 85·48 to 89·06)	−0·00 (−1·86 to 1·86)	0·9979	759 (81·97%; 79·67 to 84·27)	774 (83·59%; 81·09 to 86·09)	−1·62 (−4·80 to 1·56)	0·3187
**18–40 years**								
n‡	312	312	..	..	312	312	..	..
2 weeks post first dose	249 (79·27%; 75·02 to 83·52)	257 (82·38%; 78·43 to 86·34)	−3·11 (−8·62 to 2·39)	0·2677	242 (76·81%; 72·71 to 80·91)	250 (79·14%; 75·30 to 82·98)	−2·33 (−7·03 to 2·37)	0·3308
2 weeks after second dose	246 (77·03%; 73·08 to 80·99)	245 (78·74%; 74·89 to 82·58)	−1·71 (−6·36 to 2·95)	0·4730	224 (72·29%; 68·33 to 76·25)	246 (74·72%; 71·14 to 78·30)	−2·43 (−5·84 to 0·98)	0·1631
**6–17 years**								
n‡	354	342	..	..	354	342	..	..
2 weeks after first dose	320 (90·74%; 87·96 to 93·52)	309 (89·71%; 86·73 to 92·70)	1·03 (−2·72 to 4·78)	0·5919	280 (78·51%; 74·52 to 82·50)	281 (82·11%; 78·38 to 85·85)	−3·61 (−8·74 to 1·53)	0·1686
2 weeks after second dose	327 (92·03%; 89·73 to 94·34)	312 (91·12%; 88·58 to 93·66)	0·92 (−1·74 to 3·58)	0·4986	315 (86·09%; 82·59 to 89·58)	304 (87·66%; 81·20 to 94·12)	−1·58 (−9·66 to 6·50)	0·7022
**1–5 years**								
n‡	238	238	..	..	238	238	..	..
2 weeks after first dose	207 (85·88%; 81·61 to 90·16)	201 (85·34%; 80·88 to 89·80)	0·54 (−5·55 to 6·63)	0·8617	196 (83·26%; 78·60 to 87·93)	212 (87·69%; 83·73 to 91·65)	−4·43 (−10·38 to 1·53)	0·1452
2 weeks after second dose	221 (92·93%; 89·87 to 95·99)	225 (94·34%; 91·64 to 97·04)	−1·41 (−5·20 to 2·38)	0·4663	220 (92·67%; 89·54 to 95·80)	224 (93·76%; 90·89 to 96·62)	−1·08 (−5·07 to 2·91)	0·5950

Data are number of seroconverted participants (%; 95% CI), unless otherwise stated. The estimated seroconversion rates, 95% CIs, and p values were derived using the generalised linear model with identity link function for binomial distribution with treatment group as the covariate with adjustment for baseline titres. For all ages, age strata were also adjusted. The non-inferiority of Euvichol-S was confirmed if the lower limit of two tailed 95% CI of the difference in seroconversion rates between Euvichol-S (group C) and Shanchol (group D) was greater than the non-inferiority margin of −10%.

* Group C *vs* group D.

† A p value of 0·05 or less indicates a significant difference between two groups.

‡ Number of participants in the per-protocol analysis set with no missing data.

**Table 3:** Geometric mean titre of vibriocidal antibodies against *Vibrio cholerae* O1 Inaba and O1 Ogawa (per-protocol analysis set)

	**O1 Inaba**				**O1 Ogawa**			
	Group C	Group D	Adjusted difference in GMT ratio*	p value†	Group C	Group D	Adjusted difference in GMT ratio*	p value†
**All ages**								
n	904	892	..	..	904	892	..	..
Baseline	10·62 (9·11–12·38)	9·55 (8·18–11·14)	1·11 (0·90–1·38)	0·3349	16·97 (14·50–19·87)	16·28 (13·89–19·08)	1·04 (0·83–1·30)	0·7138
2 weeks after first dose	584·65 (516·12–662·29)	619·22 (546·20–702·00)	0·94 (0·79–1·13)	0·5216	551·39 (488·68–622·15)	812·66 (719·71–917·62)	0·68 (0·57–0·80)	<0·0001
2 weeks after second dose	588·35 (531·31–651·52)	583·71 (526·78–646·81)	1·01 (0·87–1·16)	0·9140	743·00 (678·06–814·14)	918·63 (837·88–1007·16)	0·81 (0·71–0·92)	0·0013
**18–40 years**								
n	312	312	..	..	312	312	..	..
Baseline	22·90 (17·29–30·33)	24·17 (18·25–32·02)	0·95 (0·64–1·41)	0·7895	53·94 (40·60–71·66)	52·50 (39·52–69·75)	1·03 (0·69–1·54)	0·8951
2 weeks after first dose	714·19 (583·84–873·63)	761·23 (622·30–931·17)	0·94 (0·71–1·25)	0·6604	991·38 (831·85–1181·51)	1221·68 (1025·09–1455·97)	0·81 (0·63–1·04)	0·0988
2 weeks after second dose	637·99 (538·13–756·38)	620·14 (523·07–735·22)	1·03 (0·81–1·31)	0·8170	973·55 (842·35–1125·19)	1035·51 (895·96–1196·80)	0·94 (0·77–1·15)	0·5542
**6–17 years**								
n	354	342			354	342	..	..
Baseline	11·78 (9·19–15·09)	8·91 (6·92–11·46)	1·32 (0·93–1·88)	0·1212	18·26 (14·04–23·76)	18·45 (14·11–24·11)	0·99 (0·68–1·44)	0·9583
2 weeks after first dose	861·71 (715·72–1037·48)	923·79 (764·80–1115·83)	0·93 (0·72–1·22)	0·6065	580·92 (476·92–707·60)	919·28 (752·12–1123·60)	0·63 (0·48–0·84)	0·0014
2 weeks after second dose	724·98 (623·65–842·77)	744·73 (638·96–868·00)	0·97 (0·79–1·21)	0·8061	841·33 (741·60–954·47)	1133·84 (997·24–1289·15)	0·74 (0·62–0·89)	0·0012
**1–5 years**								
n	238	238	..	..	238	238	..	..
Baseline	4·37 (3·39–5·62)	4·10 (3·18–5·27)	1·07 (0·75–1·52)	0·7274	5·04 (3·92–6·47)	4·40 (3·42–5·65)	1·15 (0·80–1·63)	0·4517
2 weeks after first dose	301·01 (228·67–396·22)	306·44 (232·80–403·38)	0·98 (0·67–1·45)	0·9279	254·05 (195·33–330·42)	427·07 (328·36–555·45)	0·59 (0·41–0·86)	0·0063
2 weeks after second dose	412·28 (330·88–513·71)	397·78 (319·24–495·63)	1·04 (0·76–1·41)	0·8211	452·75 (363·79–563·46)	604·33 (485·59–752·10)	0·75 (0·55–1·02)	0·0673

Data are GMT (95% CI), unless otherwise stated. GMT=geometric mean titre. N=number of participants in per-protocol analysis set with no missing data. GMT ratios and 95% CIs were calculated as the anti-logarithmic of the difference between the mean of the log-transformed data in Euvichol-S (group C) and that in Shanchol (group D) with adjustment for baseline titre (covariates) in a generalised linear model. For overall age, age strata were also adjusted. Non-inferiority of Euvichol-S was confirmed if the lower limit of two tailed 95% CI of the difference in GMT ratio between Euvichol-S (group C) and Shanchol (group D) was greater than the noninferiority margin of 0·67.

* Group C GMT *vs* group D GMT.

† A p value of 0·05 or less indicates a significant difference between groups.

GMT for each serotype was evaluated as a secondary endpoint whereby a value greater than 0·67 for the lower bound of the CI of the GMT ratio indicated non-inferiority. For *V cholerae* O1 Inaba, the GMT at 2 weeks after the second dose of Euvichol-S was non-inferior to that of Shanchol in all ages combined (GMT ratio 1·01 [95% CI 0·87–1·16]), and in each age stratum (1·04 [0·76–1·41] for age 1–5 years, 0·97 [0·79–1·21] for age 6–17 years, and 1·03 [0·81–1·31] for age 18-40 years; table 3). For *V cholerae* O1 Ogawa, the GMT at 2 weeks after the second dose of Euvichol-S was non-inferior to those of Shanchol in all ages combined (GMT ratio 0·81 [95% CI 0·71–0·92]) and in adults (0·94 [0·77–1·15]); however, the non-inferiority margin was not met in the 1–5 year age strata (0·75 [0·55–1·02]) and 6–17 year age strata (0·74 [0·62–0·89]; table 4; appendix 2 p 100). Log-transformed GMT of Euvichol-S (group C) and Shanchol (group D) in all ages at weeks 0, 2, and 4 demonstrated a similar pattern for *V cholerae* O1 Inaba and Ogawa with the GMT increasing after the first dose vaccination (week 2) and remaining stable at week 4 (appendix 2 p 101). Distribution curves of the cumulative distribution of anti-*V cholerae* O1 Ogawa vibriocidal titre at 2 weeks after the second dose in Euvichol-S (group C) and Shanchol (group D) for all ages and each age strata are in appendix 2 (p 102). The exploratory endpoint of GMT at 2 weeks after the first dose showed non-inferiority for *V cholerae* O1 Inaba for all ages combined, 6–17 years, and 18–40 years, but not for the 1–5 years age strata (0·98 [95% CI 0·67–1·45]) and not for any age strata for *V cholerae* O1 Ogawa.

**Table 4:** Solicited and unsolicited adverse events, and events that occurred in ≥1% of all participants in Euvichol-S (group C) or Shanchol (group D) after each dose (safety analysis set)

		**All ages**			**18–40 years**			**6–17 years**			**1–5 years**		
		Group C (n=934)	Group D (n=934)	p value*	Group C (n=329)	Group D (n=330)	p value*	Group C (n=360)	Group D (n=360)	p value*	Group C (n=245)	Group D (n=244)	p value*
**Solicited adverse events**													
Any event after first dose		37 (3·96%)	26 (3%)	0·1586	15 (5%)	12 (4%)	0·5501	9 (3%)	3 (1%)	0·0807	13 (5%)	11 (5%)	0·6830
	Vomiting	12 (1%)	7 (1%)	0·2489	2 (1%)	0	0·2489	5 (1%)	2 (1%)	0·4508	5 (2%)	5 (2%)	>0·9999
	Pyrexia	12 (1%)	7 (1%)	0·2489	5 (2%)	3 (1%)	0·5048	2 (1%)	0	0·4993	5 (2%)	4 (2%)	>0·9999
Any event after second dose		15 (2%)	31 (3%)	0·0169	4 (1%)	5 (2%)	>0·9999	2 (1%)	8 (2%)	0·0560	9 (4%)	18 (7%)	0·0730
	Pyrexia	10 (1%)	15 (2%)	0·3141	1 (<1%)	4 (1%)	0·3731	0	2 (1%)	0·4993	9 (4%)	9 (4%)	0·9929
After event after any dose		51 (5%)	54 (6%)	0·7631	19 (6%)	17 (5%)	0·7247	10 (3%)	10 (3%)	>0·9999	22 (9%)	27 (11%)	0·4424
**Unsolicited adverse events**													
Any event after first dose		33 (4%)	26 (3%)	0·3544	4 (1%)	4 (1%)	>0·9999	9 (3%)	5 (1%)	0·2803	20 (8%)	17 (7%)	0·6170
	Nasopharyngitis	19 (2%)	15 (2%)	0·4887	0	2 (1%)	0·4992	7 (2%)	2 (1%)	0·1769	12 (5%)	11 (5%)	0·8387
Any event after second dose		46 (5%)	36 (4%)	0·2587	5 (2%)	4 (1%)	0·7520	15 (4%)	12 (3%)	0·5562	26 (11%)	20 (8%)	0·3603
	Pyrexia	18 (2%)	8 (1%)	0·0483	2 (1%)	1 (<1%)	0·6239	7 (2%)	3 (1%)	0·2027	9 (4%)	4 (2%)	0·1621
	Nasopharyngitis	10 (1%)	5 (1%)	0·1949	0	0	NA	1 (<1%)	2 (1%)	>0·9999	9 (4%)	3 (1%)	0·0807
	Cough	11 (1%)	11 (1%)	>0·9999	2 (1%)	0	0·2489	2 (1%)	3 (1%)	>0·9999	7 (3%)	8 (3%)	0·7869
After event after any dose		75 (8%)	58 (6%)	0·1261	9 (3%)	8 (2%)	0·8010	23 (7%)	16 (4%)	0·2491	43 (18%)	34 (14%)	0·2723

Data are n (%). The safety analysis set included all participants who received at least one dose of Euvichol-S or Shanchol vaccines. Adverse events were coded using the Medical Dictionary for Regulatory Activities (version 25.0). NA=not applicable.

* χ^2^ test was used to calculate p values; Fisher's test was used if more than 20% of expected cell frequency for any one comparison was less than 5 .

Immune responses to *V* cholerae O1 Inaba and Ogawa following the administration of different Euvichol-S lots in adults in the per-protocol analysis set are provided in appendix 2 (p 95). For both serotypes, GMT ratios at 2 weeks after the second dose in groups A, B, and C met the equivalence margin (0·5–2·0), indicating there were no significant differences in the GMT ratio between those participants administered different lots of Euvichol-S (GMT ratio 1·10 [95% CI 0·86–1·40] for for *V cholerae* O1 Inaba in group A *vs* group B, 1·01 [0·79–1·29] for group A *vs* group C, and 0·92 [0·72–1·17] for group B *vs* group C; GMT ratio 1·16 [0·93–1·44] for *V cholerae* O1 Ogawa for group A *vs* group B, 0·98 [0·78–1·21] for group A *vs* group C, and 0·84 [0·68–1·05] for group B *vs* group C). Differences in seroconversion rates were also identified at 2 weeks after the second dose between groups A, B, and C, and these differences met the equivalence margin of (–10% to 10%), confirming lot-to-lot consistency for Euvichol-S (appendix 2 p 95).

Treatment-emergent adverse events were observed in 244 (9·7%) of 2529 participants in the safety analysis set, with a total of 403 events; 247 events were reported among 151 (9·5%) of 1595 Euvichol-S recipients and 156 events among 93 (10·0%) of 934 Shanchol recipients. Six of 403 treatment-emergent adverse events in four participants were immediate adverse events after any vaccination; three events among Euvichol-S recipients (two of 1595 participants) and three events among Shanchol recipients (two of 934 participants). 189 treatment-emergent adverse events in 136 participants were solicited adverse events within 7 days post any vaccination; 114 events among Euvichol-S recipients (82 [5·1%] of 1595 participants) and 75 events among Shanchol recipients (54 [5·8%] of 934 participants). 213 treatment-emergent adverse events s in 149 participants were unsolicited adverse events within 28 days after any vaccination; 132 events among 91 (5·7%) of 1595 Euvichol-S recipients and 81 events among 58 (6·2%) of 934 Shanchol recipients. Pyrexia was the most common adverse event in both groups (36 events among 56 [3·5%] of 1595 Euvichol-S recipients and 37 events among 35 [3·7%] of 934 Shanchol recipients).

The treatment-emergent adverse events were predominately grade 1 (mild) in severity (299 of 403 treatment-emergent adverse events). No grade 4 (potentially life threatening) or grade 5 (death) events were reported. 28 treatment-emergent adverse event were definitely treatment-related, 59 were probably treatment-related, 102 possibly treatment-related, 130 were unlikely to be treatment-related, and 84 were not treatment related. All treatment-emergent adverse events resolved without sequelae.

There were three serious adverse events reported in three participants who required hospital admission; one premature delivery at gestational age 35 weeks (grade 2 event; after first dose), one road traffic accident (grade 3 event; after first dose), and one typhoid fever (grade 2; after second dose); no serious adverse events were considered related to trial vaccines.

Solicited adverse events, unsolicited adverse events, and serious adverse events in groups C (Euvichol-S) and D (Shanchol) are shown in table 4. Overall, no significant differences were identified between the two groups in the incidence of solicited adverse events or unsolicited adverse events after the first dose. Participants in group C had a lower incidence of solicited adverse events (p=0·0169) after the second dose than participants in group D. However, the two groups had a comparable incidence of solicited adverse events within 7 days after the second dose. The incidence of pyrexia within 28 days of the second vaccination dose was higher in participants in group C (n=18) than group D (n=8; p=0·0483). For all pyrexia events in group C (n=18) after the second dose, ten events were judged to be not related to treatment and eight events were unlikely to be related to treatment. Among pyrexia events reported in group D (n=8), four events were deemed not related to treatment and the other four events were deemed unlikely to be related to treatment. The interval between the last vaccination dose and the onset of pyrexia ranged from 9 days to 27 days. Based on the interval between the last vaccination and the onset of the symptom, and the causality, the increased event rate in group C did not seem to be clinically significant.

More than half of the solicited adverse events and unsolicited adverse events in groups C and D occurred in the 1–5 years age stratum and the incidence of treatment-emergent adverse events tended to be higher after the second vaccination dose in the two younger age strata (1–5 years and 6–17 years) than the 18–40 years age strata (table 4).

## Discussion

Although the protective effect of Euvichol-S against clinically apparent *V cholerae* was not evaluated in the study, the non-inferiority in immunogenicity outcomes compared with Shanchol infers a similar level of efficacy. In a randomised controlled trial in India, a neighbouring country of Nepal that also reported a cholera outbreak in 2022,^4^, ^20^, ^21^ Shanchol had a 5-year cumulative protection efficacy of 65%.

In this study, the non-inferiority of Euvichol-S compared with Shanchol, as measured by seroconversion rates of vibriocidal antibodies at 2 weeks after the second dose, was demonstrated for *V cholerae* O1 Inaba and O1 Ogawa in all ages combined and within each age stratum. Although non-inferiority in terms of immunogenecity by GMT for *V cholerae* O1 Inaba and Ogawa at 2 weeks after the second dose in all ages combined was shown, the non-inferiority margin was not met for Ogawa in the two age strata of participants younger than 18 years (GMT ratio 0·75 [95% CI 0·55–1·02] for 1–5 years; 0·74 [0·62–0·89] for 6–17 years). One of the differences between the two vaccines is that Shanchol contains both heat-inactivated and formalin-inactivated cells and Euvichol-S contains only formalin-inactivated whole cells. Vibriocidal antibody response**s** are largely driven by IgM targeting the immunodominant O-specific polysaccharide or lipopolysaccharide antigens of *V cholerae*.^22^, ^23^, ^24^ Formalin inactivation occurs by protein cross linking and is not known to affect polysaccharides. Therefore, no difference in the immunodominant antigen is expected to result from the use of only formalin versus a mixture of heat and formalin for inactivation. Furthermore, immune responses to the Inaba serotype should have been similarly affected as the difference in method of inactivation exists for Inaba yet non-inferiority of the seroconversion rate and GMT was met for all age strata. Therefore, the reason for not acheiving non-inferiority for the Ogawa GMT ratio among the 1–5 and 6–17 year age groups remains unclear considering that the Ogawa antigen content is identical for Euvichol-S and Shanchol. The differences in GMT might reflect unexpected variation in the human response not balanced by randomisation.

The two serotypes, O1 Inaba and O1 Ogawa, are differentiated based on the presence or absence of a single methyl group on the terminal portion of the O-specific polysaccharide or lipopolysaccharide, whereby the Ogawa serotype contains a methyl group lacking in the Inaba serotype. These serotypes have been shown to be cross-reactive in vivo and in vitro. On recovery from experimental infection with wild type *V cholerae* O1, either Inaba or Ogawa, there is strong protection on subsequent re-exposure to either wild type serotype.^25^ Cross serotype protection is most highly correlated with O1 Inaba antibody responses, and is the basis for monostrain cholera vaccines that provide protection against either O1 Inaba or Ogawa exposure.^26^ Vaxchora is an oral live attenuated *V cholerae* O1 classical monostrain Inaba-based vaccine that has been approved by the US Food and Drug Administration for protection against cholera of either O1 serotype as demonstrated through human challenge studies.^27^ In early field studies done in the Philippines where Ogawa was the predominant circulating serotype**,** cross serotype protection was observed after administration of killed whole-cell monovalent vaccines (both Ogawa and Inaba).^28^ However, in a study in Bangladesh, injected killed whole-cell Inaba protected against both Inaba and Ogawa in all age cohorts, while parenteral vaccination with killed whole-cell Ogawa only had diminished protection against circulating Inaba.^29^

Infection with *V cholerae* results in the development of vibriocidal antibodies and protection from infection for a period of time.^30^ Although, the protective titre threshold has not been precisely defined, a study evaluating the correlation between vibriocidal titres and protection from infection among household contacts of cholera cases including children found that titres greater than 160 were highly correlated with protection.^31^ Therefore, the proportion of participants with a vibriocidal titre greater than 160 might be another meaningful way to assess the likely clinical effect based on serological response. Our study results showed that the proportion of participants in groups C and D that achieved vibriocidal titres greater than 160 for Ogawa and Inaba was similarly high. When taking into account the titres achieved and the cross protection that Inaba response provides to Ogawa exposure, minor differences in GMT for Ogawa are unlikely to result in a measurable clinical difference. Future field effectiveness studies of the vaccine should explore whether there is a meaningful decrement in protection against Ogawa in any age group.

Euvichol-S does not contain the *V cholerae* O139 serogroup due to its low prevalence, therefore the vaccine is not expected to elicit protection against O139.

Safety data show that Euvichol-S was safe and well-tolerated, with no unexpected safety signals observed. Moreover, the safety profile of Euvichol-S was comparable with that established for Shanchol, for which millions of doses have been administered since its approval in 2009.^15^

This trial has several limitations. First, the study aim was to evaluate immunogenicity and did not include clinical efficacy against cholera. During the clinical trial, no cholera cases were reported or any diarrhoeal illness that was suspected to be cholera although the study was conducted between October, 2021, and August, 2022, inclusive of the monsoon cholera season in Nepal. Second, this study did not include individuals older than 40 years or specific subgroups, such as pregnant and lactating women, or people with HIV, which represent important subpopulations who require inclusion in future studies. Third, the participants were followed up for 6 months after the final vaccine dose, therefore, long-term data are not available. Fourth, the trial was conducted in only one country. Nepal was selected because cholera is endemic in the country and flooding and landslides are a risk during the rainy season every year, which often lead to the breakdown of the water and sanitation infrastructure, increasing the possibility of cholera outbreaks. Despite that, the study was conducted in one country, the study included four sites across the country to reflect results from different areas.

The addition of Euvichol-S to the available vaccine supply could be an important step towards increasing availability of oral cholera vaccine and achieving the goals of Ending Cholera: A Roadmap to 2030, proposed by the Global Task Force on Cholera Control of WHO and endorsed by the World Health Assembly, which requires an estimated 670 million doses of oral cholera vaccine by 2030 for pre-emptive use in high-risk areas to optimise disease prevention.^32^, ^33^

## Data sharing

Individual participant data that underlie the results reported in this article, after de-identification (text, tables, figures, and appendices), including data dictionaries and analytic code, will be made publicly available on publication from https://data.mendeley.com.

## Declaration of interests

The authors include staff at the International Vaccine Institute who developed the oral cholera vaccine technology and Eubiologics, the manufacturers of Euvichol, Euvichol-Plus, and Euvichol-S. The five-component whole-cell oral cholera vaccines Shanchol, Euvichol, and EuvicholPlus and two-component Euvichol-S were based on technology developed at the International Vaccine Institute. EuBiologics is the commercial manufacturer of Euvichol, EuvicholPlus, and Euvichol-S.

## Supplementary Materials

Nepali translation of the abstract

Supplementary appendix 2

Equitable Partnership Declaration
