# Supplementary material for: Safety and immunogenicity of the Euvichol-S oral cholera vaccine for prevention of Vibrio cholerae O1 infection in Nepal: an observer-blind, active-controlled, randomised, non-inferiority, phase 3 trial
Source: Lancet Glob Health. 2024 Apr 11;12(5):e826–37. doi: 10.1016/S2214-109X(24)00059-7 (PMC11027156; doi:10.1016/S2214-109X(24)00059-7)
Supplement: Nepali translation of the abstract [file mmc1.pdf]

THE LANCET
Global Health

Supplementary appendix 1

This translation in Nepali was submitted by the authors and we reproduce it as supplied. It has not been peer reviewed. *The Lancet*'s editorial processes have only been applied to the original in English, which should serve as reference for this manuscript.

यो अनुवाद नेपाली भाषामा लेखकहरूद्वारा बुझाइएको हो र हामीले हुबहु उपलब्ध गराएका छौं । यो अनुवादलाई सहकर्मी समीक्षा (peer reviewed) गरिएको छैन। ल्यान्सेट (Lancet) को सम्पादकीय प्रक्रियाहरू मूल अंग्रेजी भाषाको अनुवादमा मात्र लागू गरिएको छ, जसले यस पाण्डुलिपि (manuscript) को लागि सन्दर्भको रूपमा काम गर्छ।

Supplement to: Song KR, Chapagain RH, Tamrakar D, et al. Safety and immunogenicity of the Euvichol-S oral cholera vaccine for prevention of *Vibrio cholerae* O1 infection in Nepal: an observer-blind, active-controlled, randomised, non-inferiority, phase 3 trial. *Lancet Glob Health* 2024; **12:** e826–37.

सारांश

पृष्ठभूमि

विश्व स्वास्थ्य संगठनले, सन् २०१७ अक्टोबरमा, सन् २०३० भित्र हैजा उन्मुलन गर्ने रणनीति लिएको थियो/ सो रणनीतिको मुख्य चुनैती भनेको मुखबाट खुवाइने हैजा बिरुद्धको खोप को बिश्व स्तरमा आपूर्तिको कमि र सन् २०२१ देखि हैजाको प्रकोप फैलनु हो/ विश्व स्वास्थ्य संगठनबाट पूर्व स्वीकृत प्राप्त मुखबाट खुवाइने हैजा विरूद्ध को खोप Euvichol-Plus, लाई केहि तत्व हटाएर तथा केहि उत्पादन विधिलाई निस्तेज गरेर सुधारिएको खोप बाट हाल ब्याप्त हैजा खोपको कमीलाई सम्बोधन गर्न यो अनुसन्धानको महत्व रहेको छ / यस अध्यनको उद्देश्य भनेको eubiologics दक्षिण कोरिया द्वारा निर्मित सरलीकृत युभिकोल, युभिकोल-यस, (simplified Euvichol, Euvichol-S) को प्रतिरक्षात्मक प्रतिक्रिया (immunogenicity) तथा सुरक्षा (safety) लाई सनोफी हेल्थकेयर (sanofi health care) इन्डिया द्वारा निर्मित सानकोल (shanchol) संग तुलनात्मक रुपमा हेर्नु हो /

बिधि:

नेपालको ४ ओटा अस्पतालहरुमा , अबलोकनकर्ता अनभिज्ञ हुने र सहभागीहरु नियमीत नहुने(randomized) गरि सक्रिय खोप संग तुलना गर्ने तेश्रो चरणको परिक्षण गरियो जसमा स्वतन्त्र रुपमा १ देखि ४० बर्ष सम्मका, पछिल्लो तीन महिना सम्म रगत तथा रगत जन्य पदार्थ नलिएका र गर्भवती तथा स्तनपान गराउदै गरेका तथा दिर्घ रोगीहरुलाई सहभागी नगराई पहिले हैजाको खोप नपाएका र बिगत माअन्य कुनै खोप लगाउदा अतिसंबेन्दसिलाता(Hypersiesitvity) नभयका लाई अनियमीतता (randomized) गरेर Euvichol-S वा सक्रिय कन्ट्रोल Shanchol को दुइ खुराक खोप दिईएको थियो/ सहभागीहरुलाई १:१:१:१ ब्लक गरि चार समूह(समूह A-D)मा अनियमिता (randomized) गरिएको थियो जसमा ब्लक अनियमितता (ब्लक आकार दुई, चार, छ वा आठ) थियो / समूह C र D मा उमेरहद (१-५, ६-१७ र १८-४० वर्ष)को आधारमा बर्गिकरण गरिएको थियो/ समूह A-C का सहभागी हरुले १.५ मि लि Euvichol-S को दुई खुराक (तीन भिन्न लटका) पाएका थिए भने समूह D ले सक्रिय Shanchol खोप पाएका थिए/ सबै सहभागीहरु र खोप तयारी गरि लगाउने कर्मचारी बाहेकका अनुसन्धान सदस्यहरुलाई यो बर्गिकरण बारे अनविज्ञ राखिएको थियो /

यसको प्राथमिक उदेश्य भनेको यस अध्ययनमा सहभागीहरुको प्रोटोकल बिश्लेषण सेट अनुसार सबै उमेर समुहका सहभागीले हैजा खोपको दोश्रो खुराक खाएको दुई हप्ता पश्चात anti-Vibrio cholerae O1 Inaba र Ogawa vibriocidal titer   Euvichol-S को सेरो परिबर्तन दर Shanchol को अनुपातमा कमी नरहेको भनेर देखाउनु रहेको र सम्पूर्ण उमेर समुहमा सुरक्षित छ भनेर देखाउनु रहेको थियो/ योजना अनुरुप को सबै खोप पाएका, प्रोटोकल बिचलन (Protocol deviation) नभएका तथा प्रतिरक्षात्मक प्रतिक्रिया (immunogenicity)को लागि दुई पटक रगत दिएकाहरुलाई पूर्व प्रोटोकल बिस्लेषण समूहमा

राखी प्रतिरक्षात्मक प्रतिक्रिया (immunogenicity)को प्राथमिक अन्तिम बिन्दुको रुपमा अध्ययन गरिएको थियो/ प्राथमिक सुरक्षा अन्तिम बिन्दु (primary saftey end point) Euvichol-S वा Shanchol मध्ये कुनै एकको न्युनतम एक खुराक खोप खाएका, सबै ब्लक समुहका साथै सबै उमेर समुहका सहभागीलाई प्रक्षेपीत (soliciated) तथा प्रक्षेपीत नगरिएको (unsolicated) र अवान्छित घटनालाई लिईएको थियो/

Euvichol-S को गैर हिनता(non-inferiority) पुष्टि यदि Euvichol-S र Shanchol परिबर्तनदरको भिन्नता सुनिश्चितता अन्तराल (confidence interval) को तल्लो सिमा -१० प्रतिशत भन्दा माथि रहेमा गर्न सकिन्छ / यो अनुसन्धान www.clinicaltrials.gov मा क्रम संख्या NCT04760236 मा दर्ता रहेको छ /

नतिजाहरु :

सन् २०२१ अक्टोबर ६ देखि सन् २०२२ जनावरी १९ सम्ममा २५२९ स्वस्थ्य नेपाली सहभागीहरुमध्ये १२६१ (४९.९ %) पुरुष र १२६८ (५०.१ %) महिला थिए/ समूह ए मा ३३०, बि मा ३३१ र समूह सी मा ९३४ ले भिन्न फरक तीनलटका Euvichol-S खोप र समूह डी मा ९३४ सहभागीहरुले Shanchol खोप पाएका थिए/ दोश्रो खोप पायको दोश्रो हप्ता पश्चात, समस्टिगत रुपमा सम्पूर्ण उमेर समुहमा V. cholerae O1 Inaba र Ogawa बिरुद्द Euvichol र Shanchol बिचको सेरो परिबर्तन दर को फरक –0.00 [95% CI: –1.86, 1.86] र –1.62 [95% CI: –4.80, 1.56] थियो /

दुई हजार पाँच सय उनान्तीस मध्ये २४४ (९.७%) सहभागीमा उपचार चाहिने प्रतिकुल घटनाहरु भएका थिए / चार सय तीन प्रकारका विभिन्न समस्या देखिएका थिए जसमध्ये २४७ वटा (१५१/१५९५,९.५%) Euvichol-S खाएका र १५६ (९३/९३४, १०%) Shanchol खाएका हरुमा थिए/ दुवै प्रकारका खोप खाएका सहभागीहरुमा ज्वरो घटना {५७, ५६(३.५%) Euvichol-S खाएका १५९५ मध्ये), ३७,३५(३.७% Shanchol खाएका ९३४ मध्ये)} प्रमुख खोप पश्चात अवान्छित घटना थियो/ खोप कै कारण कुनै जीबनलाई हानी पुर्याउने घटनाहरु भएका थिएनन् /

ब्याख्या :

दुई खुराक खोप खाएको दुई हप्ता पश्चात Euvichol-s खोपले सक्रिय Shanchol खोप पाएकाहरु भन्दा कम anti-V. cholerae O1 Inaba र Ogawa vibriocidal टाईटर परिबर्तन नभएको पुष्टि हुन्छ/ सरलीकृत सुत्रिकरण तथा सरलीकृत उत्पादन अबश्यकताले ओसिभी खोप को आपूर्ति वृद्धि हुने सम्भावना भई वर्तमान खोप आपूर्ति र माग बिचको खाडल कम गर्न सक्ने देखिन्छ/

अनुवादक (Translator): डा.राम हरि चापागाईं
