# Supplementary material for: Safety and immunogenicity of the Euvichol-S oral cholera vaccine for prevention of Vibrio cholerae O1 infection in Nepal: an observer-blind, active-controlled, randomised, non-inferiority, phase 3 trial
Source: Lancet Glob Health. 2024 Apr 11;12(5):e826–37. doi: 10.1016/S2214-109X(24)00059-7 (PMC11027156; doi:10.1016/S2214-109X(24)00059-7)
Supplement: Supplementary appendix 2 [file mmc2.pdf]

THE LANCET
Global Health

Supplementary appendix 2

This appendix formed part of the original submission and has been peer reviewed. We post it as supplied by the authors.

Supplement to: Song KR, Chapagain RH, Tamrakar D, et al. Safety and immunogenicity of the Euvichol-S oral cholera vaccine for prevention of *Vibrio cholerae* O1 infection in Nepal: an observer-blind, active-controlled, randomised, non-inferiority, phase 3 trial. *Lancet Glob Health* 2024; **12:** e826–37.

Study protocol

A Phase III, Multicenter, Observer-Blinded, Randomized, Active Controlled Trial to Evaluate Immune Non-Inferiority, Safety and Lot-to-Lot Consistency of Oral Cholera Vaccine-Simplified Compared to Shanchol^{TM} in 1 to 40 years old Healthy Nepalese Participants

Protocol Number: OCV-S

Sponsor: International Vaccine Institute
SNU Research Park, 1 Gwanak-ro,
Gwanak-gu, Seoul, 08826
Republic of Korea

Co-sponsor EuBiologics Co., Ltd.
8F, 207 Dosan-Daero, Gangnam-gu, Seoul,
06026Republic of Korea

Funding Agency: Bill and Melinda Gates Foundation
Seattle, WA, USA

Version Number, Date: Version 5.0, 6 Jul 2021

Version History

Version Number	Date
Version 1.0	8 Jan 2021
Version 2.0	23 Feb 2021
Version 3.0	22 Apr 2021
Version 4.0	30 Jun 2021
Version 5.0	6 Jul 2021

FOR OFFICIAL USE ONLY
Information and data included in this document contain privileged and/or proprietary information, which is the property of the International Vaccine Institute, EuBiologics Co., Ltd., and participating clinical trial sites in Nepal, and may not be reproduced, published or disclosed to others without written authorization. These restrictions on disclosure will apply equally to all future information, which is indicated as privileged or proprietary.

TABLE OF CONTENTS

TABLE OF CONTENTS 2
LIST OF ABBREVIATIONS 6
LIST OF TABLES 8
1. PROTOCOL SUMMARY 10
1.1 Synopsis 10
1.2 Table of Study Procedure/Schedule Of Events 17
2. INTRODUCTION 18
2.1 Background 18
2.2 Cholera vaccines 20
3. TEST VACCINES 23
3.1 Preclinical data 23
3.2 Clinical Data 23
3.3 Potential Risks and Benefits 23
3.3.1 Known Potential Risks 23
3.3.2 Known Potential Benefits 24
3.4. STUDY RATIONALE 24
4. OBJECTIVES 26
4.1 Primary objectives 26
4.2 Secondary objectives 26
4.3 Exploratory objectives 26
5. TRIAL ORGANIZATION 27
6. INDEPENDENT ETHICS COMMITTEE/INSTITUTIONAL REVIEW BOARD 28
7. STUDY DESIGN AND CLINICAL PROCEDURES 29
7.1 Description of the Overall Trial Design and Plan 29
7.1.1 Trial Design 29
7.1.2 Trial Plan 30
7.1.3 Visit procedures 31
7.2 Enrollment and Retention of Study Population 38
7.2.1 Recruitment Procedures 38
7.2.2 Informed Consent Procedures and Documentation 39
7.2.3 Compensation for participation 40
7.2.4 Pregnancy Prevention Counseling on Female Participants of Childbearing Potential 40
7.2.5 Eligibility Criteria 40
7.2.6 Medical History 42
7.2.7 Contraindications for primary and Subsequent Vaccinations 42

7.2.8 Participant Discontinuation/Withdrawal From the study 43
7.2.9 Handling Of Participant Discontinuation Or Termination 44
7.2.10 Lost to Follow-up 44
7.2.11 Discontinuation From Vaccination Phase In Case Of Pregnancy 45
7.2.12 Classification of Participants Who Did Not Complete the Trial or the Vaccination Phase 45
7.2.13 Follow-up of Participants Who Did Not Complete the Trial or the Vaccination Phase 46
7.2.14 Follow-up and Reporting of Pregnancies 46
7.2.15 Protocol Deviations 47
7.3 PROTOCOL AMENDMENT 48
7.4 PREMATURE TERMINATION OR SUSPENSION OF STUDY 49
7.5 END OF STUDY 49

8. INVESTIGATIONAL PRODUCT AND CONTROL DESCRIPTION 51

8.1 IDENTITY OF THE INVESTIGATIONAL PRODUCT: OCV-S 51
8.1.1 Composition 51
8.1.2 Preparation of Administration 51
8.2 IDENTITY OF CONTROL PRODUCT: SHANCHOL™ 52
8.2.1 Composition 52
8.2.2 Preparation of Administration 52
8.3 PRODUCT LOGISTICS 53
8.3.1 Labelling and Packaging 53
8.3.2 Product Shipment, Storage, Stability and Accountability 53
8.3.3 Product Preparation 55
8.3.4 Replacement Doses 55
8.3.5 Disposal of Unused Products 56
8.3.6 Recall of Products 56
8.4 RANDOMIZATION AND ALLOCATION PROCEDURES 56
8.5 BLINDING 56
8.6 UNBLINDING OF PARTICIPANTS 57
8.7 TREATMENT COMPLIANCE 58
8.8 CONCOMITANT MEDICATION 58

9. LABORATORY PROCEDURES/EVALUATIONS 59

9.1 LABORATORY EVALUATIONS 59
9.2 SPECIMEN PROCESSING, HANDLING, AND STORAGE 59
9.3 SPECIMEN SHIPMENT 60
9.4 ASSESSMENT OF IMMUNOGENICITY 60

10. ASSESSMENT OF SAFETY 62

10.1 SAFETY ASSESSMENT 62
10.1.1 Defination of Adverse Events 62
10.1.2 Definition of Serious Adverse Events 63
10.1.3 Definition of Suspected Unexpected Serious Adverse Event 63
10.1.4 Definition of Unanticipated Problems 64
10.2 CLASSIFICATION OF AN ADVERSE EVENT 64

10.2.1 Severity of Event ... 64
10.2.2 Relationship to Investigational Product ... 65
10.2.3 Expectedness ... 66
10.3 Time Period and Frequency for Event Assessment and Follow-Up ... 66
10.4 Reporting Procedures ... 67
10.4.1 Adverse Event Recording and Reporting ... 67
10.4.2 Serious Adverse Event Reporting ... 68
10.4.3 Safety Oversight ... 69

11. STUDY MONITORING ... 71

12. QUALITY ASSURANCE AND QUALITY CONTROL ... 73

13. STATISTICAL CONSIDERATIONS ... 74

13.1 Study Endpoints ... 74
13.1.1 Primary Immunogenicity Endpoint ... 74
13.1.2 Primary Safety Endpoint ... 74
13.1.3 Secondary Endpoints ... 74
13.1.4 Exploratory Endpoint ... 74
13.2 Sample Size ... 75
13.3 Statistical Analysis Plan ... 75
13.4 Statistical Hypotheses ... 76
13.5 Analysis Datasets ... 77
13.6 Description of Statistical Methods ... 77
13.6.1 General Approach ... 77
13.6.2 Baseline Descriptive Statistics ... 78
13.6.3 Primary Immunogenicity Endpoint Analysis ... 78
13.6.4 Primary Safety Endpoints Analysis ... 78
13.6.5 Secondary Endpoints Analysis ... 79
13.6.6 Adherence and Retention Analyses ... 79
13.6.7 Planned Interim Analysis ... 80
13.6.8 Additional Sub-Group Analysis ... 80
13.6.9 Multiple Comparison/Multiplicity ... 80
13.6.10 Exploratory Analyses ... 80

14. SOURCE DOCUMENTS AND ACCESS TO SOURCE DOCUMENTS ... 81

15. DATA HANDLING AND RECORD KEEPING ... 82

15.1 Data Collection and Management Responsibilities ... 82
15.2 Study Records Retention ... 82
15.3 Publication and Data Sharing Policy ... 83

16. ETHICS/PROTECTION OF HUMAN PARTICIPANTS ... 84

16.1 Regulatory and Ethical Compliance ... 84
16.2 Participant and Data Confidentiality ... 84
16.3 Research Use of Stored Human Samples ... 85

16.4 FUTURE USE OF STORED SPECIMENS 86

17. REFERENCES 87

18. APPENDICES 90

18.1 APPENDIX I. GRADING SCALE OF ADVERSE EVENT 90

LIST OF ABBREVIATIONS

AE	Adverse Event
AF	Assent Form
ANOVA	Analysis of Variance
BS	Blood Sample
°C	Degree Celsius
CI	Confidence Interval
CIOMS	Council for International Organizations of Medical Sciences
COG	Cost of goods
CONSORT	Consolidated Standards of Reporting Trials
CMP	Clinical monitoring plan
CRF	Case Report Form
CSR	Clinical Study Report
D	Day
DC	Diary card
DDA	Department of Drug Administration
DOB	Date of Birth
DSMB	Data Safety Monitoring Board
EC	Ethics Committee
EDC	Electronic Data Capture
eCRF	Electronic Case Report Form
ELISA	Enzyme Linked Immunosorbent Assay
FAS	Full Analysis Set
FDA	United States Food and Drug Administration
Gavi	Global Alliance for Vaccines and Immunisation
GCP	Good Clinical Practice
GMT	Geometric Mean Titer
IB	Investigator’s Brochure
IAF	Informed Assent Form
ICF	Informed Consent Form
ICH	International Council for Harmonization
IgG	Immunoglobulin G
IgM	Immunoglobulin M
IP	Investigational Product
IRB/ IEC	Institutional Review Board/ Independent Ethics Committee
IVI	International Vaccine Institute
IVR	Initiative for Vaccine Research
LAR	Legally Acceptable Representative
LEU	Lipopolysaccharide ELISA Units
LPS	Lipopolysaccharide

M	Month
MA	Marketing Authorization
MedDRA	Medical Dictionary for Regulatory Activities
MIN	Minutes
mITT	Modified Intention To Treat
mL	Milliliter
MOP	Manual of Operations and Procedures
N	Number
NA	Not Applicable
NRA	National Regulatory Agency
NHP	Non-human primates
NHRC	Nepal Health Research Council
O	Somatic
OCV-S	Oral Cholera Vaccine-Simplified
PBS	Phosphate Buffered Saline
PD	Post Dose
PI	Principal Investigator
PPS	Per Protocol Analysis Set
QA	Quality Assurance
QC	Quality Control
Q.S.(q.s.)	Quantum satis
RTSM	Randomization and Trial Supply Management system
SAE	Serious Adverse Event
SAF	Safety Analysis Set
SAGE	Strategic Advisory Group of Experts on Immunization
SI	Site investigator
SOE	Schedule of Events
SOP	Standard Operating Procedure
SUSAR	Suspected Unexpected Serious Adverse Reaction
TBD	To Be Determined
TEAEs	Treatment Emergent Adverse Events
V. cholerae	*Vibrio cholerae*
WC	Whole Cell
WHO	World Health Organization
WHO PQ	World Health Organization Prequalification

LIST OF TABLES

TABLE 1. GROUP ALLOCATION AND STUDY SAMPLE SIZE 14
TABLE 2. STUDY PROCEDURES/SCHEDULE OF EVENTS 17
TABLE 3. COMPOSITION OF WC OCV (SHANCHOL™, EUVICHOL® AND EUVICHOL-PLUS) 21
TABLE 4. SHANCHOL™/EUVICHOL-PLUS AND OCV-S FORMULATION COMPARISON 21
TABLE 5. BLOOD SAMPLE VOLUME BY AGE 32
TABLE 6. ASSESSMENT METHOD OF SOLICITED GENERAL ADVERSE EVENTS 34
TABLE 7. ASSESSMENT METHOD OF UNSOLICITED ADVERSE EVENTS 37
TABLE 8. GRADING SCALE OF AE: CLINICAL ABNORMALITIES 90

LIST OF FIGURES

Figure 1. Schedule of Events 15

1. PROTOCOL SUMMARY

1.1 SYNOPSIS

**Name of the Sponsor:** International Vaccine Institute (IVI) and EuBiologics Co., Ltd.	
**Name of Investigational Product:** Oral Cholera Vaccine-Simplified	
**Name of Active Ingredients:** Formalin inactivated *Vibrio cholerae* O1 Inaba Phil 6973, El Tor and O1 Ogawa Cairo 50, Classical biotypes	
**Title of Study:** A phase III, Multicenter, Observer-Blinded, Randomized, Active Controlled Trial to Evaluate Immune Non-Inferiority, Safety and Lot-to-Lot Consistency of Oral Cholera Vaccine-Simplified (OCV-S) compared to Shanchol™ in 1 to 40 years old Healthy Nepalese Participants	
**Protocol Number:** OCV-S	
**Study Sites and Principal Investigators:**<br>• Kanti Children's Hospital, Kathmandu: Dr Ram Hari Chapagain<br>• School of Medical Sciences, Kathmandu University, Dhulikhel: Dr Dipesh Tamrakar<br>• B. P. Koirala Institute of Health Sciences, Dharan: Dr Shipra Chaudhary<br>• Nepalgunj Medical College, Nepalgunj: Dr Piush Kanodia	
**Study Period (years/months):**<br>Estimated date first participant enrolled: July 2021<br>Estimated duration of the trial: 12.5 months	**Phase of Development:** III
**Study Hypothesis:**<br>The scientific rationale in this protocol is to establish non-inferior immunogenicity of OCV-S to Shanchol™ in healthy children and adults. The study is intended to compare the proportion of participants showing seroconversion against *Vibrio cholerae* O1 Inaba and Ogawa 2 weeks after the second dose of OCV-S to Shanchol™ for ages 1 to 40 years old. The sample size calculation is based on the non-inferiority hypothesis of the immunogenicity endpoint and the lot-to-lot consistency endpoint, for primary and secondary objectives, respectively. Safety assessment of OCV-S will evaluate safety profile in all age strata combined and describe the safety profile in each age stratum at 7 days and 28 days after each dose.	
**Objectives:**<br><br>**Primary Objectives**<br>• To demonstrate non-inferiority of OCV-S compared to Shanchol™ as measured by seroconversion rates of anti-*V. cholerae* O1 Inaba and anti-*V. cholerae* O1 Ogawa vibriocidal titer 2 weeks after second dose for all ages (*i.e.*, one lot of OCV-S)<br>• Assess and compare safety profile in all age strata combined and describe the safety profile in each age stratum 7 days and 28 days after each dose	

Secondary Objectives

- To demonstrate non-inferiority of OCV-S compared to Shanchol™ as measured by GMT of vibriocidal titers against *V. cholerae* O1 Inaba and *V. cholerae* O1 Ogawa 2 weeks after second dose (*i.e.*, one lot of OCV-S) for all ages
- To demonstrate non-inferiority of OCV-S compared to Shanchol™ as measured by seroconversion rates of vibriocidal titers against *V. cholerae* O1 Inaba and *V. cholerae* O1 Ogawa 2 weeks after second dose (*i.e.*, one lot of OCV-S) in each age stratum
- To demonstrate non-inferiority of OCV-S compared to Shanchol™ as measured by GMT of vibriocidal titers against *V. cholerae* O1 Inaba and *V. cholerae* O1 Ogawa at 2 weeks after second dose (*i.e.*, one lot of OCV-S) in each age stratum
- To demonstrate the equivalence of immunogenicity as measured by GMT of vibriocidal titers against *V. cholerae* O1 Inaba and *V. cholerae* O1 Ogawa of 3 lots of OCV-S 2 weeks after second dose in adults

Exploratory Objectives

- To describe the difference of immunogenicity as measured by seroconversion rates of vibriocidal titers against *V. cholerae* O1 Inaba and *V. cholerae* O1 Ogawa of 3 lots of OCV-S 2 weeks after second dose in adults
- To describe vibriocidal antibody responses 2 weeks after first dose for all ages and for each age stratum

Methodology: This is a multicenter, randomized, observer-blinded, active controlled study to evaluate immune non-inferiority and safety of OCV-S compared to Shanchol™ in 1 to 40 years old Healthy Nepalese Participants. Lot-to-Lot consistency will be included as a secondary objective.

The study vaccines will be administered to 2,530 healthy participants. 935 healthy participants will receive a comparator vaccine (Shanchol™) and 1,595 participants will be vaccinated with an investigational vaccine (OCV-S). Each of OCV-S and Shanchol™ will be vaccinated twice 2 weeks apart. Participants will be followed up for 2 weeks after each dose for immunogenicity (blood samples at Day 0, Day 14 prior to the first and second vaccinations respectively, and Day 28) and for 24 weeks after the second dose for safety assessment. The primary objective is to demonstrate non-inferiority of OCV-S to Shanchol™ as measured by seroconversion rates of vibriocidal titers against *V. cholerae* O1 Inaba and *V. cholerae* O1 Ogawa 2 weeks after second dose for all ages. The secondary objectives are to demonstrate non-inferiority of OCV-S compared to Shanchol™ as measured by GMT and seroconversion rates of vibriocidal titers against *V. cholerae* O1 Inaba and *V. cholerae* O1 Ogawa 2 weeks after second dose for all ages and in each age stratum. The equivalence of immunogenicity will be demonstrated as measured by GMT and seroconversion rates of vibriocidal titers against *V. cholerae* O1 Inaba and *V. cholerae* O1 Ogawa of 3 lots of OCV-S 2 weeks after second dose in adults. Safety profile will be assessed and compared in all age strata combined and described in each age stratum 7 days and 28 days after each dose.

Participants will be observed at the study site for 30 minutes after vaccination for safety assessment. Solicited adverse events will be recorded during 7 days after each vaccination. Unsolicited adverse events will be recorded during 28 days after each vaccination. Serious adverse events will be reported during the entire study period.

Blood samples will be collected at baseline prior to vaccination and at 2 weeks post each dose vaccination for immunogenicity assessment (Day 0, Day 14, and Day 28).

Eligible participants enrolled in the study will be randomized into 4 study groups with age stratum of 1 to 5 years old, 6 to 17 years old, and 18 to 40 years old. Participants will be observed at the study site for 30 minutes after vaccination for immediate safety assessment. Solicited adverse events will be recorded during 7 days after vaccination. Unsolicited adverse events will be recorded within 28 days after vaccination. Serious adverse events will be reported during the entire study period.

Except for designated study site personnel responsible for vaccine administration, site investigators, study nurse, and those assessing clinical outcomes, and data analysts will be blinded to vaccine allocation until data base lock for the statistical analysis.

Estimated Number of Participants to Enroll: A total of 2,530 participants aged 1 to 40 years will be enrolled in this study. 2,530 participants will be randomized into 4 groups, 330 adult participants in group A and B each and 935 adult and children participants in group C and D each. Group C and D will be divided into age stratum of 1 to 5 years old, 6 to 17 years old, and 18 to 40 years old. Participants in the group A, B and C will receive OCV-S while participants in group D will receive Shanchol™. Group A, B and C are three different lots of OCV-S and group D is Shanchol™.

Criteria for Inclusion/Exclusion:

Inclusion Criteria

To be eligible to participate in this study, any individual must meet the following criteria:

1. Healthy participants 1 to 40 years of age at enrollment
2. Participants/Parent(s)/LAR willing to provide written informed consent to participate study voluntarily
3. Participants/Parent(s)/LAR who can be followed up during the study period and can comply with the study requirements

Exclusion Criteria

An individual who meets any of the following criteria will be excluded from participation in this study:

1. Known history of hypersensitivity reactions to other preventive vaccines
2. Severe chronic diseases or medical conditions based on the medical judgment of the investigator. In particular, a participant with a) chronic infection such as tuberculosis, or sequel of poliomyelitis, b) known history of immune function disorders, c) chronic use of systemic steroids (>2 mg/kg/day or >20 mg/day prednisone equivalent for periods exceeding 10 days)/cytotoxic drugs/immunosuppressants within past 6 weeks, d) active malignancy with the exception of adequately treated basal cell or squamous cell skin

cancer, in situ cervical cancer, adequately treated Stage I cancer from which the participant is currently in complete remission, or any other cancer from which the participant has been disease-free for 5 years, e) congestive heart failure, f) myocardial infarction within the previous 6 months, g) known HIV-infected patients, h) neurological and/or psychiatric disorder, or i) known history of uncontrolled coagulopathy or blood disorders

3. Participant who received any other vaccines within 4 weeks prior to enrollment in OCV-S study or who plans to receive any vaccine within 4 weeks after the second dose of study vaccine administration
4. Participant concomitantly enrolled or scheduled to be enrolled in another trial
5. Receipt of blood or blood-derived products in the past 3 months
6. Participant who has previously received a cholera vaccine
7. Any female participant who is lactating, pregnant or planning for pregnancy during study period
8. Participants planning to move from the study area before the end of study period
9. Employees or the family members of the OCV-S study sites

Temporary Contraindication

Should a participant have one of the conditions/situations listed below, the Investigator will postpone primary or subsequent vaccination until the condition/situation is resolved.

1. Febrile illness (axillary temperature ≥ 38°C) or moderate or severe acute illness/infection on the day of vaccination or planned vaccination, according to Investigator's judgment.
2. Gastrointestinal symptoms including nausea, vomiting, or decreased appetite within 24 hours prior to trial vaccination.
3. Administration of antidiarrheal drugs or antibiotics to treat diarrhea or abdominal pain lasting 2 weeks or longer within 6 months prior to trial vaccination
4. Diarrhea occurring up to 1 week within 6 months prior to trial vaccination.
5. Receipt of any vaccine in 4 weeks preceding the trial vaccination.

*Lactation: Breastfeeding women will not be enrolled. Should a female participant decide to breastfeed during the vaccination period, she will be excluded from further vaccination, but will be followed for safety until the end of the study

**Pregnancy Test is necessary for all married female participants of childbearing age. If un-married female participants of childbearing age agree to take pregnancy test, the test should be performed.

Investigational Product, Dosage and Mode of Administration:

Test Vaccine

Oral cholera vaccine with formalin inactivated *Vibrio cholerae* O1 Inaba Phil 6973, El Tor and O1 Ogawa Cairo 50, Classical biotypes, manufactured by EuBiologics Co., Ltd. in Republic of Korea.

- Dose formulation: Formalin inactivated O1 Inaba Phil 6973 El Tor biotype (900 LEU*) and O1 Ogawa Cairo 50 Classical biotype (600 LEU) per dose
- Mode of administration: oral administration of 1.5 mL per dose

- Number of doses: 2 doses with 2 weeks interval
- Storage conditions: +2 to +8°C

  * LEU: Lipopolysacharide ELISA Units

Active Control Vaccine

Locally available Shanchol™ manufactured by Sanofi Healthcare India Private Limited.

- Dose formulation: Formaldehyde inactivated O1 Inaba Phil 6973 El Tor biotype (600 LEU), O1 Ogawa Cairo 50 Classical biotype (300 LEU) and O139 4260B biotype (600 LEU), and heat inactivated O1 Ogawa Cairo 50 Classical biotype (300 LEU) and O1 Inaba Cairo 48 Classical biotype (300 LEU) per dose
- Mode of administration: oral administration of 1.5mL per dose
- Number of doses: 2 doses with 2 weeks interval
- Storage conditions: +2 to +8°C

Group Allocation

Table 1. Group Allocation and Study Sample Size

Group	Age Strata (year old)	Number of participants		Day 0	Day 14
		By age stratum	Total		
A	18-40	330	330	OCV-S 1st dose	OCV-S 2nd dose
	6-17	-			
	1-5	-			
B	18-40	330	330		
	6-17	-			
	1-5	-			
C	18-40	330	935		
	6-17	360			
	1-5	245			
D	18-40	330	935	Shanchol™ 1st dose	Shanchol™ 2nd dose
	6-17	360			
	1-5	245			
Total			2,530		

- Group A, B and C are three different lots of OCV-S and group D is Shanchol™.
- Non-inferiority of OCV-S and Shanchol™ will compare in Group C and D.
- Lot-to-lot consistency of OCV-S will compare Group A and B, B and C, and C and A.
- All groups will be included in safety analysis.

Schedule of Events

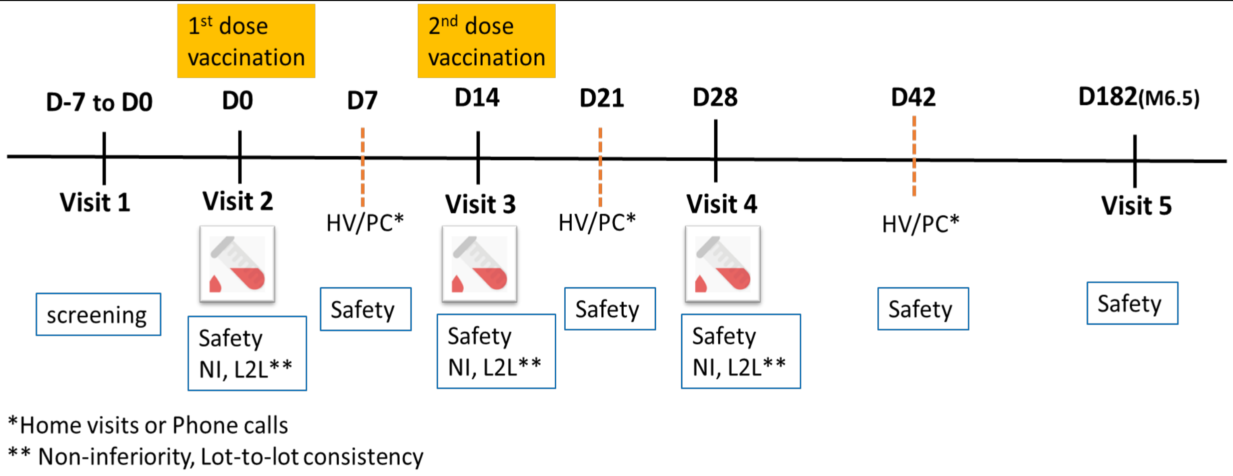

Figure 1. Schedule of Events

- 5 site visits include screening visit, 2 doses of vaccinations and 3 blood samples for immune non-inferiority and lot-to-lot consistency, and safety follow up.
- 3 safety follow-ups via home visit or phone call.

Criteria for Evaluation

Primary endpoints

- Proportion of participants showing seroconversion of vibriocidal titers against *Vibrio cholerae* O1 Inaba and O1 Ogawa (seroconversion is defined as at least 4-fold increase of vibriocidal titers against *V. cholerae* O1 Inaba and O1 Ogawa compared to baseline) at 2 weeks after second dose of either OCV-S (*i.e.*, one lot of OCV-S) or Shanchol™ for all ages
- Frequency of solicited adverse events within 7 days post vaccination, unsolicited adverse events within 28 days post vaccination, SAEs after each dose during the entire study period in all ages and in each age stratum

Secondary endpoints

- Geometric Mean Titer of vibriocidal antibodies against *Vibrio cholerae* O1 Inaba and Ogawa 2 weeks after second dose of either OCV-S (*i.e.*, one lot of OCV-S) or Shanchol™ for all ages
- Proportion of participants showing seroconversion against *Vibrio cholerae* O1 Inaba and Ogawa 2 weeks after second dose of either OCV-S (*i.e.*, one lot of OCV-S) or Shanchol™ in each age stratum
- Geometric Mean Titer of vibriocidal antibodies against *Vibrio cholerae* O1 Inaba and Ogawa 2 weeks after second dose of either OCV-S (*i.e.*, one lot of OCV-S) or Shanchol™ in each age stratum
- Geometric Mean Titer of vibriocidal antibodies against *Vibrio cholerae* O1 Inaba and Ogawa 2 weeks after second dose of 3 lots of OCV-S in adults

Exploratory endpoints

- Proportion of participants showing seroconversion of vibriocidal titers against *Vibrio cholerae* O1 Inaba and Ogawa 2 weeks after second dose of 3 lots of OCV-S in adults
- Seroconversion rate and GMT of vibriocidal antibodies 2 weeks after first dose of either OCV-S or Shanchol^{TM} for all ages and for each age stratum

1.2 TABLE OF STUDY PROCEDURE/SCHEDULE OF EVENTS

Table 2. Study Procedures/Schedule of Events

Visit Number	V1	V2	HV/PC*	V3	HV/PC*	V4	HV/PC*	V5	Unscheduled Visit
Visit Day	-7 to 0	0	7†	14	21††	28†††	42††††	182†††††	
Visit Week	-1 to 0	0	1	2	3	4	6	26	
Visit window (Days)	NA	NA	±1	+3	±1	±3	±1	±5	
Screening	X								
Informed Consent/Assent	X								
Participant Eligibility assessment	X	X							
Demographic Information	X								
Height / Weight	X								
Medical history	X	X		X					X
Prior/Concomitant Medication	X	X	X	X	X	X	X	X***	X
Physical Examination	X	X		X					X
Vital Signs	X	X		X					X
Urinary Pregnancy Test**	X	X		X					
Pregnancy Prevention Counseling	X								
Enrollment and Randomization	X	X							
Blood Samples		X		X		X			
Vaccine Administration		X		X					
Post Vaccination 30 minutes Observation		X		X					
Diary Card (DC) Distribution		DC1	DC2	DC3	DC4	DC5	DC6		
Diary Card (DC) Collection			DC1	DC2****	DC3	DC4****	DC5	DC6	
Solicited Adverse Events			X		X				
Unsolicited Adverse Events		X	X	X	X	X	X		X
Study Completion Form								X	
SAE/Pregnancy Reporting	To be reported at any time throughout the study period								

† 7 days post 1st dose vaccination
†† 7 days post 2nd dose vaccination
††† 28 days post 1st dose vaccination
†††† 28 days post 2nd dose vaccination
††††† 6 months post 2nd dose vaccination

* Home visits/Phone calls
** Pregnancy Test is necessary for all married female participants of childbearing age. If un-married female participants of childbearing age agree to take pregnancy test, the test should be performed.
*** After D42, concomitant medication history related to SAEs only will be recorded
**** For safety follow ups through phone calls, DC1,2 and DC 3,4 will be collected on V3 and V4, respectively.

2. INTRODUCTION

2.1 BACKGROUND

Cholera is an acute diarrheal disease caused by ingestion of toxigenic serogroups of *V. cholerae* and is endemic in parts of south and south-east Asia and in Africa. The disease is transmitted primarily by drinking water or eating food that is contaminated by the feces of a cholera infected person. Approximately 1.3 billion people are at risk of cholera in endemic countries. An estimated 2.86 million cholera cases (uncertainty range 1.3–4.0 million) occur annually in endemic countries. Among these cases, there are an estimated 95,000 deaths (uncertainty range: 21,000– 43,000).[1,2] However, it is difficult to determine the exact number of cases and deaths as many countries do not disclose their data due to the concern that acknowledging cholera transmission may have a negative impact on tourism and trade of agricultural products. Cholera was not observed in the *Americas* during most of the 20th century, however, it reappeared towards the end of the century and has persisted in some settings such as Haiti.[3,4]

The main symptoms of cholera are acute, profuse watery diarrhea of one or a few days' duration with or without vomiting. Within 3-4 hours of onset of symptoms, a healthy person may become severely dehydrated and may die within 24 hours if proper treatment is not given immediately. The diagnosis of cholera is commonly established by isolating the causative organism from the stools of infected individuals. Agglutination tests with specific antisera are generally used for confirming the infection. The primary treatment of cholera is to give Oral Rehydration Solution (ORS) containing salts and glucose to replace water and electrolytes. If the patient has severe dehydration or does not recover quickly with ORS, intravenous rehydration treatment (normal saline or sodium lactate) is needed. Antibacterial drugs (tetracycline or doxycycline) are beneficial in the case of severe condition to shorten its duration and severity.[1]

V. cholerae is a Gram-negative, rod-shaped bacterium. More than 200 serogroups of this bacterium are available in nature, and serogrouping is based on the endotoxin of the somatic (O) antigen of *V. cholerae*. The endotoxin is a lipopolysaccharide (LPS) molecule, where the lipid portion of LPS is embedded in the cell wall and the polysaccharide referred to as the O-specific polysaccharide (OSP) extends out from the surface of the bacteria. The disease is caused mainly by serogroups of O1 and occasionally by O139 of *V. cholerae*. O1 strain has been divided into two biotypes, classical and El Tor. The classical biotype was discovered during the cholera

outbreaks in India and has been responsible for the previous six pandemics in modern history. However, El Tor is responsible for the seventh pandemic that started in 1961 and continues till today.[1,2] The biotypes represent variants that harbor some genotypic and phenotypic differences, and both Inaba and Ogawa serotypes can have either a classical or El Tor biotype. The classical biotype of the sixth pandemic is generally thought to be more virulent causing higher rates of severe disease, whereas the El Tor biotype has collected elements which favor ecological fitness and persistence in aquatic environments. The biotype designation is unrelated to OSP structure and cannot be characterized serologically.[3,4]

V. cholerae O1 strains are further divided into two serotypes (subtypes), Ogawa and Inaba based on their phenotypic differences in O1 antigen. The O1 polysaccharide is made up by repeating units of a perosamine. In the case of Ogawa serotype, a single 2-*O*-methyl group is available in its terminal perosamine unit, which is absent in Inaba serotype. The Inaba strains have been shown to be the mutants of wild-type Ogawa strains that have no ability to methylate the terminal perosamine. However, O1 strains have been shown to shift between these two serotypes. This interconversion is usually irreversible in the laboratory and occurs more frequently in the direction of Ogawa to Inaba.[1,2]

In 1992, a new variant of *V. cholerae* strain (named as O139 Bengal) caused extensive epidemics in Bangladesh and India, and subsequently in other parts of south Asia. This strain is a genetic derivative of the El Tor biotype in which the O1 biosynthetic genes are replaced by the O139 biosynthetic genes. Although there was initially concern that O139 would become a new pandemic strain and spread globally, after its initial explosive appearance it has remained only an occasional isolate in south Asia.

During 2010–2017, cholera continued to be a significant problem globally, with large epidemics, such as those experienced in Haiti and Yemen, and surges in endemic disease in areas of sub-Saharan Africa and Asia. While epidemic cholera attracts attention and accounts for most of the cases reported to WHO each year, endemic cholera continues to be present in large parts of sub-Saharan Africa, south and south-east Asia, as well as Haiti. Prospective population-based cholera surveillance in an African sentinel network showed an incidence of 0.3/10 000 in endemic settings that increased to <20/10 000 during epidemics. The burden of cholera is greatest in Africa and southern Asia, with about 99% of all cases occurring in these regions.[1]

While improvement of water, sanitation, and hygiene is the definitive long-term solution for the prevention of most diarrheal diseases, these measures remain unavailable to large proportions of people in the world. Vaccines offer a complementary approach for cholera infection prevention and control. The availability of affordable, inactivated oral cholera vaccine (OCV) has renewed confidence that the disease may be controlled in areas where the disease poses a significant public health problem.[1]

2.2 CHOLERA VACCINES

Since *V. cholerae* colonizes on the intestinal mucosal surface without invasion of enterocytes, the protective immune response is believed to reside at the mucosal surface without a major contribution from serum antibody. Therefore, to maximize the intestinal secretory antibody response and long-lived efficacy, cholera vaccines shifted from parenteral (injectable) to oral vaccine of which antigens could be delivered directly to the mucosal surface.[2] An injectable whole-cell parenteral vaccine formerly prepared from phenol-inactivated strains of *V. cholerae* is no longer in use because of its low efficacy and adverse side-effects.[1]

At present, two OCVs, Shanchol™, Euvichol®/Euvichol-Plus have been pre-qualified by the WHO and available for public health use in endemic countries through a Gavi (Global Alliance for Vaccines and Immunisation) funded stockpile. Both Shanchol™, Euvichol®/Euvichol-Plus are killed modified whole cell (WC) bivalent (O1 and O139) vaccines without the B subunit.

Both Shanchol™, Euvichol®/Euvichol-Plus derive from technology developed at the International Vaccine Institute (IVI) and subsequently technology transferred under licensing agreements to Shantha and EuBiologics. The strains and compositions are therefore identical. Shanchol™ was first licensed in India and WHO pre-qualified based on a randomized clinical trial[5], Euvichol®/Euvichol-Plus was subsequently registered in Korea as export only and pre-qualified based on immune non-inferiority studies.[6,7]

The current modified bivalent WC OCVs consist of five components (*V. cholerae* O1 Inaba Cairo 48, O1 Inaba Phil 6973, O1 Ogawa Cairo 50, O1 Ogawa Cairo 50, and O139) of which some are chemically inactivated, and some are heat inactivated. All three OCVs produced from the IVI technology include the same cholera strains and have the same formulation of O1 and O139 components (refer Table 3); among them Euvichol-Plus is in a plastic tube presentation and currently is the predominant product in the Gavi stockpile.

Table 3. Composition of WC OCV (Shanchol™, Euvichol® and Euvichol-Plus)[8]

Strain type used	Serotype	Biotype	Method of inactivation	Abbreviated Nomenclature
V. cholerae Cairo 48	O1 Inaba	Classical	Heat	Cairo 48H
V. cholerae Phil 6973	O1 Inaba	El Tor	Formalin	Phil 6973F
V. cholerae Cairo 50	O1 Ogawa	Classical	Heat	Cairo 50H
V. cholerae Cairo 50	O1 Ogawa	Classical	Formalin	Cairo 50F
V. cholerae O139 4260B	O139	Not applicable	Formalin	O139F

The manufacturing of the current modified bivalent WC OCVs, with respect to the number of strains and using two inactivation processes, is cumbersome and imposes limitations on the cost of goods (COG) and the supply of vaccine. Preliminary analyses of the feasibility and impact of simplifying the formulation of the vaccine have indicated that this could decrease the COG by about 20% and increase vaccine production capacity by about 38%, based on the assumption of reducing the number of components to two [formalin inactivated O1 Inaba Phil 6973 (El Tor), O1 Ogawa Cairo 50 (Classical)] and with a re-balanced composition to achieve the same antigenic quantity of O1 Inaba and Ogawa, and to generate an equivalent relevant immune response to support the current label indication (*i.e.*, prevention of cholera caused by *Vibrio cholerae*), and considering removal of strain O139.

In January 2020, a technical consultation with independent cholera experts/immunologists and legacy OCV developers was held and the rationale was confirmed that a simplified formulation containing only two of the components could yield an equivalent protective effect against *Vibrio cholerae O1*. The expert group further confirmed the simplified formulation now referred to as OCV-S.

Table 4. Shanchol™/Euvichol-Plus and OCV-S formulation comparison

Vaccine Component	Inactivation method	Shanchol™/Euvichol-Plus (LEU/Dose)	OCV-S (LEU/dose)
O 139 (4260B)	Formalin	600	X

Vaccine Component	Inactivation method	Shanchol™/Euvichol-Plus (LEU/Dose)	OCV-S (LEU/dose)
O1 Inaba Cairo 48 Biotype-Classical	Heat	300	X
O1 Inaba Phil 6973 Biotype- El Tor	Formalin	600	900
O1 Ogawa Cairo 50 Biotype-Classical	Formalin	300	600
O1 Ogawa Cairo 50 Biotype-Classical	Heat	300	X
Total LEU/Dose		O1 Inaba=900<br>O1 Ogawa=600<br>Total (O1+O139) = 2100	O1 Inaba=900<br>O1 Ogawa=600<br>Total (O1) = 1500
Volume/dose		1.5 mL	

3. TEST VACCINES

3.1 PRECLINICAL DATA

There is no suitable animal model for measuring potency of orally delivered cholera vaccines.[36] Consequently, the only reliable way to assess immunogenicity of this new formulation will be directly in humans. A pre-clinical toxicology study was conducted for Euvichol®.[23] More importantly, greater than 45 million doses of Euvichol® and Euvichol-Plus have been administered globally since registration and pre-qualification without the emergence of any safety concerns and SAGE (Strategic Advisory Group of Experts on Immunization) recommends use of OCV in pregnant women at risk for cholera.[1,37] Considering that neither new antigens nor new process were added, and only removal of antigens was required for OCV-S development, there was consensus among the Expert Group that pre-clinical studies including toxicology is not required.[16,36]

3.2 CLINICAL DATA

Considering that neither new antigens nor new process were added, and only removal of antigens was required for OCV-S development, there was consensus among the Expert Group that clinical studies including phase I and II are not required.[16]

3.3 POTENTIAL RISKS AND BENEFITS

3.3.1 KNOWN POTENTIAL RISKS

Euvichol® and Euvichol-Plus are licensed, WHO pre-qualified and have been used since 2015 years and 2018 respectively with a well-established safety profile. After taking Euvichol®/Euvichol-Plus, during the first 7 days, the most frequently reported solicited adverse drug reactions in the clinical trials were headache, fever, diarrhea, nausea/vomiting and myalgia which were reported in 102 participants (3.40%) among 2,999 participants. Among adverse drug reactions examined during the period of 28 days after taking the vaccines, gastrointestinal disorders reported the highest numbers i.e., 35 participants (1.17%) among 2,999 participants.[9,10]

Since the two antigenic components in OCV-S are identical to two components in Euvichol® and Euvichol-Plus, the expected adverse reactions of OCV-S are expected to be the same and include fever, nausea/vomiting, diarrhea, headache, fatigue, myalgia and anorexia/loss of appetite. These reactions are expected to be mild or moderate in intensity and transient and resolve spontaneously without sequelae.

In all participants, potential risks may also include the unwanted effects of blood sampling (*i.e.*, the discomfort from having blood taken lasting for a few seconds to minutes). There may be chance of tenderness or bruising at the spot where blood will be drawn. Fainting or dizziness can occur also after a blood draw, but this is uncommon. On very rare occasions, infection can occur where the needle is inserted to draw blood.

3.3.2 KNOWN POTENTIAL BENEFITS

As Nepal is a cholera endemic country, study participants may receive benefit through OCV vaccination in cholera protection from study participation, but all participants in the study will be closely followed with regular visits and contacts as part of the study procedures. This protocol is not designed to provide treatment for any condition. Participants will have access to their medical records and findings of medical concern will be referred for appropriate care and treatment during the study. The potential benefits to vaccinated participants are substantial, since cholera is endemic in several parts of the world including in Nepal. According to the updated global burden of cholera in endemic countries, it was estimated that over 18 million people, more than 60% of the Nepalese population, are at risk of cholera with an incidence rate of 1.64 per 1,000 resulting in an estimated 30,379 cases annually.[11,12]

3.4. STUDY RATIONALE

Cholera is a disease of inequity that continues to disproportionately affect the world's poorest and most vulnerable people. Endemic in at least 47 countries, transmission of cholera results in an estimated 2.9 million cases and 95,000 deaths per year globally.[11] Nepal is a cholera endemic country and at high risk for outbreaks due to a steady increase in urban population density accompanied by an inadequate supply of safe drinking water and improved sanitation. Most importantly, Nepal faces flooding and landslides during the rainy season every year which often

lead to the breakdown of the already fragile water and sanitation infrastructure making the possibility of cholera outbreaks, which may be challenging to prevent and control.[13]

In 2017 the Global Task Force on Cholera Control launched an ambitious plan of "Ending Cholera: A Roadmap to 2030", advocating a change in focus from reactive use of vaccine to quell outbreaks to pre-emptive use of OCV integrated with WASH (Water, Sanitation and Hygiene) to systematically prevent outbreaks in high-risk communities.[14] As currently modelled by Gavi, the strategy could avert 66,000–659,000 deaths and 2–27 million cases by 2035 at a procurement cost of $1.3 billion.[15,16] However, this strategy requires an estimated 670 million doses of OCV. EuBiologics is the major supplier to the Stockpile, but their current production capacity is ~25 million doses per year. Shanchol™ production is limited to ~4 million doses per year and Shantha has plans to discontinue its commercial sales in 2023.[16,17,18] Although other OCV products may enter the market by 2025, the timing and production capacity are unknown. Consequently, the OCV forecast is for continued supply constraint potentially limiting achievement of the WHO objectives for 2030. As Gavi considers OCV moderately cost effective at the current price (~$1.85 per dose) it has also called for improved value for money in order to sustain or expand their investment.[16]

The manufacturing of the current modified bivalent WC OCVs includes 5 distinct components with two inactivation methods. Redundant heat and formalin inactivated components were included because the relative importance of protein versus polysaccharide antigens in inducing protection was unknown and *Vibrio cholerae* O139, a newly emergent strain at that time, was included under concern that it might become a pandemic pathogen.[16]

Preliminary analyses of the feasibility and impact of simplifying the formulation of the vaccine have indicated that this could decrease the COG by about 20% and increase vaccine production capacity by about 38%, based on the assumption of reducing the number of components to two [formalin inactivated O1 Inaba Phil 6973 (El Tor), O1 Ogawa Cairo 50 (Classical)] and with a re-balanced composition to achieve the same antigenic quantity of O1 Inaba and Ogawa, and to generate an equivalent relevant immune response to support the current label indication (*i.e.*, prevention of cholera caused by *Vibrio cholerae*), and considering removal of strain O139.[16]

4. OBJECTIVES

4.1 PRIMARY OBJECTIVES

- To demonstrate non-inferiority of OCV-S compared to Shanchol™ as measured by seroconversion rates of anti-*V. cholerae* O1 Inaba and anti-*V. cholerae* O1 Ogawa vibriocidal titer 2 weeks after second dose for all ages (*i.e.*, one lot of OCV-S)
- To assess and compare safety profile in all age strata combined and describe the safety profile in each age stratum 7 days and 28 days after each dose

4.2 SECONDARY OBJECTIVES

- To demonstrate non-inferiority of OCV-S compared to Shanchol™ as measured by GMT of vibriocidal titers against *V. cholerae* O1 Inaba and *V. cholerae* O1 Ogawa 2 weeks after second dose (*i.e.*, one lot of OCV-S) for all ages
- To demonstrate non-inferiority of OCV-S compared to Shanchol™ as measured by seroconversion rates of vibriocidal titers against *V. cholerae* O1 Inaba and *V. cholerae* O1 Ogawa 2 weeks after second dose (*i.e.*, one lot of OCV-S) in each age stratum
- To demonstrate non-inferiority of OCV-S compared to Shanchol™ as measured by GMT of vibriocidal titers against *V. cholerae* O1 Inaba and *V. cholerae* O1 Ogawa at 2 weeks after second dose (*i.e.*, one lot of OCV-S) in each age stratum
- To demonstrate the equivalence of immunogenicity as measured by GMT of vibriocidal titers against *V. cholerae* O1 Inaba and *V. cholerae* O1 Ogawa of 3 lots of OCV-S 2 weeks after second dose in adults

4.3 EXPLORATORY OBJECTIVES

- To describe the difference of immunogenicity as measured by seroconversion rates of vibriocidal titers against *V. cholerae* O1 Inaba and *V. cholerae* O1 Ogawa of 3 lots of OCV-S 2 weeks after second dose in adults
- To describe vibriocidal antibody responses 2 weeks after first dose for all ages and for each age stratum

5. TRIAL ORGANIZATION

The clinical trials in Nepal will be led by IVI, supported by EuBiologics as co-sponsor and manufacturing partner.

6. INDEPENDENT ETHICS COMMITTEE/INSTITUTIONAL REVIEW BOARD

Before the investigational product can be shipped to the investigational site and before the enrollment of the first participant, this protocol, the informed consent form (ICF), the assent form (AF), participant recruitment procedures, and any other written information to be provided to participants must be approved by, and/or receive favorable opinion from, the appropriate Independent Ethics Committee (IEC) or Institutional Review Board (IRB) and Regulatory approval.

In accordance with Good Clinical Practice (GCP) and local regulations, each site Principal Investigator and/or the Sponsor are responsible for obtaining this approval and/or favorable opinion before the start of the trial. If the protocol is subsequently amended, approval must be re-obtained for each amendment. Copies of these approvals, along with information on the type, version number, and date of document, and the date of approval, must be forwarded by the site Principal Investigator to the Sponsor together with the composition of the IEC/IRB (the names and qualifications of the members attending and voting at the meetings).

The site Principal Investigators will submit written summaries of the status of the trial to the IRB/IEC annually, or more frequently if requested. All SAEs occurring during the trial that are related to vaccination will be reported by the Investigator to the IRB/IEC, according to the IEC/IRB policy.

7. STUDY DESIGN AND CLINICAL PROCEDURES

7.1 DESCRIPTION OF THE OVERALL TRIAL DESIGN AND PLAN

7.1.1 TRIAL DESIGN

This is a multicenter, randomized, observer-blinded, controlled study to evaluate immune non-inferiority and safety of OCV-S compared to Shanchol™ in 1 to 40 years old healthy Nepalese participants. Lot-to-lot consistency will be included as a secondary objective. The vaccines will be administered to 2,530 healthy participants. 1,595 participants will be vaccinated with an investigational vaccine (OCV-S) and 935 healthy participants will receive an active control vaccine (licensed Shanchol™). Each participant will be vaccinated with either OCV-S or Shanchol™ twice by 2 weeks apart. Participants will be followed up for 2 weeks after each dose for immunogenicity (blood samples at Day 0, Day 14 prior to first and second vaccination respectively, and Day 28) and for 24 weeks after the second dose for safety assessment. The primary objective is to demonstrate non-inferiority of OCV-S to Shanchol™ as measured by seroconversion rates of vibriocidal titers against *V. cholerae* O1 Inaba and *V. cholerae* O1 Ogawa 2 weeks after second dose for all ages. The secondary objectives are to demonstrate non-inferiority of OCV-S compared to Shanchol™ as measured by GMT and seroconversion rates of vibriocidal titers against *V. cholerae* O1 Inaba and *V. cholerae* O1 Ogawa 2 weeks after second dose for all ages and in each age stratum. The equivalence of immunogenicity will be demonstrated as measured by GMT and seroconversion rates of vibriocidal titers against *V. cholerae* O1 Inaba and *V. cholerae* O1 Ogawa of 3 lots of OCV-S 2 weeks after second dose in adults. Safety profile will be assessed and compared in all age strata combined and described in each age stratum 7 days and 28 days after each dose. Exploratory objective will describe vibriocidal antibody responses 2 weeks after first dose for all ages and for each age stratum. The study has been designed to consider only one lot in the analysis of the primary immunogenicity endpoint in order to reduce the chance that a single consistency lot not meeting the criteria could result in the study not meeting the primary immunogenicity endpoint. To reduce the risk of failing the lot-to-lot consistency objective, it was designed to conduct the lot-to-lot consistency in only adults where immune responses to vaccine may have less inherent variability.

Eligible participants enrolled in the study will be randomized into 4 study groups with age stratum of 1 to 5 years old, 6 to 17 years old, and 18 to 40 years old. Participants will be observed at the study site for 30 minutes after vaccination for safety assessment. Solicited adverse events will be recorded during 7 days after vaccination. Unsolicited adverse events will be recorded during 28 days after first and second doses of vaccination. Serious adverse events will be reported during the entire study period. Blood samples will be collected at baseline prior to vaccination and at 2 weeks post each dose vaccination for immunogenicity assessment (Day 0, Day 14, and D 28).

Except for designated study site personnel responsible for vaccine administration, site investigators, study nurse, and those assessing clinical outcomes, as well as data analysts will be blinded to vaccine allocation until data base lock for the statistical analysis.

7.1.2 TRIAL PLAN

A schedule of assessments is provided in Table 2. Study Procedures/Schedule of Events.

7.1.2.1 VACCINATION

A schedule of study vaccinations is provided in Table 2. Study Procedures/Schedule of Events.

7.1.2.2 VISITS AND CONTACT

Any visit and any procedure must be done after the Informed Consent/ Assent has been obtained.

Evaluation of this investigational vaccine will include medical history, physical examination by the investigators and participant self-assessment. A summary of study procedures and schedule of events is shown in Table 2. Study Procedures/Schedule of Events.

After written consent/assent forms are obtained, screening procedures will be performed as described in the MOP. The screening process will take place during the week prior to enrollment (Days -7 to 0). The study staff will request the screened eligible participants/parent(s)/LAR to visit trial site for vaccination which will be considered as Day 0. A study participant will be enrolled if s/he meets all inclusion and none of the exclusion criteria and the participant's health status is deemed acceptable as determined by medical history, physical examination, and medical judgment of the site investigator. If participant/parent(s)/LAR agrees, screening and enrollment could be done on the same day if screening results are available. A study ID card will be provided to each enrolled participant.

Details of visit procedures are described in Section 7.1.3 Visit Procedures

7.1.2.3 BLOOD SAMPLING

At visits specified in the Schedule of Events, blood samples will be collected for lot-to-lot consistency and immune non-inferiority assessments of OCV-S compared to Shanchol™.

7.1.2.4 TOTAL DURATION

This study will last approximately 6.5 months. This duration period will be taken from the first dose vaccination to the last safety follow up. All the participants receiving OCV-S and Shanchol™ will be follow up for 6 months after the last dose until the end of the study.

7.1.3 VISIT PROCEDURES

Details of visit procedures during screening are as follows and summarized information is found in Table 2. Study Procedures/Schedule of Events.

Visit 1. Screening (Days -7 to 0)

1) Explain study objectives and procedures, risk and benefits to the participant /parent(s) /LAR.
2) Obtain written informed consent/assent from the participant/parent(s)/LAR. Written Informed consent/assent forms will be obtained prior to performing any study-specific screening or evaluations.
3) Obtain medical/medication history of last 1 year, demographics including date of birth, gender and perform physical examination including vital signs and height/weight and record in study database.
4) Perform screening procedures within 0 to 7 days prior to first vaccination
5) Check if participants meet inclusion/exclusion criteria.
6) Determine if participants are qualified to participate in this study and confirm by documenting on the medical record and into the study database. For participants who are not eligible, the reason(s) must be recorded.

7) All married female participants of childbearing age will be screened with urinary pregnancy test and whether using any contraceptive method or not. If un-married female participants of childbearing age agree to take pregnancy test, the test should be performed.
8) Pregnancy Prevention Counseling: Clinical staff should perform pregnancy prevention counseling on female participants of childbearing potential to evaluate if participants are able to meet the birth control eligibility criteria.
9) Schedule participants for vaccination visit (Visit 2) at the study center within 7 days after the screening visit. If an eligible participant does not come back for vaccination within the 7 days after the screening visit, screening procedures must be repeated.
10) Screening and enrollment visit could be performed on the same day if the screening related information can be obtained on the date of screening.
11) Participant who does not meet the criteria for participation in this trial because of fever or acute illness, may be rescreened when these conditions have resolved. Rescreened participants will be assigned the same screening number as for the initial screening.

Visit 2. Enrollment / 1st dose Vaccination / Blood drawing (Day 0)

1) Obtain medical/medication history of last 1 year and perform physical examination including vital signs and record in eCRF.
2) Perform urinary pregnancy test for all married female participants of childbearing age. If un-married female participants of childbearing age agree to take pregnancy test, the test should be performed.
3) Confirm eligibility of participant for the first dose of vaccination.
4) Perform enrollment, randomization and attribute enrollment number to participant.
5) Collect a venous blood sample prior to immunization; See Table below.

Table 5. Blood Sample Volume by Age

Age of participants	**1-5 Years**	**6-40 Years**
Blood sample volume	Approximately 4 mL	Approximately 6 mL

*Minimum volume for 1-5 years old is 3 mL.

6) Vaccinate the first dose vaccine.
7) Monitor participant for 30 minutes following vaccination as follows:
    - Clinical examination including vital signs, general physical examination before leaving the study center 30 minutes post vaccination

- Record adverse reactions (if any occurrence is there)

8) Instruct participant/parent(s)/LAR to record any adverse event at home for 7 days post immunization (from Day 0 to Day 6) in diary card (DC) 1.
9) If Day 7 visit will be through phone call, provide DC2 to record unsolicited adverse events (if any occurrence is there) during Day 7 to Day 13 and instruct participant/parent(s)/LAR how to fill in the diary card.
10) Provide a thermometer along with DC1 to record fever.
11) Schedule next home visit/phone call (HV/PC) at Day 7 and next visit to the study site at Day 14 and remind participant(s)/parent/LAR to bring the DC1 and DC2 (for PC1), if distributed, at the next visit. If HV/PC may not be applicable depending on situation (e.g., COVID-19 situation), it may be replaced by a site visit.
12) Instruct participant/parent(s)/LAR to contact investigator/study staff if needed.

Home visit/Phone call 1. Post 1st dose 1-week safety follow-up (Day 7 / Week 1 ± 1 days)

Each participant will be followed up for safety via home visit at Day 7 after the first dose of vaccination. A visiting site staff will,

1) Verify and collect recorded DC1 and confirm with participant/parent(s)/LAR.
2) Record solicited and unsolicited events and concomitant medications in DC1 in study database.
3) Provide DC2 to document any unsolicited adverse events (if any occurrence is there) during Day 7 to Day 13 before the next visit
4) Remind to bring DC2 at Visit 3 on Day 14.
5) Remind to visit the site for the second dose of vaccination at Day 14.
6) Remind the participant/parent(s)/LAR to contact investigator/ study staff if needed.

Home visit may be replaced by a phone call. Only a qualified healthcare professional (HCP) according to Nepal's definition of HCP (*i.e.*, registered physician, health assistant nurse practitioner, public health nurse, auxiliary health worker), should perform the call. A staff calling the participants will,

1) Check the participant's identification.
2) Call out all the solicited adverse events listed in DC1 to participant(s)/parent/LAR so that the adverse events can be confirmed through the call.

3) Enter the adverse events in DC1 into eCRF when DC1 is collected.
4) Instruct to document any adverse events (if any occurrence is there) during Day 7 to Day 13 in DC2 and remind to bring DC1 and DC2 at Visit 3 on Day 14.

Home visit/phone call maybe replaced by a site visit.

Each AE will be assessed according to the following method:

Table 6. Assessment method of Solicited General Adverse Events

**Assessment method**	
**Solicited General Adverse Events (Days 0-6 and Days 14-20)**	
Nausea/Vomiting, Diarrhea, Headache, Fatigue, Myalgia, Anorexia (loss of appetite)	General observation/interview/diary card
Fever	Thermometer/interview/diary card

Visit 3. 2nd dose Vaccination / Blood drawing (Day 14 / Week 2 + 3 days)

1) Obtain medical/medication history and perform physical examination including vital signs and record in the eCRF.
2) Verify and record all unsolicited AEs and concomitant medications in DC2 and record in the eCRF.
3) Perform urinary pregnancy test for all married female participants of childbearing age. If un-married female participants of childbearing age agree to take pregnancy test, the test should be performed.
4) Confirm eligibility of participant for the second dose vaccination.
5) Collect a venous blood sample prior to immunization; See Table below.

Table 5. Blood Sample Volume by Age

Age of participants	**1-5 Years**	**6-40 Years**
Blood sample volume	Approximately 4 mL	Approximately 6 mL

*Minimum volume for 1-5 years old is 3 mL.

6) Vaccinate the second dose vaccine.

7) Monitor participant for 30 minutes following vaccination as follows:
   - Clinical examination including vital signs, general physical examination before leaving the study center 30 minutes post vaccination.
   - Record adverse reactions (if any occurrence is there)
8) Instruct participant/parent(s)/LAR to record any adverse event at home for 7 days post immunization (from Day 14 to Day 20) in DC3.
9) If Day 21 visit will be through phone call, provide DC4 to record adverse events (if any occurrence is there) during Day 21 to Day 27 and instruct participant/parent(s)/LAR how to fill in the diary card.
10) Schedule next HV/PC2 at Day 21 and next visit to the study site at Day 28 and remind participant(s)/parent/LAR to bring the DC3 and DC4 , if distributed, at the next visit. If HV/PC may not be applicable depending on situation (e.g., COVID-19 situation), it may be replaced by a site visit.
11) Instruct participant/parent(s)/LAR to contact investigator/study staff if needed.

Home visit/Phone call 2. Post 2nd dose 1-week Safety follow-up (Day 21 / Week 3 ± 1 days)
Each participant will be followed up for safety via home visit at Day 21. A visiting site staff will,

1) Verify and collect recorded DC3 and confirm with participant/parent(s)/LAR.
2) Record solicited, unsolicited AEs and concomitant medications since last visit in eCRF.
3) Provide DC4 to document any unsolicited AE (if any occurrence is there) during Day 21 to Day 27 before the next visit.
4) Remind to bring DC4 at the next visit.
5) Remind to visit the site at Day 28.
6) Remind to contact investigator/ study staff if needed.

Home visit may be replaced by a phone call. Only a qualified healthcare professional (HCP) according to Nepal's definition of HCP (*i.e.*, registered physician, health assistant nurse practitioner, public health nurse, auxiliary health worker), should perform the call. A staff calling the participants will,

1) Check the participant's identification.
2) Call out all the solicited and unsolicited AEs listed in DC3 to participant(s)/parent/LAR so that the AEs can be confirmed through the call.

3) Enter the solicited, unsolicited AEs and concomitant medications into the study database when DC3 is collected.
4) Instruct participant(s)/parent/LAR to document any adverse events (if any occurrence is there) during Day 21 to Day 27 in DC4, provided on Day 14, and remind to bring DC3 and DC4 at Visit 4, Day 28.

Home visit/phone call maybe replaced by a site visit.

Each AE will be assessed according to the following method:

Table 6. Assessment method of Solicited General Adverse Events

Assessment method	
**Solicited General Adverse Events (Days 0-6 and Days 14-20)**	
Nausea/Vomiting, Diarrhea, Headache, Fatigue, Myalgia, Anorexia (loss of appetite)	General observation/interview/diary card
Fever	Thermometer/interview/diary card

Visit 4. Post 1st dose 4-week Safety follow up / Blood drawing (Day 28 / Week 4 ± 3 days)
Each participant will be followed up for safety and blood drawing.

1) Verify and collect DC4 and confirm with participant/parent(s)/LAR enter any unsolicited events and concomitant medications CRF
2) Provide DC5 to document unsolicited adverse events (if any occurrence is there) from Day 28 to Day 41. Instruct participant/parent(s)/LAR how to fill in the diary card.
3) If Day 42 visit will be through phone call, provide DC6 to record adverse events (if any occurrence is there) during Day 42 to Day 181.
4) Schedule next HV/PC3 at Day 42 and next visit to the study site at Day 182 and remind participant(s)/parent/LAR to bring the DC5 and DC6, if distributed, at the next visit. If HV/PC may not be applicable depending on situation (e.g., COVID-19 situation), it may be replaced by a site visit.
5) Instruct participant/parent(s)/LAR to contact investigator/study staff if needed.
6) Collect a venous blood sample prior to immunization; See Table below.

Table 5. Blood Sample Volume by Age

Age of participants	**1-5 Years**	**6-40 Years**
Blood sample volume	Approximately 4 mL	Approximately 6 mL

*Minimum volume for 1-5 years old is 3 mL.

Each AE will be assessed according to the following method:

Table 7. Assessment Method of Unsolicited Adverse Events

**Assessment method**	
**Unsolicited Adverse Events (Days 0-28 and Days 14-42)**	
Any other symptoms	General observation/interview/diary card

Home visit/Phone call 3. Post 2nd dose 4-week Safety follow-up (Day 42 / Week 6 ± 1 days)

Each participant will be followed up for safety via home visit at Day 42. A visiting site staff will,

1) Verify and collect recorded DC5 and confirm with participant/parent(s)/LAR.
2) Record unsolicited AEs since last visit in eCRF. After Day 42, record concomitant medication history related to SAEs only.
3) Provide DC6 to document any adverse events (if any occurrence is there) during Day 42 to Day 181 before the next visit
4) Remind to bring DC6 at the next visit.
5) Remind to visit the site at Day 182.
6) Remind to contact investigator/ study staff if needed.

Home visit may be replaced by a phone call. Only a qualified healthcare professional (HCP) according to Nepal's definition of HCP (*i.e.,* registered physician, health assistant nurse practitioner, public health nurse, auxiliary health worker), should perform the call. A staff calling the participants will,

1) Check the participant's identification.
2) Call out all the unsolicited AEs listed in DC5 to participant(s)/parent/LAR so that the adverse events can be confirmed through the call.

3) Enter the unsolicited AEs and concomitant medications in DC5 into the eCRF when DC5 is collected.
4) Instruct participant(s)/parent/LAR to document any adverse events (if any occurrence is there) during Day 42 to Day 181 in DC6 and remind to bring DC5 and DC6 at Visit 5, Day 182.

Home visit/phone call maybe replaced by a site visit.

Each AE will be assessed according to the following method:

Table 7. Assessment Method of Unsolicited Adverse Events

**Assessment method**	
**Unsolicited Adverse Events (Days 0-28 and Days 14-42)**	
Any other symptoms	General observation/interview/diary card

Visit 5. Post 2nd dose 24 week/6 months Safety follow up (Day 182 / Week 26 ± 5 days)

Each participant will be followed up for safety.

1) Verify and collect recorded DC5 and DC6 and confirm with participant/parent(s)/LAR.
2) Record DC5 and DC6 in study database.
3) Only SAE and concomitant medication will be collected in the eCRF.
4) Instructed to contact investigator/study staff if needed.
5) Ensure source documents are completed before the participant/parent/LAR leave the study site.
6) For unresolved AEs, participants will be continued to be monitored after study ends.

7.2 ENROLLMENT AND RETENTION OF STUDY POPULATION

7.2.1 RECRUITMENT PROCEDURES

The targeted number of participants is 2,530. Study staff will approach participants/parents/LAR between Days -7 to 0 at the health facility. The enrollment venue is the clinical trial sites in Nepal. Participants/Parents/LAR with aged 1 to 40 years visiting health centers for regular immunizations or medical check-up who may be interested in participating in the study, will be asked to go to clinical trial site during the recruitment period. Beside that healthy

volunteers from the community will be mobilized to trial sites for recruitment with the help of field health workers.

Details of visit procedures during enrollment are described in Section 7.1.3 Visit Procedures

7.2.2 INFORMED CONSENT PROCEDURES AND DOCUMENTATION

Informed consent is a continuing process that is initiated prior to the individual's agreeing to participate in the study and continues throughout the individual's study participation. Apart from this, parental/LAR consent form will also be completed in the age group younger than 18 years old group, agreeing to participate in the study and continues throughout the individual's study participation. Additionally, in accordance with the local regulation, the participant group aged 7-17 years, will also complete an assent process and form. Participants in the age group 18 to 40 years will be asked for consent through ICF only.

Information about the risks and possible benefits of participation will be provided to the participants/parent(s)/LAR through extensive discussion. Consent/Assent forms will be IRB/IEC-approved prior to their use and the participants/parent(s)/LAR will be asked to read the document for their better understanding about the study.

The investigator or designated study team member will explain the study to the participants/parent(s)/LAR and answer any questions that may arise. All participants will receive a verbal explanation in terms suited to their comprehension of the purposes, procedures, and potential risks of the study and of their rights as research participants. The participants/parent(s)/LAR will have the opportunity to carefully go through the written consent/assent form and ask questions prior to signing. Participant/parent(s)/LAR may wish to discuss the study with family or friends before making any decision as to whether or not to participate in the study and come back later to inform the site Investigator or designee of his/her decision. For those individuals who express interest in continuing with the consent/assent process, the site investigator or designee will review the consent/assent form privately in detail with the participant/parent(s)/LAR and answer any questions.

Before signing the consent/assent, participants/parent(s)/LAR will be asked to ensure that they fully understand the purpose of the study, procedures, potential risks and their rights in this study.

If the participant's representative is illiterate (*i.e.*, not able to read and sign the ICF/AF), then it must be signed and dated by an impartial witness who is independent of the Investigator after explanation. A witness who signs and dates the consent form is certifying that the information in this form and any other written information had been accurately explained to and understood by the participant's parent(s)/LAR. If minor participants visit the facility with LAR then valid identity document will be asked from them showing their legal guardianship and depending on their age and local regulations, the study participants will have to sign and date an AF.

The participants/parent(s)/LAR will sign the informed consent/assent documents prior to any procedures being done specifically for the study. The participants/parent(s)/LAR may withdraw consent/assent at any time throughout the course of the trial. A copy of the informed consent/assent document will be handed over to the participants/parent(s)/LAR for their records. The rights, safety, and wellbeing of the participants will be protected by emphasizing that the quality of their medical care will not be adversely affected even if they decline to participate in this study. The process should be documented in the medical record

7.2.3 COMPENSATION FOR PARTICIPATION

Compensation for time and inconvenience of study participation will be provided to participants in accordance with the site-specific IRB/EC approved study documents. This includes scheduled visits and unscheduled visits.

7.2.4 PREGNANCY PREVENTION COUNSELING ON FEMALE PARTICIPANTS OF CHILDBEARING POTENTIAL

Clinical staff must perform pregnancy prevention counseling on female participants of childbearing potential to evaluate if participants are able to meet the birth control eligibility criteria. The documentation of this procedure is explained in detail in the MOP. Additionally, female participants of childbearing potential will receive pregnancy prevention counseling throughout the vaccination period.

7.2.5 ELIGIBILITY CRITERIA

Inclusion Criteria

To be eligible to participate in this study, any individual must meet the following criteria:

- Healthy participants 1 to 40 years of age at enrollment
- Participants/Parent(s)/LAR willing to provide written informed consent to participate study voluntarily
- Participants/Parent(s)/LAR who can be followed up during the study period and can comply with the study requirements

Exclusion Criteria

An individual who meets any of the following criteria will be excluded from participation in this study:

- Known history of hypersensitivity reactions to other preventive vaccines
- Severe chronic diseases or medical conditions based on the medical judgment of the investigator. In particular, a participant with a) chronic infection such as tuberculosis, or sequel of poliomyelitis, b) known history of immune function disorders, c) chronic use of systemic steroids (>2 mg/kg/day or >20 mg/day prednisone equivalent for periods exceeding 10 days)/cytotoxic drugs/immunosuppressants within past 6 weeks, d) active malignancy with the exception of adequately treated basal cell or squamous cell skin cancer, in situ cervical cancer, adequately treated Stage I cancer from which the participant is currently in complete remission, or any other cancer from which the participant has been disease-free for 5 years, e) congestive heart failure, f) myocardial infarction within the previous 6 months, g) known HIV-infected patients, h) neurological and/or psychiatric disorder, or i) known history of uncontrolled coagulopathy or blood disorders
- Participant who received any other vaccines within 4 weeks prior to enrollment in OCV-S study or who plans to receive any vaccine within 4 weeks after the second dose of study vaccine administration
- Participant concomitantly enrolled or scheduled to be enrolled in another trial
- Receipt of blood or blood-derived products in the past 3 months
- Participant who has previously received a cholera vaccine
- Any female participant who is lactating, pregnant or planning for pregnancy during study period
- Participants planning to move from the study area before the end of study period
- Employee or the family members of the study center

Temporary Contraindication

Should a participant have one of the conditions/situations listed below, the Investigator will postpone primary or subsequent vaccination until the condition/situation is resolved.

- Febrile illness (axillary temperature ≥ 38°C) or moderate or severe acute illness/infection on the day of vaccination or planned vaccination, according to Investigator's judgment.
- Gastrointestinal symptoms including nausea, vomiting, or decreased appetite within 24 hours prior to trial vaccination.
- Administration of antidiarrheal drugs or antibiotics to treat diarrhea or abdominal pain lasting 2 weeks or longer within 6 months prior to trial vaccination
- Diarrhea occurring up to 1 week within 6 months prior to trial vaccination.
- Receipt of any vaccine in 4 weeks preceding the trial vaccination.

*Lactation: Breastfeeding women will not be enrolled. Should a female participant decide to breastfeed during the vaccination period, she will be excluded from further vaccination, but will be followed for safety until the end of the study.

** Pregnancy Test is necessary for all married female participants of childbearing age. If un-married female participants of childbearing age agree to take pregnancy test, the test should be performed.

7.2.6 MEDICAL HISTORY

A medical history of last 1 year will be obtained by the Investigator or qualified designee at the time of enrolment. Participants included will be assessed for pre-existing conditions and illnesses, both past and ongoing, as well as surgical procedures, that are considered to be clinically significant by the Investigator. Any such conditions will be documented in the source document and the medical history module of the electronic case report form (eCRF).

For each condition, the data collected will be:

- Diagnosis (this is preferable to reporting signs and symptoms).
- Presence or absence of the condition at enrollment.

7.2.7 CONTRAINDICATIONS FOR PRIMARY AND SUBSEQUENT VACCINATIONS

7.2.7.1 TEMPORARY CONTRAINDICATION

Should a participant experience one of the conditions/situations listed below, the Investigator will postpone further vaccination until the condition/situation is resolved.

- Febrile illness (axillary temperature ≥ 38°C) or moderate or severe acute illness/infection on the day of vaccination, according to Investigator judgment.
- Receipt of any other vaccine in the 4 weeks preceding the trial vaccination.

7.2.7.2 DEFINITIVE CONTRAINDICATION

The following conditions/situations listed below, are definitive contraindications and the site investigator must discontinue participants from vaccination:

1) Pregnancy, as indicated by a positive urine test.
2) An anaphylactic or other significant allergic reaction to the previous dose of vaccine.
3) Clinically significant AE or biological abnormality related to previous vaccination and, in the Investigator's opinion, contraindicating further vaccination.
4) SAE related to the study vaccine following the previous trial vaccination.

7.2.8 PARTICIPANT DISCONTINUATION/WITHDRAWAL FROM THE STUDY

Participants/Parents/LAR are free to withdraw (their child) from participation in the study at any time upon request, without justification and without prejudice. The Principal Investigator may also decide to discontinue participation of participant from study interventions in the following cases:

1) An acute reaction (allergy, hypersensitivity reaction, etc.) to the investigational product.
2) Occurrence of an illness or serious adverse event or adverse event that in the judgment of the investigator may be detrimental for the participant's safety.
3) A study participants/Parents/LAR withdrawal of informed consent (dropout). The reason for a withdrawal or dropout should be clearly documented in the source documents and on the eCRF.
4) A study participant's medical condition or use of medication that in the judgment of the investigator may compromise the participant's safety and/or the scientific integrity of the study.
5) Violation of the inclusion/exclusion criteria by the participant.
6) Significant non-compliance with the protocol, based on the Investigator's judgment and lost to follow-up.

7) Any other reason of study discontinuation as per the judgment of the Principal Investigator.

Withdrawn participants will not be replaced. Any unsolicited AE, SAE and concomitant medications will be recorded in CRF.

7.2.9 HANDLING OF PARTICIPANT DISCONTINUATION OR TERMINATION

Discontinuation from study intervention does not mean discontinuation from the study, and remaining study procedures will be completed as indicated by the study protocol. If a clinically significant finding is identified after enrollment, the PI will determine if any change in participant management is needed. Any new clinically relevant finding will be reported as an adverse event.

Study team will encourage participants/Parents/LAR of withdrawn or terminated participant to continue in the study for safety follow-up. If s/he declines, this will end the participant's interaction with the study team for this protocol. The study team will engage in no further communication with the volunteer except as directed by an IRB/IEC with regards to participant safety information. Protocol-specified safety follow-up procedures will be discussed with the participants/Parents/LAR to capture AEs, serious adverse events (SAEs), and unanticipated problems (UPs). The reason for participant discontinuation or withdrawal from the study will be recorded on the study termination form of CRF. Only data and samples already collected will be analyzed according to protocol. Counseling about any issue will be provided if he/she decides to discontinue participation in the study. Medical advice will also be provided in the best interest of the participant.

Participants who receive the study intervention and subsequently withdraw or are withdrawn or discontinued from the study will not be replaced.

In the event of early termination of a participant:

1) Date and Reason for early termination of the participant will be recorded in the eCRF.
2) Any unsolicited AE, SAE and concomitant medications up to that points will be recorded in eCRF.

7.2.10 LOST TO FOLLOW-UP

A participant will be considered lost to follow up if s/he fails to return for any of scheduled visits and remains unreachable to study site staff.

The following actions will be taken if a participant fails to return to the clinic for a required study visit:

- The site staff will attempt to contact the participants/Parents/LAR and counsel on the importance of maintaining the assigned visit schedule and ascertain if the participants/Parents/LAR wishes to and/or should continue in the study.
- Before a participant is deemed lost to follow-up, the investigator or designee will make every effort to regain contact with the participant/parent/LAR. The several contact attempts should be documented in the participant's medical record or study file. In the case of participants who fail to return for a follow-up examination, documented reasonable effort (*i.e.*, documented telephone calls and certified mail) should be undertaken to locate or recall them, or at least to determine their health status while fully respecting their rights. These efforts should be documented in the source document (*i.e.*, medical record).

Should the participant continue to be unreachable, s/he will be considered to have withdrawn from the study with a primary reason of lost to follow-up.

7.2.11 DISCONTINUATION FROM VACCINATION PHASE IN CASE OF PREGNANCY

Pregnancy is an exclusion criterion for enrolment in this study, but a participant could potentially become pregnant during her participation. All pregnancy cases should be reported as described in the section 7.2.14. Follow-up and reporting of Pregnancy. If a female participant who has already received at least one vaccination becomes pregnant during the trial, she will continue to be followed for safety assessments that the vaccine aims to prevent, and she will not be discontinued from the trial; however, no additional vaccination will be administered.
Study staff must then maintain contact with the participant to obtain information about the outcome of the pregnancy that begins during this study following the procedures of section 7.2.14. Follow-up and reporting of Pregnancy.

7.2.12 CLASSIFICATION OF PARTICIPANTS WHO DID NOT COMPLETE THE TRIAL OR THE VACCINATION PHASE

Participants who receive at least one product administration are expected to continue with planned follow-up visits until the end of the study. However, for any participant who discontinues the trial prior to completion, the reason for early termination will be noted in the eCRF, classified as one of the following:

- **Serious adverse event**: To be used when a participant drops out of or is withdrawn from the study by the Investigator because of the occurrence of an SAE.
- **Other adverse event:** To be used when a participant drops out of or is withdrawn from the study by the Investigator because of the occurrence of an AE other than an SAE.
- **Non-compliance with protocol:** To be used when the Investigator withdraws a participant from the study because of failure to follow protocol guidelines. This termination category may also be used if it is retrospectively discovered that a participant did not fulfill the eligibility criteria. The Investigator will provide a comment as to the specific cause of non-compliance.
- **Lost to follow-up**: To be used when the Investigator withdraws a participant from the study because of failure to establish contact. The Investigator will provide documentation that contact was attempted.
- **Voluntary withdrawal not due to an AE:** To be used when a participant drops out of the study for any reason other than those listed above.

7.2.13 FOLLOW-UP OF PARTICIPANTS WHO DID NOT COMPLETE THE TRIAL OR THE VACCINATION PHASE

For participants where the reason for early termination is voluntary withdrawal, the site will attempt to contact them to obtain further safety information and to ask them whether they accept to participate to the follow-up procedures.

7.2.14 FOLLOW-UP AND REPORTING OF PREGNANCIES

All female participants of childbearing potential will receive pregnancy prevention counseling throughout the vaccination period. Even though pregnancy is an exclusion criterion for enrollment in this study, a participant could potentially become pregnant during her participation. For this reason, women will be asked to inform the site immediately if they suspect or learn they are pregnant during the study.

In case of pregnancy, participants will be discontinued from the product administration schedule. However, she will not be discontinued from the trial and can continue to be followed up until the end of the study for safety assessments and can have blood draws at the discretion of the site PI and the sponsor.

All pregnancy cases should be reported if they occurred during this study. To report the pregnancy case, the investigator must fill out a Pregnancy Reporting Form within 1 month or depending on the NHRC, IRB/IEC pregnancy report guidelines, after identifying a pregnancy case. Study staff must then maintain contact with the participant to obtain information about the outcome of the pregnancy that begins during this study (*i.e.*, details about the delivery and the newborn, or about pregnancy termination) and must update the Pregnancy Reporting Form (or the corresponding paper version if the electronic version is not available). This information should be provided to the Sponsor within one month of the end of the pregnancy.

Pregnancy itself is not considered an AE, but any complications during pregnancy are to be considered as AEs, and in some cases could be considered SAEs. Spontaneous abortions, fetal death, stillbirth and congenital anomalies reported in the baby are always considered as SAEs, and the information should be provided using an SAE form.

Women will be consulted to practice effective contraception from at least 4 weeks prior to first vaccination until at least 4 weeks after the last vaccination.

7.2.15 PROTOCOL DEVIATIONS

The ICH E3 Q&A R1 defines a protocol deviation (PD) as “any change, divergence, or departure from the study design or procedures defined in the protocol.” In other words, protocol deviation is any noncompliance with the clinical trial protocol, ICH E6 (R2) GCP, E11 (R1) or MOP requirements by the participant, the investigator, or the study site staff. It is the responsibility of the site PI to use continuous vigilance to identify and report all protocol deviations to the site IRB/IEC and to the sponsor. The PI is responsible for knowing and adhering to the site requirements. The IVI Study Medical Monitor will report all protocol deviations to IVI IRB/IEC and only major PDs have to be reported to IVI IRB/IEC within 10 calendar days of their awareness.

The ICH guidelines also introduce a definition for “important” protocol deviations, defining them as “a subset of protocol deviations that may significantly impact the completeness, accuracy,

and/or reliability of the study data or that may significantly affect a participant's rights, safety, or well-being." Important, "major", "critical" and "significant" are synonyms when referring to important protocol deviations.

To align the sponsor terminology with that of the health authorities' (*i.e.*, Regulatory Agency inspectors), we are using the terminology major and minor instead of the classification of important and non-important protocol deviations.
Major deviations are defined as those jeopardize the safety or rights of the participant or the scientific integrity of the study which is applicable to cases listed below.

- Violation of inclusion and exclusion criteria
- Vaccination with wrong vaccine as defined in the protocol
- Vaccination outside window of the immunization schedule defined in protocol
- Missed visit or visit outside window for the immunogenicity assessment

Major protocol deviations thought to affect the scientific integrity of the study and/or the safety and rights of the participant will be reported and discussed with investigator, monitor, sponsor, and statistician for their exclusion from the per protocol analysis.

For minor protocol deviations considered not to affect the scientific integrity of the study, the extent of deviation or delay as well as reason will be accurately documented. The equivalent definition to minor protocol deviation would be, a non-important protocol deviation. However, there is no formal definition of a non-important protocol deviation in ICH. It follows that if a protocol deviation does not meet the criteria of important, it is non-important.

Protocol deviation documentation must include a description of the deviation (including at which visit the deviation occurred, if applicable), the cause, and the plan to correct and prevent such deviations from occurring in the future. The root cause identification analysis and the corrective and preventive actions are to be implemented promptly.

7.3 PROTOCOL AMENDMENT

Any protocol amendment must be made only with the prior approval of the Sponsor and the amended version of the protocol will replace the earlier version.

Agreement from the investigator must be obtained for all protocol amendments and amendments to the informed consent document. All amendments require IRB/IEC approval, and those amendments that affect the conduct of the trial or the safety of participants must also be forwarded to the NHRC for approval before implementation, according to local requirements.

An administrative amendment to a protocol is one that modifies some administrative or logistical aspect of the trial but does not affect its design or objectives or have an impact on the participants' safety. Some Regulatory authorities need only be notified about administrative changes.

The Investigator is responsible for ensuring that changes to an approved trial, during the period for which IRB/IEC approval has already been given, are not initiated without IRB/IEC review and approval.

7.4 PREMATURE TERMINATION OR SUSPENSION OF STUDY

This study may be temporarily suspended or prematurely terminated if there is sufficient reasonable cause. IVI and other regulatory authorities reserve the right to terminate the study. Each site PI will notify the respective site IRB/IEC of the study termination in writing and provide documentation to the sponsor. Written notification, documenting the reason for study suspension or termination, will be provided by the suspending or terminating party to investigator, the sponsor, the regulatory authorities, and IRB/IECs.

Circumstances that may warrant termination or suspension are:

- Determination of unexpected, significant, or unacceptable risk to participant as recommended by the PI
- Poor protocol compliance

Study may resume once concerns about safety, protocol compliance, data quality is addressed, resolved, and meeting the requirements from the sponsor, IRB/IECs, NHRC and/or DSMB/SMC.

7.5 END OF STUDY

A participant is considered to have completed the study if s/he has completed all study visits including the last visit or the last scheduled procedure shown in the Schedule of Events according to group allocation.

8. INVESTIGATIONAL PRODUCT AND CONTROL DESCRIPTION

8.1 IDENTITY OF THE INVESTIGATIONAL PRODUCT: OCV-S

- Manufacturer: EuBiologics Co., Ltd.
- Appearance: yellow to yellowish suspension
- The test article, oral cholera vaccine simplified, is available in 1.5 mL plastic tube as a single dose.
- Route: oral administration

8.1.1 COMPOSITION

- Component:
  - Formalin inactivated Vibrio cholerae O1 Inaba Phil 6973, El Tor and O1 Ogawa Cairo 50, Classical biotypes.
  - Buffer q.s.

8.1.2 PREPARATION OF ADMINISTRATION

OCV-S will be given to participants orally by skilled and delegate personnel at each site. 1.5 mL of the investigational vaccine will be administered. Refer to the MOP/IB for information on study products preparation and use.

Prior to administration, all study products must be inspected visually for cracks, broken seals, correct label content, and extraneous particulate matter and/or discoloration, whenever solution and container permit. If any of these conditions exists, the vaccine must not be administered. A replacement dose is to be used, and the event is to be reported to the Sponsor. According to the MOP and IB, labels may include the participant identifier, treatment number, time removed from freezer, time of expiration, and preparer's initials and date.

If a tube is accidentally broken and the product spilled out, appropriate disinfection procedures must be used (refer to the MOP and/or site center's Procedures). Site staff should practice universal precautions and dispose of tubes in keeping with the site policy and practices.

The trial staff/pharmacist administering the vaccine would have the tube covered with opaque material to ensure blinding of the participants as well Investigator(s) and clinical supervisors to treatment arm.
Site staff will be vigilant to identify severe allergic or anaphylactic reactions following vaccine administration. Appropriate medical equipment and emergency medications must be available on site in the event of an anaphylactic or other immediate allergic reaction.

8.2 IDENTITY OF CONTROL PRODUCT: SHANCHOL™

8.2.1 COMPOSITION

- Component:
    - Formaldehyde inactivated O1 Inaba Phil 6973 El Tor biotype (600 LEU), O1 Ogawa Cairo 50 Classical biotype (300 LEU) and O139 4260B biotype (600LEU), and heat inactivated O1 Ogawa Cairo 50 Classical biotype (300LEU) and O1 Inaba Cairo 48 Classical biotype (300LEU) per dose
    - Thiomersal B.P. less than 0.02%(w/v)
    - Buffer q.s.

8.2.2 PREPARATION OF ADMINISTRATION

Shanchol™ will be given to participants orally by skilled and delegate personnel at each site. 1.5 mL of the investigational vaccine will be administered. Refer to the MOP/IB for information on study products preparation and use.
Prior to administration, all study products must be inspected visually for cracks, broken seals, correct label content, and extraneous particulate matter and/or discoloration, whenever solution and container permit. If any of these conditions exists, the vaccine must not be administered. A replacement dose is to be used, and the event is to be reported to the Sponsor.
If a vial is accidentally broken and the product spilled out, appropriate disinfection procedures must be used (refer to the MOP and/or site center's Procedures). Site staff should practice universal precautions and dispose of vials in keeping with the site policy and practices.

The trial staff/pharmacist administering the vaccine would have the vial covered with opaque material to ensure blinding of the participants as well Investigator(s) and clinical supervisors to treatment arm.
Site staff will be vigilant to identify severe allergic or anaphylactic reactions following vaccine administration. Appropriate medical equipment and emergency medications must be available on site in the event of an anaphylactic or other immediate allergic reaction.

8.3 PRODUCT LOGISTICS

8.3.1 LABELLING AND PACKAGING

The vaccine package will consist of 1 tube/vial of OCV-S/Shanchol™.

At the time of study product delivery to the pharmacy, labels on an investigational product (OCV-S) will have the following product information:

- Study code
- Name of product / Drug code
- Randomization number
- Investigational use only statement
- Manufacturer information
- Storage conditions
- Batch number / Lot number
- Manufacturing date / Expiry date
- Route of administration

All the products will be identified for group assignment.

8.3.2 PRODUCT SHIPMENT, STORAGE, STABILITY AND ACCOUNTABILITY

IVI will ensure appropriate supply of study vaccinations at all sites. The OCV-S/Shanchol™ will be shipped to study sites in Nepal. The pharmacist or designee will receive the study products

and will be responsible for accounting, storage, handling and administration of the vaccines. The detail working process of study sites will be narrated in the Manual of Procedure (MOP).
The Logistics designee will contact the site principal investigator or a designee in order to determine the dates and times of delivery of products.

8.3.2.1 PRODUCT SHIPMENT

Vaccines must be kept in temperature-controlled environments at all times throughout the shipment process including in transit storage points/warehousing. Every vaccine shipment will include a temperature-monitoring device to verify maintenance of the cold chain during transit. On delivery of the product to the site, the delegated personnel in charge of product receipt must follow the instructions given in the MOP, including checking that the cold chain was maintained during shipment (*i.e.*, verification of the temperature recorders). If there is an indication that the cold chain was broken, the site designee personnel should immediately quarantine the product, alert the IVI, and request authorization from IVI to use the product.

8.3.2.2 PRODUCT STORAGE

Vaccine tubes/vials will be shipped to the study pharmacy at the recommended temperature range using appropriate shipping configurations. At the site, products must be kept in a secure place with restricted access Vaccines will be stored in a in a qualified, continuously monitored, temperature-controlled refrigerator at the target temperature of +2°C to +8°C. The vaccines must not be frozen.

The temperature must be monitored and documented for the entire time that the vaccine is at the trial site. In case of accidental freezing or disruption of the cold chain, vaccines must not be administered and must be quarantined, and the Investigator or authorized designee should contact the IVI representative for further instructions.

If deviations in storage temperature occur from the normal allowance for the pharmacy refrigerator, the site pharmacist or designee must quarantine affected product(s) and report the storage temperature excursion as soon as detected to the site PI and the IVI. The excursion must be evaluated and investigated, and action must be taken to restore and maintain the desired

temperature limits. Pending the outcome of the investigation, the Sponsor will notify the site pharmacist or designee if clinical use of the affected product is acceptable and the affected product may be removed from quarantine.

8.3.2.3 PRODUCT ACCOUNTABILITY

Documentation

Each study site will be responsible for maintaining an accurate record of the treatment codes, inventory and an accountability record of the investigational study product supplies for this study.

Disposition

•Empty tubes/vials and the unused portion of a tube/vial must be discarded at the end of the vaccination period that will be incinerated or autoclaved in accordance with site policy.

Any unopened tubes/vials that remain at the end of the study will be discarded at the discretion of the sponsor in accordance with policies or guidance from the sponsor that apply to investigational products.

All Used vaccines will be disposed at the site following reconciliation as per the Nepalese national regulation. All expired vaccines will be disposed as per the Nepalese national regulation.

8.3.3 PRODUCT PREPARATION

The preparation and administration of the vaccines to participants enrolled into the study will only be done by the unblinded study personnel according to the procedures stipulated in this study protocol. The study personnel responsible for vaccine administration is qualified to perform this task and same will be documented by site investigator.

The Shanchol^{TM} will be prepared before use, according to the package insert. For further details please refer to the MOP.

8.3.4 REPLACEMENT DOSES

In case a replacement dose is required (e.g., because the tube/vial broke or particulate matter was observed in the tube/vial), the site personnel must follow the instructions given in the MOP and contact the sponsor to get to obtain a new assigned product.

8.3.5 DISPOSAL OF UNUSED PRODUCTS

Unused or wasted products will be either disposed of in the site or returned to the Sponsor in accordance with the instructions in the MOP.

8.3.6 RECALL OF PRODUCTS

In case the Sponsor decides to launch a retrieval procedure, the Investigators will be informed of what needs to be done.

8.4 RANDOMIZATION AND ALLOCATION PROCEDURES

Before randomization, pregnancy test results must be confirmed as negative.
Each participant who meets the inclusion/exclusion criteria and signs an ICF/AF will be randomly assigned to the respective groups.
The randomization list will be generated by an independent statistician who is not directly involved in the study conduct. The randomization will be stratified by three age strata. Eligible participants will be assigned to receive OCV-S or Shanchol™. The randomization list will contain sequential numbers unique to each participant and the block randomization process will be employed to ensure an effective balance between the interventions. Only the independent statistician will have a complete set of randomization lists. The randomization list will be uploaded into Randomization and Trial Supply Management system (RTSM) to be used by the pharmacy staff or designee at all clinical sites. After the completion of the database lock, unblinding of the assignment to the respective vaccine groups will be carried out for statistical analysis and the lists will be returned to the statistician.

Trial staff other than the unblinded study staff will remain blinded to vaccine administration. The unblinded study nurse/pharmacist will not be involved in the evaluation of vaccine safety and will not discuss with the investigator and clinical staff about vaccines administered.

8.5 BLINDING

The PI, study staff, and participants will be blinded as to receipt of study vaccine or comparator. The pharmacy staff preparing the vaccine and the study nurse who is administering the vaccine will not be involved in the safety assessment of participants and will be instructed not to comment on the experimental agent to study staff. The site pharmacy staffs, and the study nurse must not discuss randomization codes or participant assignments.

The enrollment/randomization number with its treatment assignment is generated in advance by the independent study statistician. The randomization information is only available to the study nurse/pharmacist and independent statistician. Enrollment/Randomization numbers will be assigned sequentially by RTSM upon confirmation of eligibility and enrollment into the study according to their age strata by the Investigator. All source documents will be labeled with the randomization number and study visit number. Personal identifying information linking the study number to an individual volunteer will not be captured on eCRF as study data. This linkage if site requires will be maintained electronically in a secured, password protected computer on site for the purpose of volunteer scheduling and verification of identity.

8.6 UNBLINDING OF PARTICIPANTS

A request for unblinding, with its rationale, must be forwarded through the PI. The PI will evaluate the request and will notify the Study Medical Monitor (SMM). The SMM will evaluate the request and will advise the Sponsor regarding a course of action. The Sponsor will decide whether to approve the request for unblinding. In the case of the former, the Sponsor will authorize the independent statistician to provide this information to the PI. It should be noted that there are very few circumstances in which unblinding will be essential to the medical management of a vaccine (or comparator) recipient. In case of vaccine-related death or life threatening, SAEs, knowledge of whether a participant received vaccine or comparator can be critical for the interpretation of the significance of clinical findings and thus impact decisions regarding continuation of study participation. In such cases, the assignment of a participant may be unblinded.

Episodes of unblinding, whether accidental or intentional, will be reported by the site investigator (either by email or fax) with an explanation to the sponsor at IVI who will in its turn inform IVI IRB/IECs. Apart from this, the Site PI is also responsible for informing their own IRB/IEC. Other participating IRB/IECs will be informed through Sponsor. Follow-up of such participants will continue throughout the duration of the trial.

Study participants will be unblinded after the completion of the database lock. See section 13.3 Statistical Analysis Plan. The database will be locked for the statistical analysis after safety and immunogenicity data is cleaned and locked last participant's last follow-up visit.

8.7 TREATMENT COMPLIANCE

In order to ensure that the vaccine doses administered comply with those planned, and that any non-compliance is documented so that it can be accounted for in the data analyses, the following measures must be applied:

- All vaccinations will be administered by qualified trial personnel.
- The person in charge of product management at the site will maintain accountability records of product delivery to the trial site, product inventory at the site, dose(s) given to each participant, and the disposal of unused or wasted doses.

8.8 CONCOMITANT MEDICATION

A prescription medication is defined as a medication that can be prescribed only by an authorized/licensed physician. Medications to be reported in the Electronic Case Report form (eCRF) are concomitant prescription medications, over-the-counter medications and supplements.

Only routine medications should be entered in the database at the time of study randomization.

All approved vaccinations for routine health care must be entered in the database throughout the study.

Concomitant medications will be updated in the study database if there is an occurrence of an AE that requires expedited reporting or development of a new chronic medical condition that requires ongoing medical management. Otherwise, concomitant medications taken during the study must be recorded in the participant study file.

Vaccines during the Study: Participants may receive routine vaccinations throughout the study as needed for standard healthcare practice. Product administrations must be scheduled such that:

- No inactivated vaccine is received within 4 weeks before or after each product administration.
- No live attenuated vaccine is received within 4 weeks before or after each product administration.

9. LABORATORY PROCEDURES/EVALUATIONS

9.1 LABORATORY EVALUATIONS

Blood samples will be obtained from participants for immunogenicity and lot-to-lot consistency assessments. For immunogenicity evaluation, vibriocidal assays against *Vibrio cholerae* O1 Inaba and Ogawa 2 weeks after the second dose of OCV-S to Shanchol™ will be performed. Laboratory test results are dependent on the quality of the specimen submitted. It is important that all specimens and test requisition forms are properly labelled in the presence of the participant.

- Blood sample will be collected in appropriate standard vacutainer tubes for the specific analysis as per the details provided in the MOP.
- Samples will be labelled with participant ID, date, visit number, and etc. The blood will be processed and aliquoted according to the MOP.
- All aliquots will be frozen and stored at temperatures mentioned in the MOP, before being shipped to the IVI laboratory. Temperature monitoring with backup generators, will be in place to ensure proper sample storage.
- The details of the blood collection, fractionation, storage and shipment will be provided in the MOP.

9.2 SPECIMEN PROCESSING, HANDLING, AND STORAGE

Venous blood will be collected from participants. For immunogenicity assessments whole blood will be centrifuged, and sera will be aliquoted and stored at temperature of below -20°C in a qualified, continuously monitored, temperature-controlled freezer until shipment to IVI and storage for future use. Pre-print study labels provided in advance will be attached on each of the serum aliquots.

Serum Sample Preparation

Following the blood draw, the sampling tube should be stored at room temperature for a minimum of 60 minutes and a maximum of 2 hours to allow the blood to clot before centrifugation. The tube must be stored vertically and will not be shaken. Beyond 2 hours, the sampling tube must be

refrigerated at a temperature of +2°C to +8°C after the period of clotting at room temperature and must be centrifuged within a maximum of 24 hours.

After clotting and centrifugation, the serum will be harvested and divided into two aliquots. Samples will then be handled one participant at a time to avoid a mix-up of participants' blood tubes. Serum will be transferred to the appropriate number of tubes, pre-labeled with adhesive labels that clearly identify the participant's number and sampling stage or visit number. The participant's identification number and code, the date of sampling, the number of aliquots obtained, and the date and time of preparation are to be specified on a sample identification list. Serum will be aliquoted in tubes which are specified in the MOP. Aliquots will be frozen immediately at the target temperature of -20°C in a qualified, continuously monitored, temperature-controlled freezer until testing.

9.3 SPECIMEN SHIPMENT

Shipments to the laboratories will be made only after appropriate monitoring and following notification of the Logistics Coordinator. Sera will be shipped frozen, using dry ice to maintain them in a frozen state, in the packaging container provided by the carrier. Temperatures will be monitored. Shipments must be compliant with the IATA (International Air Transport Association) regulations.

Aliquoted serum samples for immunogenicity assessment will be shipped from Nepal clinical trial sites to the IVI laboratory, Korea, where they will be stored below -20°C for analysis and storage for 10 years after completion of CSR or according to participant's decision about preservation period.

9.4 ASSESSMENT OF IMMUNOGENICITY

Blood sample will be collected for immunogenicity assessments at time-points specified in the Table 2. Study Procedures/Schedule of Events.

Table 5. Blood Sample Volume by Age

Age of participants	**1-5 Years**	**6-40 Years**
Blood sample volume	Approximately 4 mL	Approximately 6 mL

*Minimum volume for 1-5 years old is 3 mL.

10. ASSESSMENT OF SAFETY

10.1 SAFETY ASSESSMENT

The following procedures will be performed to monitor safety as listed in the SOE:

- **Demographic and medical history** (date of birth, age, gender, baseline medical/medication history of participants)
- **Physical examination** (height and weight, organ systems, growth and development and motor assessments for age eligible participants)
- **Vital signs** (body temperature, pulse, respirations, blood pressure)
- **Diary cards** will be used for reported outcomes

At each study visit, the investigator will inquire about the occurrence of AE/SAEs since the last visit.

10.1.1 DEFINATION OF ADVERSE EVENTS

Adverse events (AE): These are defined as any untoward medical occurrence which follows vaccination, and which does not necessarily have a causal relationship with the administration of the vaccine. An AE may be any unfavorable or unintended sign, symptom, or disease.

Adverse Drug Reactions (ADR): All noxious and unintended responses to a medicinal product related to any dose should be considered adverse reactions (AR). The phrase "responses to a medicinal product" means that a causal relationship between a medicinal product and an AE is exist with at least a reasonable possibility.

Solicited AEs are predetermined events, identified in the Investigator's Brochure (IB), which may reflect safety concerns related to the investigational product. AEs that will be solicited by the participant/ parents/LAR and recorded in the diary card and reviewed by a blinded observer during the 7 days after each dose for this study include fever, nausea/vomiting, diarrhea, headache, fatigue, myalgia and anorexia/loss of appetite.

Unsolicited AEs are all other adverse events (those that do not fall under the categories of Solicited AEs listed above) that are identified by site staff, the site investigator and the Safety Medical Monitors. These unsolicited AEs will be documented in the participant's study records and entered in the study eCRFs.

Results will be expressed as frequency of the AEs and individual descriptions will be tabulated according to MedDRA organ class system and preferred term.

10.1.2 DEFINITION OF SERIOUS ADVERSE EVENTS

An AE or suspected adverse reaction is considered "serious" if, in the view of either the investigator or sponsor, it results in any of the following outcomes:

- Results in death
- Life-threatening event [1]
- Requires in-patient hospitalization or prolongation of existing hospitalization [2]
- Results in persistent or significant incapacity [3]
- Is a congenital anomaly/birth defect; [4] or
- Is an important medical event that may jeopardize the participant or may require intervention
- to prevent one of the other outcomes listed above

1. The term “life-threatening” refers to an event in which the participant was at risk of death at the time of the event; it does not refer to an event which hypothetically might have caused death if it were more severe.
2. All medical events leading to hospitalizations will be recorded and reported as SAEs, with the exception of hospitalization planned before inclusion into the study or out-patient treatment with no hospitalization.
3. “Persistent or significant disability or incapacity” means that there is a substantial disruption of a person’s ability to carry out normal life functions.
4. Characteristic or abnormality existing at birth and found in the participant's offspring and not in the participant his/herself

Serious and severe are not synonymous. The term severe is often used to describe the intensity of a specific event as corresponding to Grade 3. This is not the same as serious which is based on patient/event outcome or action criteria usually associated with events that pose a threat to a patient’s life or functioning. Seriousness, not severity, serves as a guide for defining regulatory reporting obligations.

10.1.3 DEFINITION OF SUSPECTED UNEXPECTED SERIOUS ADVERSE EVENT

- Suspected adverse reaction means any AE for which there is a reasonable possibility that the

drug caused the AE.

- Unexpected adverse event means an AE that is not listed in the IB or is not listed at the specificity or severity that has been observed.

Suspected unexpected serious adverse event (SUSAR) is defined as a serious adverse reaction whose nature or severity is not consistent with the applicable product information, Investigator's Brochure (IB) or the summary of product characteristics of an authorized product.

10.1.4 DEFINITION OF UNANTICIPATED PROBLEMS

Non-serious Unanticipated problems (UP): An UP that is not an Adverse Event (UP non AE) is an unanticipated problem that does not fit the definition of an AE, but which may, in the opinion of the investigator, involve risk to the participant, affect others in the research study, or significantly impact the integrity of research data. Such events would be considered a non-serious UP. For example, accidental destruction of study records or samples.

Serious Unanticipated problems include any incident, experience, or outcome that meets all of the following criteria:

- Unexpected in terms of nature, severity, or frequency of what is stated under adverse events in the protocol informed consent and Investigator's Brochure.
- Related or possibly related to vaccination
- Suggests that participants or others will be at a greater risk of harm (including physical, psychological, economic, or social harm) than was previously known or recognized.

10.2 CLASSIFICATION OF AN ADVERSE EVENT

10.2.1 SEVERITY OF EVENT

Grading the Severity of Adverse Events: The FDA Guidance for Industry (September 2007): "Toxicity Grading Scale for Healthy Adult and Adolescent Volunteers [Participants] Enrolled in Preventive Vaccine Clinical Trials"[19] is the basis for the severity grading for most of the AEs in this protocol.

See Table 8. Grading scale of AE: Clinical Abnormalities.

Grading the Severity of Adverse Events.

The following guidance will be used to assign a severity grade:

Grade 1 (Mild): No effect on activities of daily living

Grade 2 (Moderate): Some interference with activity not requiring medical intervention

Grade 3 (Severe): Prevents daily activity and requires medical intervention

Grade 4 (Potentially Life-threatening): Hospitalization; immediate medical intervention or therapy required to prevent death.

Grade 5 (Death): Death is assigned a Grade 5 severity.

Only the single AE that is assessed as the primary cause of death should be assigned "Grade 5" severity.

10.2.2 RELATIONSHIP TO INVESTIGATIONAL PRODUCT

For all collected AEs, the investigator who examines and evaluates the participant will determine the relationship of each AE with the investigational product based on plausible biologic mechanism, temporal relationship of occurrence after administration of the investigational product, identification of possible alternative etiologies including underlying disease, concurrent illness or concomitant medication, and the investigator's clinical judgment. The relationship of vaccination to adverse event (AE) will be determined based on the definitions below.

Definitely Related – There is clear evidence to suggest a causal relationship, and other possible contributing factors can be ruled out. The clinical event, including an abnormal laboratory test result, occurs in a plausible time relationship to vaccine administration and cannot be explained by concurrent disease or other drugs or chemicals. The response to withdrawal of the Vaccine (dechallenge) should be clinically plausible.

Probably Related – There is evidence to suggest a causal relationship, and the influence of other factors is unlikely. The clinical event, including an abnormal laboratory test result, occurs within a reasonable time after administration of vaccine, is unlikely to be attributed to concurrent disease or other drugs or chemicals, and follows a clinically reasonable response on withdrawal (dechallenge).

Possibly Related – There is some evidence to suggest a causal relationship (e.g., the event occurred within a reasonable time after administration of vaccine). However, other factors may have contributed to the event (e.g., the participant clinical condition, other concomitant events). Although an AE may rate only as “possibly related” soon after discovery, it can be flagged as requiring more information and later be upgraded to “probably related” or “definitely related,” as appropriate.

Unlikely to be related – A clinical event, including an abnormal laboratory test result, whose temporal relationship to vaccine administration makes a causal relationship improbable (e.g., the event did not occur within a reasonable time after administration of vaccine) and in which other drugs or chemicals or underlying disease provides plausible explanations (e.g., the participant clinical condition, other concomitant treatments).

Not Related – The AE is completely independent of vaccine administration, and/or evidence exists that the event is definitely related to another etiology. There must be an alternative, definitive etiology documented by the clinician.

The criteria for determining causality can be documented as follows: The initial four definitions can be considered as ADR and the last one *i.e.* “Not Related” can be considered as Non-ADR.

10.2.3 EXPECTEDNESS

The Study Medical Monitor in consultation with site PI will be responsible for determining whether an AE is expected or unexpected. An Adverse Reaction will be considered unexpected if the nature, severity, or frequency of the event is not consistent with the risk information previously described for the study agent.

10.3 TIME PERIOD AND FREQUENCY FOR EVENT ASSESSMENT AND FOLLOW-UP

All participants will be observed for immediate adverse reactions for 30 minutes after each vaccination. For 7 consecutive days (Days 0-6) after each dose of study vaccine, the participant/parent(s)/LAR will be asked to record solicited symptoms in the diary card. The study staff will remind participant/parent(s)/LAR of the importance of properly filling the diary cards and to return the cards at the next scheduled study visit. If they did not fill up or lost their card, the

participant/parent(s)/LAR will be interviewed for recall of symptoms with trained study staff during clinic visit on Day 7 after vaccination.

The occurrence of an adverse event (AE) will come to the attention of study personnel during study visits and interviews of a study participant presenting for medical care, or upon review by a study monitor.

All AEs not meeting the criteria for SAEs will be captured on the appropriate case report form (eCRF). Information to be collected includes event description, time of onset, symptoms and physical examination findings, clinician's assessment of severity, relationship to study product (as assessed by the PI or delegated SI), medications given and time of resolution/stabilization of the event. All AEs occurring while on study will be documented appropriately regardless of relationship.

Changes in the severity of an AE will be documented to allow an assessment of the duration of the event at each level of severity to be performed. AEs characterized as intermittent require documentation of onset and duration of each episode.

The investigator will record all reportable events with start dates occurring any time after informed consent is obtained until the last day of study participation. At each study visit, the investigator will inquire about the occurrence of AE/SAEs since the last visit. Events will be followed for outcome information until resolution or stabilization.

10.4 REPORTING PROCEDURES

10.4.1 ADVERSE EVENT RECORDING AND REPORTING

Adverse events, solicited AEs, and SAEs will be assessed at various study visits, documented in the source record, and recorded in the eCRF using accepted medical terms and/or the diagnosis that accurately characterize the event. When the diagnosis is known the AE term recorded in the eCRF will be the diagnosis rather than constellation of symptoms. The site PI or delegated SI will assess all AEs for seriousness, relationship to investigational product, severity, and other possible causes.

The timeframe for the collection of adverse events (AEs) occurring from the first administration of investigational product through to the end of the trial and will be collected as well as recorded in the source document and eCRF according to the MOP.

All AEs will be followed until they have resolved or are considered stable during the study. When an AE has not resolved by the current visit it will be documented in the eCRF as ongoing. If the event has a resolution date, this date must be recorded on the Adverse Event form, regardless of whether it falls within the AE reporting period.

Documentation will include date of onset, detailed description of the event and relevant history and physical examination, severity, attribution of the AE, treatment given and date the AE improved or resolved. The medical monitor will review the AEs reported regularly and clarify with PI or SI if there are queries. The data manager will review all AEs for consistency and provide summary of AEs to the medical monitor periodically. Non-clinically significant AEs still ongoing as the end of the study will be listed as continuing. SAEs continuing at the end of the study will be followed to resolution or stabilization. Details of AE reporting are included in the MOP.
The PI, sub-investigators, and site staff will exercise due diligence in ascertaining, accurately recording and promptly entering data on the eCRF for all AEs of all study participants. As data becomes available from the participant, the clinic adverse events should be recorded and entered by the site staff on regular basis. Site investigators will review, in a timely manner, the AE source data and determine the severity of the event and relation to the study agent. Site investigators will contact the study medical monitor for consultation of AEs as required.

10.4.2 SERIOUS ADVERSE EVENT REPORTING

The PI/SI will complete a SAE Form within the following time frame:

- All SAEs will be recorded on the SAE Form and submitted by the site PI to the Overall study PI/Sponsor within 24 hours of initial receipt of the information (weekends and holidays are not included) and addressed to:
- Dr. Katerina Rok Song
  Study Medical Monitor
  International Vaccine Institute
  SNU Research Park, 1 Gwanak-ro, Gwanak-gu, Seoul, 08826 Republic of Korea
  Phone: +82-2-881-XXXX (Dir)
  Fax: +82-2-881-1164
  E-mail: *Katerina.song@ivi.int*

- NHRC to be notified within 2 calendar days by the sponsor/overall study PI/Designee with complete report due within 21 additional calendar days of the first information.

All information (which may include special investigations and treatment received) will be recorded on the SAE Form and submitted to the NRA and site-specific IRB/IEC. All SAEs will be followed until satisfactory resolution or until the site investigator deems the event to be chronic or to be stable. Other supporting documentation of the event may be requested by the sponsor and should be provided as soon as possible. SAE reporting to IVI IRB on regular basis is not mandatory (except those are of SUSAR category) and will be reported with the annual renewal report.

- The sponsor will be responsible for notifying the IVI IRB of Suspected Unexpected Serious Adverse Reactions (SUSAR) within 24 hours of initial receipt of the information (weekends and holidays are not included)

The SUSAR report will include the following information:

- Protocol information: protocol number and date
- A detailed description of the event, incident, experience, or outcome
- An explanation of the basis for determining that the event, incident, experience, or outcome represents an unexpected problem

10.4.3 SAFETY OVERSIGHT

An internal **Safety Monitoring Committee (SMC)** will be responsible to oversee the vaccine safety patterns during the clinical trial. The SMC will be composed of individuals with appropriate expertise. The SMC will review blinded safety data on a regular basis according to the guidelines of the SMC charter. The SMC will send a summary of safety review findings to the site PIs, DSMB members, and the Study Medical Monitor.

An independent **Data Safety Monitoring Board (DSMB)** will be constituted of experts from various fields of Medicine external to sponsor's organization. The DSMB will oversee the study in terms of safety data as per DSMB charter. DSMB chair will issue a recommendation letter after each meeting. The DSMB will provide independent recommendations to the Sponsor and subsequently to Principal Investigator. The decision on whether to resume the study will be taken by the Sponsor and the Principal Investigator, based upon the recommendations of the DSMB.

The decision taken will then be communicated to the IRB/IEC and National Regulatory Agencies along with the DSMB recommendation by the principal investigators.

11. STUDY MONITORING

Before the inclusion of the first participant in the center, the Investigators and the Sponsor's monitoring staff, or a representative will meet at the site-initiation visit to discuss the trial protocol and the detailed trial procedures. Emphasis will be placed on inclusion and exclusion criteria, visit timing, safety procedures, informed consent procedures, SAE reporting procedures, eCRF completion, and the handling of samples and products.

Study monitoring and auditing will be performed in accordance with the sponsor's procedures, ICH E6 (R2) GCP guidelines and any other applicable regulatory requirements.

Upon successful approval of the protocol and establishment of the Regulatory File, the clinical monitor will establish a clinical monitoring plan (CMP). To ensure that the investigator and the study staff understand and accept their defined responsibilities, the clinical monitor will maintain regular correspondence with the site and may be present during the course of the study to verify the acceptability of the facilities, compliance with the investigational plan and relevant regulations, and the maintenance of complete records.

Investigators and/or their study staff will be trained on the study protocol and all applicable study procedures prior to study initiation. Electronic CRFs supplied by the sponsor must be completed for each enrolled participant. The data entries as well as study related documents will be checked by the sponsor and/or trained delegates of the sponsor.

Study progress will be monitored by IVI study team or representative (e.g., a clinical research organization) as frequently as necessary to ensure the rights and well-being of study participants are protected; to verify adequate, accurate and complete data collection; protocol compliance and to determine that the study is being conducted in conformance with applicable regulatory requirements. Arrangements for monitoring visits will be made in advance in accordance with the monitoring plan, except in case of emergency.

A report of monitoring observations will be provided to the principal investigator. All clinical and research records must be available for review by the sponsor's representative, local IRB

representatives, and other regulatory agencies as part of their responsibilities for insuring the protection of research participants.

At the end of the trial, a close-out visit will be performed to ensure that:

- The center has all the documents necessary for archiving
- All samples have been shipped to the appropriate laboratories
- All unused materials and products have been either destroyed or returned to the Sponsor

12. QUALITY ASSURANCE AND QUALITY CONTROL

Quality Assurance (QA) oversight will be required at all stages of the trial process per ICH E6 (R2) and/or local government GCP requirements.

Quality Control (QC) procedures will be implemented beginning with the data entry system and data QC checks. Any missing data or data anomalies will be communicated to the site(s) for clarification/resolution.

During study conduct, the sponsor or its designee (e.g., CRO) will conduct periodic monitoring visits (*i.e.*, QC checks) to ensure that the protocol, Good Clinical Practice, local regulatory requirements and sponsor's controlled documents (e.g., Standard Operating Procedure) are being followed. The monitors will review source documents to confirm that the data recorded on CRFs/eCRFs are accurate.

In addition to on-going QA oversight, selected investigator sites will be subjected to quality assurance audits performed by the sponsor or its designee, and/or by inspection by regulatory authorities and/or notified bodies.

The investigational sites will provide direct access to all study related sites, source data/documents, and reports for the purpose of monitoring and auditing by the sponsor; inspection by local and regulatory authorities and/or notified bodies.

13. STATISTICAL CONSIDERATIONS

13.1 STUDY ENDPOINTS

13.1.1 PRIMARY IMMUNOGENICITY ENDPOINT

- Proportion of participants showing seroconversion of vibriocidal titers against *Vibrio cholerae* O1 Inaba and O1 Ogawa (seroconversion is defined as at least 4-fold increase of vibriocidal titers against *V. cholerae* O1 Inaba and O1 Ogawa compared to baseline) at 2 weeks after second dose of either OCV-S (*i.e.*, one lot of OCV-S) or Shanchol™ for all ages.

13.1.2 PRIMARY SAFETY ENDPOINT

- Frequency of solicited adverse events within 7 days post vaccination, unsolicited adverse events within 28 days post vaccination, and SAEs after each dose during the entire study period in all ages and in each age stratum.

13.1.3 SECONDARY ENDPOINTS

- Geometric Mean Titer of vibriocidal antibodies against *Vibrio cholerae* O1 Inaba and Ogawa 2 weeks after second dose of either OCV-S (*i.e.*, one lot of OCV-S) or Shanchol™ for all ages
- Proportion of participants showing seroconversion against *Vibrio cholerae* O1 Inaba and Ogawa 2 weeks after second dose of either OCV-S (*i.e.*, one lot of OCV-S) or Shanchol™ in each age stratum
- Geometric Mean Titer of vibriocidal antibodies against *Vibrio cholerae* O1 Inaba and Ogawa 2 weeks after second dose of either OCV-S (*i.e.*, one lot of OCV-S) or Shanchol™ in each age stratum
- Geometric Mean Titer of vibriocidal antibodies against *Vibrio cholerae* O1 Inaba and Ogawa 2 weeks after second dose of 3 lots of OCV-S in adults

13.1.4 EXPLORATORY ENDPOINT

- Proportion of participants showing seroconversion of vibriocidal titers against *Vibrio cholerae* O1 Inaba and Ogawa 2 weeks after second dose of 3 lots of OCV-S in adults
- Seroconversion rate and GMT of vibriocidal antibodies 2 weeks after first dose of either OCV-S or Shanchol™ for all ages and for each age stratum

13.2 SAMPLE SIZE

The sample size of the study is calculated to provide about 90% power to show primary Immunogenicity objective of immune non-inferiority of OCV-S to Shanchol™ and secondary immunogenicity objective of immune equivalence of three lots of OCV-S of the study and increased to satisfy total safety population of about N=2,530. The given sample size of N=935 participants per OCV-S and Comparator vaccine group in all ages would provide 99% power to detect the non-inferiority of immunogenicity of OCV-S group (Group C) compared to Shanchol™ (Group D) in terms of seroconversion rate. The assumed seroconversion rate of Shanchol™ is 71% for all ages which is considered in the assumption based on the clinical studies conducted in a neighboring country to Nepal (*i.e.*, India). The sample size of N=330, 360, and 245 participants per OCV-S and Shanchol™ would provide 80% power to detect non-inferiority of OCV-S in terms of seroconversion rates of 75%, 71%, and 84% in adults, adolescents, and young children, respectively. The assumed non-inferiority margin is -10% based on WHO TRS 924. One sided test of non-inferiority is used with significance level of 0.025.

The sample size of N=330 per lot of OCV-S in adults will also provide >90% power for three equivalence tests of GMT ratio of immunogenicity among three lots of OCV-S (A vs. B, B vs. C and C vs. A) with overall two-sided significance level of 0.05. Each of two-sided equivalence test have >90% power to show the equivalence of GMT of immunogenicity of OCV-S with two-sided significance level of 0.05. The equivalence margin of ratio was assumed as [0.5, 2.0] according to precedent study of OCV (i.e., Euvichol®)[6] and other oral vaccine trials[20,21,22], the true GMT ratio is assumed as 1 and the coefficient of variation on titer of immunogenicity is assumed as 2.0. The coefficient of variation of GMT of OCV-S was assumed conservatively based on OCV immunogenicity data. In both sample size calculations, 10% drop out rate is assumed conservatively considering the potential large variation of experience between sites.

13.3 STATISTICAL ANALYSIS PLAN

The statistical analysis will focus on comparisons of immunogenicity of OCV-S (Group C) and Shanchol™ (Group D), and on tests of lot consistency of OCV-S (pair-wise comparison of Group A, B and C in adults) at 2 weeks post second dose of vaccination. Safety of OCV-S will be assessed by descriptively comparing incidence of common solicited, unsolicited and serious AE between OCV-S and Shanchol™ and any incidence of unexpected AE. The statistical analysis will be performed when all participants complete the Day 182 visit (24 weeks post second dose). Immunogenicity and safety data up to Day 182 will be included in the analysis.

13.4 STATISTICAL HYPOTHESES

The primary comparison to show the non-inferiority of OCV-S compared to Shanchol™ is

- Seroconversion rate of vibriocidal titers against *Vibrio cholerae* O1 Inaba and O1 Ogawa at 2 weeks after second dose of OCV-S (Group C) is non-inferior to seroconversion rate at 2 weeks after second dose of Shanchol™ (Group D) using noninferiority margin of -10%

The secondary comparison to demonstrate the lot consistency of OCV-S is

- Geometric Mean Titer of vibriocidal antibodies against *Vibrio cholerae* O1 Inaba and O1 Ogawa at 2 weeks after second dose of each lot of OCV-S is equivalent to each other (A vs. B, B vs. C, and C vs. A) using equivalence margin of GMT ratio of [0.5, 2.0]

No multiplicity adjustment of primary and secondary comparison. The non-inferiority test of OCV-S vs. Shanchol™ will be performed using one sided significance level of 0.025 and three equivalent tests will be performed using two-sided significance level of 0.05.

Following secondary comparisons are tested using the one-sided significance level of 0.025 for non-inferiority.

- GMT of vibriocidal antibodies against *Vibrio cholerae* O1 Inaba and Ogawa at 2 weeks after second dose of OCV-S (Group C) is non-inferior to GMT at 2 weeks after second dose of Shanchol™ (Group D) using non-inferiority margin of GMT ratio of 0.67
- Seroconversion rate of vibriocidal titers against *Vibrio cholerae* O1 Inaba and O1 Ogawa at 2 weeks after second dose of OCV-S (Group C) is non-inferior to seroconversion rate at 2 weeks after second dose of Shanchol™ (Group D) using noninferiority margin of -10% in each age stratum

- GMT of vibriocidal antibodies against *Vibrio cholerae* O1 Inaba and Ogawa at 2 weeks after second dose of OCV-S (Group C) is non-inferior to GMT at 2 weeks after second dose of Shanchol™ (Group D) using non-inferiority margin of GMT ratio of 0.67 in each age stratum

13.5 ANALYSIS DATASETS

The safety analysis set (SAF) includes all participants who receive at least one dose of the OCV-S or Shanchol™. All safety analyses are based on the SAF.

The Full Analysis Set (FAS) is a modified intention to treat (mITT) analysis set includes all participants who receive at least one dose of OCV-S or Shanchol™ and have at least one post-baseline immunogenicity data available.

The per-protocol analysis set (PPS) is comprised of subset of the FAS who receive all their planned vaccine administrations and who have no SMM-assessed important protocol deviations. Analyses on the PPS will be considered for the primary analysis of the immunogenicity endpoints. Participants excluded from the full analysis set for the PPS will be identified and documented prior to locking the study database.
A sensitivity analysis using the FAS will be conducted for the immunogenicity endpoints.

Demographic and baseline characteristics are presented in both PPS and FAS.

13.6 DESCRIPTION OF STATISTICAL METHODS

13.6.1 GENERAL APPROACH

This is a randomized, observer-blinded phase III study in healthy participants aged 1 to 40 years old at the time of vaccination of investigational vaccine to assess immune non-inferiority and safety of OCV-S (Group C) compared to Shanchol™ (Group D) and lot consistency among three lots of OCV-S (Group A, B, and C) in adults.
Unless otherwise specified, standard descriptive statistics will be computed for all endpoints and other observed values. The standard descriptive statistics for continuous variables include number of observations analyzed, mean, standard deviation, median, minimum, and maximum. The standard descriptive statistics for categorical variables include frequency distribution with the

number and percent of participants included in each category. The binary outcome measures of safety and immunogenicity will be summarized with frequency, proportion and associated 95% confidence interval. The continuous outcome measures of immunogenicity will be summarized with geometric mean and associated 95% confidence interval. The outcome measures will be summarized by study group and for overall ages and by age strata.

Statistical significance will be compared using two-sample t-test or ANOVA test for continuous variables, and Chi-square test or Fisher's exact test for categorical variables.

Missing immunogenicity data will not be imputed for the analysis. If missing data is more than 10%, the analysis of missing pattern will be assessed, and a multiple imputation technique will be utilized as a sensitivity analysis.

13.6.2 BASELINE DESCRIPTIVE STATISTICS

Demographic characteristics and other baseline data of participants enrolled will be tabulated by study group and for overall age and by age strata. Continuous variables (*i.e.*, age, height and weight) and categorical variables (*i.e.*, sex) will be summarized by standard descriptive statistics.

13.6.3 PRIMARY IMMUNOGENICITY ENDPOINT ANALYSIS

The primary immunogenicity endpoint will be measured as seroconversion rate (the proportion of participants with at least 4-fold rise vibriocidal titers against *Vibrio cholerae* O1 Inaba and O1 Ogawa compared prior to dosing (Day 0)) of OCV-S (Group C) and Shanchol™ (Group D) after 2 weeks of second dose. The primary immunogenicity endpoint analysis will be done by generalized linear model adjusting for stratification and any imbalances in baseline characteristics. The two-sided 95% CI of seroconversion rate for each vaccine group and the difference will be provided an estimate and non-inferiority of OCV-S will be confirmed if the lower limit of the two-sided 95% CI of the difference of seroconversion rate of OCV-S (Group C) and Shanchol™ (Group D) is greater than the pre-defined non-inferiority margin of -10%.

Primary immunogenicity endpoint analysis will be conducted on the participants in the PPS and FAS.

13.6.4 PRIMARY SAFETY ENDPOINTS ANALYSIS

The primary safety endpoint analyses are safety analyses of treatment emergent adverse events (TEAEs). TEAEs are defined for this trial as any AEs/ SAEs that occur on or after Day 0 following OCV-S or Shanchol™ administration. All TEAEs will be summarized by frequency, percentage and associated 95% confidence interval. The frequencies will also be presented separately by dose and will be depicted by system order class and preferred term. Additional frequencies will be presented with respect to maximum severity and relationship to investigational product. All serious TEAEs will also be summarized as above. Serious TEAEs will be presented in listings.

13.6.5 SECONDARY ENDPOINTS ANALYSIS

GMT of vibriocidal antibodies against *Vibrio cholerae* O1 Inaba and O1 Ogawa at 2 weeks post second dose will be calculated by vaccine group and for all ages and by age strata. The two-sided 95% CI of GMT and ratio of GMTs will be provided. The non-inferiority of OCV-S will be confirmed if the lower limit of two-tailed 95% CI of the ratio of GMT of OCV-S (Group C) to Shanchol™ (Group D) is greater than the non-inferiority margin of 0.67.

Seroconversion rate of vibriocidal antibodies against *Vibrio cholerae* O1 Inaba and O1 Ogawa at 2 weeks post second dose will be calculated by vaccine group by age strata. The two-sided 95% CI of seroconversion rate and difference of seroconversion rates will be provided. The non-inferiority of OCV-S will be confirmed if the lower limit of the two-sided 95% CI of the difference of seroconversion rate of OCV-S (Group C) and Shanchol™ (Group D) is greater than the pre-defined non-inferiority margin of -10%.

To assess lot consistency, three equivalence tests (Group A vs B, A vs C, B vs C) on GMT of vibriocidal antibody responses against *Vibrio cholerae* O1 Inaba and O1 Ogawa at 2 weeks after second dose of 3 lots of OCV-S will be performed with two-sided significance level of 0.05. The equivalence of GMT at 2 weeks after second dose of two lots of OCV-S will be analyzed using the analysis of variance (or covariance if necessary) model after log transformation. The equivalence of two lots will be confirmed if both limits of the two-sided 95% confidence interval of the ratio of the GMT between two lots of OCV-S is within the equivalence margin of [0.5, 2.0].

Secondary immunogenicity endpoints analysis will be conducted on the participants in the PPS and FAS.

13.6.6 ADHERENCE AND RETENTION ANALYSES

Summaries of Participants Disposition will be based on safety analysis set (SAF). A flow diagram of participant disposition (CONSORT flow diagram) will illustrate the progress of participants through the study duration from initial screening for eligibility to the completion of the primary outcome assessment. Number and percentage by vaccine group will be given for participants in the FAS and PP analysis sets, and reasons for study discontinuation.

13.6.7 PLANNED INTERIM ANALYSIS

No interim analysis is performed.

13.6.8 ADDITIONAL SUB-GROUP ANALYSIS

The immunogenicity analyses will be repeated by age strata. A potential difference in safety and immunogenicity by 'sex' and 'age strata' may be investigated.

13.6.9 MULTIPLE COMPARISON/MULTIPLICITY

No multiplicity adjustment is taken relative to non-inferiority and lot consistency tests.

13.6.10 EXPLORATORY ANALYSES

Seroconversion rate of vibriocidal antibody responses against *Vibrio cholerae* O1 Inaba and O1 Ogawa at 2 weeks post second dose of will be calculated by OCV-S (Group A, B, and C in adults) vaccine group. The two-sided 95% CI of seroconversion rate and difference of seroconversion rates will be provided.

Seroconversion rate and GMT of vibriocidal antibody responses at 2 weeks after first dose of OCV-S or Shanchol^{TM} titers against *Vibrio cholerae* O1 Inaba and O1 Ogawa will be calculated. The two-sided 95% CI of seroconversion rate and the difference of seroconversion rates and GMT and ratio of GMTs will be provided by vaccine group for all ages and by age strata.

14. SOURCE DOCUMENTS AND ACCESS TO SOURCE DOCUMENTS

Data recorded on the electronic Case Report Forms (eCRF) will be verified by checking the eCRF entries against source documents (i.e., all original records, medical records, diary cards, memory aids, etc.) in order to ensure data completeness and accuracy as required by study protocol. Source documents will be stored at the clinical site in a secured place under lock and key. The investigator and/or site staff must make eCRFs and source documents of participants enrolled in this study available for inspection by IVI clinical team, clinical research associate (CRA) or its representative at the time of each monitoring visit.

At a minimum, source documentation must be available to substantiate participant identification, eligibility and participation, proper informed consent procedures, dates of visits, adherence to protocol procedures, adequate reporting and follow-up of adverse events, administration of concomitant medication, study vaccine receipt/dispensing/return records, study vaccine administration information, and date of completion and reason. Specific items required as source documents will be reviewed with the investigators before the study.

The source documents must also be available for inspection, verification and copying, as required by regulations, by officials of the regulatory health authorities (e.g., NRAs, others) and/or site IRB/IECs and for possible audit by IVI Quality Management, Regulatory agency, notified body and collaborators/donors. The investigator and study site staff must comply with applicable privacy, data protection and medical confidentiality laws for use and disclosure of information related to the study and enrolled participants.

The participant must also allow access to medical records. Each participant should be informed of this prior to the start of the study by administration of the inform consent process per ICH E6 (R2).

Each participant will have a complete source documentation of records including study logbooks, ICF, lab reports and test results for the entire study period. Appropriate source documents will be prepared by study staffs. These records must be available to the IVI and regulatory authorities upon request for review.

15. DATA HANDLING AND RECORD KEEPING

15.1 DATA COLLECTION AND MANAGEMENT RESPONSIBILITIES

Electronic Case Report Forms (eCRF) will be used for recording data for each participant enrolled in the study. The site investigators are responsible to ensure the accuracy, completeness, legibility and timeliness of the data captured in eCRF. Data captured in the eCRF derived from source documents and should remain consistent with those source documents. In case of discrepancies, data will be clarified and corrected. IVI will provide guidance to investigator on making corrections to the eCRF.

Study staff will extract all data collected in source documents and workbooks for computerization into the eCRF. Data will be entered into the electronic Case Report Form (CRF) Data will be entered into eCRFs designed on EDC system which is in compliance with industry regulations such as the FDA's 21 CFR Part 11, EMA Annex 11, GAMP5, etc. The entire data collection and handling will be monitored through the implementation of individual credentials to maintain appropriate database access and ensure database integrity. Edit checks will be programmed in the EDC system to identify data entry errors during transcription, including range and consistency checks wherever applicable.

All sequential changes made will be captured in the audit trail in the EDC system which will also provide error reports and summary reports for each activity. Data entry and cleaning will be conducted at the sites. Final data cleaning and data locking will be reviewed by IVI, and data analysis will be performed at the IVI. Unblinding of study groups will be carried out after database lock. All data will be stored in a secure data base maintained by EDC system.

15.2 STUDY RECORDS RETENTION

The site Principal Investigators (PI) will retain all study records that support eCRFs for this study (i.e., ICFs, source documents, IP dispensing records) required by sponsor and by the applicable regulations in a secure and safe facility. The PI will consult IVI representative before disposal of any study records and will notify the sponsor of any change in the location, disposition, or custody of the study files. These documents should be retained for not less than 3 years after the approval of a marketing application or at least 2 years has been elapsed since formal discontinuation of

clinical development of the investigational product. The documents will be stored as per NHRC guidelines.

If a PI retires, relocates or for other reasons withdraws from the responsibility of keeping the study records, custody must be transferred to a qualified person who will accept this responsibility. The Sponsor must be notified in writing of the name and address of the new custodian. The PI will be responsible for retaining sufficient information about each participant, *i.e.*, name, address, telephone number, and participant identifier in the study, so that the sponsor and/or other regulatory authorities may have access to this information should the need arise.

If site expresses its inability to retain those study documents beyond certain period due to space constraints or due to an unanticipated event, then IVI will take the help of external archiving vendor.

15.3 PUBLICATION AND DATA SHARING POLICY

IVI assures that the key design elements of this protocol will be posted in a publicly accessible database such as Clinicaltrials.gov. All data collected during this study will be used to support this vaccine development plan until licensure and WHO prequalification. All individual data will stay strictly confidential. Analyzed data may be presented in scientific conferences and published in peer-reviewed scientific journals. Anyone wishing to publish or present site-specific data obtained during and/or after completion of the study will conform to study site and IVI data sharing policies and then forward the publication and/or presentation for review and approval to IVI and manufacturer.

16. ETHICS/PROTECTION OF HUMAN PARTICIPANTS

16.1 REGULATORY AND ETHICAL COMPLIANCE

The investigators will ensure that this study is conducted in full conformity with the ICH E6 (R2) (Guidelines, Council for International Organizations of Medical Science (CIOMS), local country's ethical policy statement or the Declaration of Helsinki, whichever provides the most protection to human participants.

16.2 PARTICIPANT AND DATA CONFIDENTIALITY

Researchers shall adhere to the principles of transparency, legitimate purpose, and proportionality in the collection, retention, and processing of personal information.

Participant confidentiality is strictly held in trust by the participating investigators, their staff, and the sponsor and their agents. This confidentiality is extended to cover testing of biological samples in addition to the clinical information relating to participants. Therefore, the study protocol, documentation, data, and all other information generated will be held in strict confidentiality. No information concerning the study, or the data will be released to any unauthorized third party without prior written approval of the sponsor.

The study monitor, other authorized representatives of the sponsor, representatives of the IRB/IEC or pharmaceutical company supplying study product may inspect all documents and records required to be maintained by the investigator, including but not limited to, medical records (office, clinic, or hospital) and pharmacy records for the participants in this study. The clinical study site will permit access to such records.

The study participant's contact information will be securely stored at clinical site for internal use during the study. At the end of the study, all records will continue to be kept in a secure location for as long a period as dictated by local IRB/IEC and local regulations.

Individual participants and their research data will be identified by a unique study identification number. The study data entry and study management systems used by clinical site and by Data Management will be secured and password protected.

Researchers must respect participants' right to privacy. Unless required by law, the confidentiality of information shall at all times be observed. Records that link individuals to specific information shall not be released. No personal identifier will be used in any publication or communication used to support this research study. The participant's identification number will be used in the event it becomes necessary to identify data specific to a single participant.

16.3 RESEARCH USE OF STORED HUMAN SAMPLES

- Intended Use: Samples, specimens and data collected under this protocol may be used to conduct safety and immune responses evaluations to the vaccines administered. Additionally, for safety purpose if deemed necessary per medical judgment of the PI or special request from the sponsor or IRB/IECs. No genetic testing will be performed.

- Plan for Use and Storage of Biological Samples: To be eligible for this protocol, participants must be willing to allow stored specimens to be used in the future for studying infectious diseases, immune function, vaccine responses and other medical conditions.

- Storage: Samples and data will be stored at Site and IVI using codes assigned by the investigators. Data will be kept in password-protected computers. Investigators will have access to the stored samples only when proper records are made to keep sample chain of custody intact.

- Loss or Destruction of Samples, Specimens or Data: Any loss or unanticipated destruction of samples (for example, due to freezer malfunction) or data (for example, misplacing a printout of data with identifiers) that compromises the scientific integrity of the study will be reported to the site IRB/IEC in accordance with institutional policies. The site PI will also notify the site IRB/IEC if the decision is made to destroy the remaining samples.

- Disposition at the completion of the study: All stored samples will be sent to IVI Biorepository

for long term storage. Study participants who request destruction of samples will be notified of compliance with such request and all supporting details will be maintained for tracking

16.4 FUTURE USE OF STORED SPECIMENS

With the participant's approval (consent form) and as approved by NRAs, local sites and IVI IRB/IECs, the identified biological samples will be stored for 10 years at the Biorepository room in IVI for future use. The immunology lab at IVI will be attributed a code that will allow linking the biological specimens with the phenotypic data from each participant, maintaining the masking of the identity of the participants.

During the conduct of the study, an individual participant can choose to withdraw consent to have biological specimens stored for future research.

The stored samples may be used for additional assessment of cholera immunogenicity, study of possible immune correlates of protection, validation of assays, testing of new assays, and for safety purpose

17. REFERENCES

1. Weekly epidemiological record. Cholera vaccines: WHO Position Paper-August 2017, No34, 2017,92,477-500

2. WHO. Background Pager on the Integration of Oral Cholera Vaccines into Global Cholera Control Programmes. 2009.

3. Safa A, Sultana J, Dac Cam P, Mwansa JC, Kong RY. Vibrio cholerae O1 hybrid El Tor strains, Asia and Africa. Emerg Infect Dis 2008, 14:987-988.

4. Faruque SM, Albert MJ, Mekalanos JJ. Epidemiology, genetics, and ecology of toxigenic Vibrio cholerae. Microbiol Mol Biol Rev 1998, 62:1301-1314.

5. Sur D, Kanungo S, Sah B, Manna B, Ali M, Paisley AM, et al. Efficacy of a low cost, inactivated whole-cell oral cholera vaccine: results from 3 years of follow-up of a randomized, controlled trial. PLoS Negl Trop Dis 2011;5(10): e1289.

6. Baik YO, Choi SK, Olveda RM, Espos RA, Ligsay AD, Montellano MB, et al. A randomized, non-inferiority trial comparing two bivalent killed, whole cell, oral cholera vaccines (Euvichol vs Shanchol) in the Philippines. Vaccine 2015;33(46):6360–5.

7. Russo P, Ligsay AD, Olveda R, Choi SK, Kim DR, Park JY, Park JY, Syed KA, Dey A, Kim YH, et al. A randomized, observer-blinded, equivalence trial comparing two variations of Euvichol®, a bivalent killed whole-cell oral cholera vaccine, in healthy adults and children in the Philippines. Vaccine 2018, 36:4317-4324.

8. WHO. Shanchol™ PI. *https://www.who.int/immunization_standards/vaccine_quality/pq_250_cholera_1dose_shantha_insert.pdf* <Accessed on Feb 14, 2021>

9. WHO. Euvichol® PI. *https://www.who.int/immunization_standards/vaccine_quality/pq_298_euvichol_1dose_eubiologics_PI.pdf?ua=1* <Accessed on Feb 14, 2021>

10. Lina Odevall, Deborah Hong, Laura Digilio, Sushant Sahastrabuddhe, Vittal Mogasale, Yeongok Baik, Seukkeun Choi, , Julia Lynch. The Euvichol story – Development and licensure of a safe, effective and affordable oral cholera vaccine through global public private partnerships. Vaccine 36 (2018) 6606-14

11. Ali M, Nelson AR, Lopez AL, Sack DA. Updated global burden of cholera in endemic countries. PLoS Negl Trop Dis 2015,9(6)

12. Epidemiology and Disease Control Division, Nepal. National Preparedness and Response Plan for Acute Gastroenteritis/Cholera Outbreaks in Nepal

13. Chulwoo Rhee, Birendra Prasad Gupta, Bibek Kumar Lal, Jacqueline Kyungah Lim, Anh Wartel, Julia Lynch, Sushant Sahastrabuddhe. Mapping the high burden areas of cholera in Nepal for potential use of oral cholera vaccine: An analysis of data from publications and routine surveillance systems. Asian Pacific Journal of Tropical Medicine. Vol 13;3:107-114

14. Ending Cholera, A Global Roadmap to 2030. Global Task Force on Cholera Control. www.who.int/cholera/en <Accessed on Feb 14, 2021>

15. GAVI update: OCV support and vaccine Investment Strategy. GTFCC Annual Meeting. Adam Soble. 3-4 June 2018, Annecy, France.

16. Summary Report of OCV Reformulation Technical Consultation. 31 Jan 2020 Dhaka, Bangladesh

17. EuvicholR. Eubiologics. http://www.eubiologics.com/en/info/Euvichol_brochure(2016).pdf <Accessed on Feb 14, 2021>.

18. Hanif Shaikh, Julia Lynch, Jerome Kim, Jean-Louis Excler. Current and future cholera vaccines. Vaccines. Volume 38, Supplement 1.

19. Baik YO, Choi SK, Kim JW, Yang JS, Kim IY, Kim CW, Hong JH. Safety and immunogenicity assessment of an oral cholera vaccine through phase I clinical trial in Korea. J Korean Med Sci 2014; 29:494-501.

20. European Medicines Agency Evaluation of Medicines for Human Use (EMEA). Scientific discussion. RotaTeq (Sanofi Pasteur MSD, SNC), 2013

21. Food and Drug Administration (FDA). Paul Kitsutani. Clinical Review for STN 125265/0 Rotarix: Rotavirus Vaccine, Live, Oral, GlaxoSmithKline Biologicals. RotarixTM BLA Clinical Review. 2008

22. Zaman K, Kingma R, Yunus M, Straaten IV, Mekkes D, Bouwstra X, Gunale B, Kulkarni PS. Safety, immunogenicity and lot-to-lot consistency of a new Bivalent Oral Polio Vaccine (bOPV) in healthy Infants: Results of a Phase III, observer blind, randomized, controlled clinical study. Vaccine 2019;33:4275-80

23. Baek YO, Choi SK, Shin SH, Koo KH, Choi HY, Cha SB, Li YC, Yoo HJ, Lee JY, Kil KH, Kim HS, Kang MS, Kang BH, Kim KH, Bae JS. A 6-week oral toxicity study of oral cholera vaccine in sprague-dawley rats. Toxicol Res 2012; 28:225-33.

24. SAGE Working Group on Oral Cholera Vaccines, WHO, and the CDC. Background paper on whole-cell, killed, Oral Cholera Vaccines. March 31, 2017

25. Global Health Observatory (GHO) data, WHO. https://www.who.int/gho/epidemic_diseases/cholera/Cholera_005.jpg?ua=1 <Accessed on Apr 19, 2021>

26. Ali M, Nelson AR, Lopez AL, Sack DA. Updated Global Burden of Cholera in Endemic Countries. PLoS Negl Trop Dis 2015;9: e0003832.

27. Bi Q, Ferreras E, Pezzoli L, Legros D, Ivers LC, Date K, et al. Protection against cholera from killed whole-cell oral cholera vaccines: a systematic review and meta-analysis. Lancet Infect Dis 2017; S1473-3099(17):30359-6.

28. Devarahosahally et al. Repeated dose oral toxicity study of Cholvax in Sprague dawley rats, unpublished report, International Vaccine Institute, Seoul, Korea, October 2015

29. Anh DD, Canh do G, Lopez AL, et al. Safety and immunogenicity of a reformulated Vietnamese bivalent killed, whole-cell, oral cholera vaccine in adults. Vaccine 2007; 25:1149-1155.

30. Mahalanabis D, Lopez AL, Sur D, et al. A randomized, placebo-controlled trial of the bivalent killed, whole-cell, oral cholera vaccine in adults and children in a cholera endemic area in Kolkata, India. PLoS One 2008;3: e2323.

31. Sur D, Lopez AL, Kanungo S, et al. Efficacy and safety of a modified killed-whole-cell oral cholera vaccine in India: an interim analysis of a cluster-randomised, double-blind, placebo-controlled trial. The Lancet 2009; 374:1694-1702.

32. Guidance for Industry: Toxicity Grading Scale for Healthy Adult and Adolescent Volunteers Enrolled in Preventive Vaccine Clinical Trials. *https://www.fda.gov/regulatory-information/search-fda-guidance-documents/toxicity-grading-scale-healthy-adult-and-adolescent-volunteers-enrolled-preventive-vaccine-clinical* <Accessed on Apr 19, 2021>

33. User manual for the revised WHO classification for causality assessment of an adverse event following immunization, WHO/HIS/EMP/QSS. March 2013

34. Guidelines on clinical evaluation of vaccines: regulatory expectations, Fifty-second report, Geneva, World Health Organization, ECBS, TRS 924, Annex

35. ICH Harmonised guideline. Integrated addendum to ICH E6(R1): Guideline for good clinical practice E6(R2). 9 Nov 2016

36. Odevall L, Rijpkema S, Smith D, et al. Consensus meeting on international standards for oral whole cell killed cholera vaccines, 17-18 May 2018, Seoul, Republic of Korea. Vaccin Res Open J. 2019; 1(1): 72-78.

37. Global Task Force on Cholera Control (GTFCC), Oral Cholera Vaccine Working Group. Technical Note. Evidence of the risks and benefits of vaccinating pregnant women with WHO pre-qualified cholera vaccines during mass campaigns, Nov 2016, *https://www.who.int/cholera/vaccines/Risk_Benefits_vaccinating_pregnant_women_Technical_Note.pdf?ua=1* <Accessed on Apr 19, 2021>

18. APPENDICES

18.1 APPENDIX I. GRADING SCALE OF ADVERSE EVENT

Table 8. Grading scale of AE: Clinical Abnormalities

Systemic (General)	Mild (Grade 1)	Moderate (Grade 2)	Severe (Grade 3)	Potentially Life Threatening (Grade 4)
Nausea/Vomiting	No interference with activity or 1-2 episodes/24 hours	Some interference with activity or > 2 episodes/24 hours	Prevents daily activity, requires outpatient IV hydration	ER visit or Hospitalization for hypotensive shock
Diarrhea	2-3 loose stools or < 400 gms/24 hours	4-5 loose stools or 400-800 gms/24 hours	6 or more watery stools or > 800 gms/24 hours or requires outpatient IV hydration	ER visit or Hospitalization
Headache	No interference with activity	Repeated use of non-narcotic pain reliever > 24 hours or some interference with activity	Significant; any use of narcotic pain reliever or prevents daily activity	ER visit or Hospitalization
Fatigue	No interference with activity	Some interference with activity	Significant; prevents daily activity	ER visit or Hospitalization
Myalgia	No interference with activity	Some interference with activity	Significant; prevents daily activity	ER visit or Hospitalization
Fever	38.0 – 38.4 ℃	38.5 – 38.9 ℃	39.0 – 40 ℃	> 40 ℃
Anorexia (loss of appetite)	No interference with activity	Some interference with activity	Significant; prevents daily activity	ER visit or hospitalization
**Systemic Illness**	**Mild (Grade 1)**	**Moderate (Grade 2)**	**Severe (Grade 3)**	**Potentially Life Threatening (Grade 4)**
Illness or clinical adverse event (as defined according to applicable regulations)	No interference with activity	Some interference with activity not requiring medical intervention	Prevents daily activity and requires medical intervention	ER visit or Hospitalization

Reference: U.S. Department of Health and Human Services, Food and Drug Administration, and Center for Biologics Evaluation and Research**.** Guidance for industry: Toxicity grading scale for healthy adult and adolescent volunteers enrolled in preventive vaccine clinical trials. Available at *https://www.fda.gov/media/73679/download*

Grade 5 (Death): Death is assigned a Grade 5 severity.

Vital Signs[c]	Mild (Grade 1)	Moderate (Grade 2)	Severe (Grade 3)	Potentially Life Threatening (Grade 4)
Fever (°C)[d]<br>(°F)[d]	38.0-38.4<br>100.4-101.1	38.5-38.9<br>101.2-102.0	39.0-40.0<br>102.1-104.0	> 40.0<br>> 104.0
Tachycardia - beats per minute	101-115	116-130	> 130	Hospitalization for arrhythmia
Bradycardia - beats per minute[e]	50-54	45-49	< 45	Hospitalization for arrhythmia
Hypertension (systolic) - mm Hg	141-150	151-155	> 155	Hospitalization for malignant hypertension
Hypertension (diastolic) - mm Hg	91-95	96-100	> 100	Hospitalization for malignant hypertension
Hypotension (systolic) - mm Hg	85-89	80-84	< 80	Hospitalization for hypotensive shock
Respiratory Rate - breaths per minute	17-20	21-25	> 25	Intubation

[a] In addition to grading the measured local reaction at the greatest single diameter, the measurement should be recorded as a continuous variable.
[b] Induration/Swelling should be evaluated and graded using the functional scale as well as the actual measurement.
[c] Participant should be at rest for all vital sign measurements.
[d] No recent hot or cold beverages or smoking.
[e] When resting heart rate is between 60-100 beats per minute. Use clinical judgment when characterizing bradycardia among some healthy participant populations, for example, conditioned athletes.

Table S1. Composition of Euvichol-S, Euvichol-Plus and Shanchol^{TM}

Serotype	Biotype	Strain	Inactivated method	Euvichol-S (LEU)	Euvichol-Plus (LEU)	Shanchol^{TM} (LEU)
O1 Inaba	Classical	Cairo 48	Heat	-	300	300
O1 Ogawa	Classical	Cairo 50	Heat	-	300	300
O1 Ogawa	Classical	Cairo 50	Formalin	600	300	300
O1 Inaba	El Tor	Phil 6973	Formalin	900	600	600
O139	-	4260B	Formalin	-	600	600
			**Total (LEU)**			
			**O1 Inaba**	900	900	900
			**O1 Ogawa**	600	600	600
			**O1 (Inaba+Ogawa)**	1500	1500	1500
			**O1 (Inaba+Ogawa) + O139**	-	2100	2100

Abbreviations: LEU= Lipopolysaccharide ELISA Unit.

Table S2. Demographic Characteristics stratified by age (Full analysis set)

Age strata: 18-40 years

Characteristics	Total (N=1296)	Group A [a] (N=327)	Group B [a] (N=322)	Group C [a] (N=323)	Group D [a] (N=324)	p-Value [b]
Sex						0.3217
Male (%)	599 (46.2%)	147 (44.95%)	159 (49.38%)	140 (43.34%)	153 (47.22%)	
Female (%)	697 (53.8%)	180 (55.05%)	163 (50.62%)	183 (56.55%)	171 (52.78%)	
Age (years)						
Mean (SD)	26.1 (6.4)	25.55 (6.35)	26.50 (6.00)	26.62 (6.68)	25.69 (6.39)	0.0734
Median (Q1, Q3)	25.0 (20.0, 31.0)	24.00 (20.00, 31.00)	26.00 (21.00, 31.00)	25.00 (21.00, 33.00)	24.00 (20.00, 30.00)	0.0913
Height (cm)						
Mean (SD)	158.6 (8.7)	158.78 (8.99)	158.87 (8.68)	158.15 (9.10)	158.48 (8.21)	0.6215
Median (Q1, Q3)	158.0 (152.0, 165.0)	158.00 (152.00, 165.00)	158.00 (153.00, 165.00)	157.50 (152.00, 165.00)	158.00 (152.00, 164.00)	0.6109
Weight (kg)						
Mean (SD)	56.80(11.1)	56.67 (10.88)	57.66 (11.29)	57.12 (11.33)	55.76 (10.98)	0.1209
Median (Q1, Q3)	55.0 (48.60, 63.55)	55.50 (48.40, 62.40)	56.10 (50.00, 64.90)	55.20 (48.80, 65.00)	53.70 (48.00, 61.40)	0.0670
BMI (kg/m^2)						
Mean (SD)	22.6 (4.0)	22.48 (3.96)	22.82 (4.01)	22.83 (4.13)	22.16 (3.81)	0.0309
Median (Q1, Q3)	21.8 (19.6, 24.9)	21.92 (19.47, 24.73)	22.14 (19.94, 24.96)	21.97 (19.74, 25.54)	21.31 (19.50, 24.15)	0.0320

Age strata: 6-17 years

Characteristics	Total (N=714)	Group C [a] (N=359)	Group D [a] (N=355)	p-Value [b]
Sex				0.0239
Male (%)	378 (52.94%)	175 (48.75%)	203 (57.18%)	
Female (%)	336 (47.06%)	184 (51.25%)	152 (42.82%)	
Age (years)				
Mean (SD)	10.54 (3.21)	10.62 (3.16)	10.46 (3.26)	0.5154
Median (Q1, Q3)	10.00 (8.00, 13.00)	10.00 (8.00, 13.00)	10.00 (8.00, 13.00)	0.4496
Height (cm)				
Mean (SD)	136.61 (16.73)	137.44 (16.18)	135.76 (17.25)	0.1811
Median (Q1, Q3)	136.00 (123.00, 150.00)	136.00 (124.50, 150.00)	135.00 (120.00, 150.00)	0.1889
Weight (kg)				
Mean (SD)	32.74 (11.68)	33.01 (11.33)	32.47 (12.04)	0.5381
Median (Q1, Q3)	30.00 (22.90, 41.60)	30.50 (23.70, 41.50)	30.00 (22.20, 42.10)	0.3104
BMI (kg/m^2)				
Mean (SD)	16.96 (2.95)	16.94 (2.91)	19.98 (3.00)	0.8413
Median (Q1, Q3)	16.33 (14.86, 18.58)	16.34 (14.80, 18.52)	16.31 (14.95, 18.66)	0.9744

Age strata: 1-5 years

Characteristics	Total (N=484)	Group C [a] (N=243)	Group D [a] (N=241)	p-Value [b]
Sex				0.3594
Male (%)	257 (53.10%)	124 (51.03%)	133 (55.19%)	
Female (%)	227 (46.90%)	119 (48.97%)	108 (44.81%)	
Age (years)				
Mean (SD)	3.05 (1.29)	3.01 (1.30)	3.09 (1.28)	0.5019
Median (Q1, Q3)	3.00 (2.00, 4.00)	3.00 (2.00, 4.00)	3.00 (2.00, 4.00)	0.5036
Height (cm)				
Mean (SD)	94.29 (11.00)	93.85 (10.74)	94.73 (11.26)	0.3780
Median (Q1, Q3)	94.00 (86.00, 102.00)	94.00 (85.50, 101.00)	94.00 (86.00, 103.00)	0.4543
Weight (kg)				
Mean (SD)	14.27 (3.39)	14.31 (3.48)	14.24 (3.31)	0.8155
Median (Q1, Q3)	14.00 (11.80, 16.30)	14.00 (11.90, 16.00)	13.80 (11.60, 16.50)	0.8847
BMI (kg/m^2)				
Mean (SD)	15.98 (2.26)	16.16 (2.37)	15.79 (2.13)	0.0774
Median (Q1, Q3)	15.68 (14.77, 16.67)	15.83 (14.71, 16.37)	15.48 (14.81, 17.01)	0.0444

Abbreviations: BMI=body mass index; SD=standard deviation.

[a] Participants in Groups A, B and C received 3 different lots of Euvichol-S and those in Group D received Shanchol™.

[b] Comparison between Group C and Group D. For continuous variables (i.e., age and BMI), p-value was calculated by 2 sample t-test for mean and by Wilcoxon rank-sum test for median. For categorical variable (i.e., Sex), Chi–square test was used. Fisher's test was used if more than 20% of expected cell frequency was <5.

Table S3. Lot-to-lot consistency (Per-protocol analysis set)

18-40 years	O1 Inaba					O1 Ogawa				
	**Group A (N=308)**	**Group B (N=311)**	**Group C (N=312)**	**Adjusted comparison**	**p-Value**	**Group A (N=308)**	**Group B (N=311)**	**Group C (N=312)**	**Adjusted comparison**	**p-Value**
**GMT[a]**										
**Baseline**<br>GMT [95% CI]	30.84<br>[23.41, 40.63]	24.58<br>[18.68, 32.34]	22.90<br>[17.41, 30.11]	 1.25<br>[0.85, 1.85] [c,e]<br>1.35<br>[0.91, 1.99] [c,f]<br>1.07<br>[0.73, 1.58] [c,g]	0.2925 [h,j]<br>0.2529 [h,e]<br>0.1331 [h,f]<br>0.7195 [h,g]	58.31<br>[43.77, 77.69]	39.24<br>[29.49, 52.21]	53.94<br>[40.56, 71.73]	 1.49<br>[0.99, 2.23] [c,e]<br>1.08<br>[0.72, 1.62] [c,f]<br>0.73<br>[0.49, 1.09] [c,g]	0.1261 [h,j]<br>0.0551 [h,e]<br>0.7052 [h,f]<br>0.1221 [h,g]
**2 weeks post first dose**<br>GMT [95% CI]	756.84<br>[617.09, 928.25]	711.33<br>[580.64, 871.43]	742.04<br>[605.86, 908.82]	 1.06<br>[0.80, 1.42] [c,e]<br>1.02<br>[0.76, 1.36] [c,f]<br>0.96<br>[0.72, 1.28] [c,g]	0.9108 [h,j]<br>0.6726 [h,e]<br>0.8929 [h,f]<br>0.7726 [h,g]	1034.22<br>[864.19, 1237.71]	857.57<br>[717.10, 1025.55]	963.13<br>[805.78, 1151.20]	 1.21<br>[0.94, 1.55] [c,e]<br>1.07<br>[0.83, 1.38] [c,f]<br>0.89<br>[0.69, 1.15] [c,g]	0.3434 [h,j]<br>0.1478 [h,e]<br>0.5808 [h,f]<br>0.3676 [h,g]
**2 weeks post second dose**<br>GMT [95% CI]	666.66<br>[560.72, 792.62]	606.39<br>[510.52, 720.27]	658.79<br>[554.76, 782.34]	 1.10<br>[0.86, 1.40] [c,e]<br>1.01<br>[0.79, 1.29] [c,f]<br>0.92<br>[0.72, 1.17] [c,g]	0.7077 [h,j]<br>0.4464 [h,e]<br>0.9239 [h,f]<br>0.5037 [h,g]	929.02<br>[795.72, 1084.64]	801.96<br>[687.33, 935.72]	952.12<br>[816.38, 1110.42]	 1.16<br>[0.93, 1.44] [c,e]<br>0.98<br>[0.78, 1.21] [c,f]<br>0.84<br>[0.68, 1.05] [c,g]	0.2489 [h,j]<br>0.1875 [h,e]<br>0.8252 [h,f]<br>0.1226 [h,g]
**Seroconversion[b]**										
**2 weeks post first dose**<br>Seroconversion (%)<br>[95% CI]	249 (80.72)<br>[76.71, 84.74]	251 (80.91)<br>[76.85, 84.97]	249 (78.74)<br>[74.55, 82.93]	 -0.21<br>[-5.61, 5.18] [d,e]<br>1.98<br>[-3.73, 7.68] [d,f]<br>2.19<br>[-3.37, 7.75] [d,g]	0.7034 [i,j]<br>0.9380 [i,e]<br>0.4971 [i,f]<br>0.4394 [i,g]	240 (77.24)<br>[73.40, 81.07]	234 (74.12)<br>[69.81, 78.44]	242 (76.62)<br>[72.71, 80.52]	 3.07<br>[-2.19, 8.32] [d,e]<br>0.63<br>[-4.32, 5.59] [d,f]<br>-2.42<br>[-7.71, 2.86] [d,g]	0.4953 [i,j]<br>0.2530 [i,e]<br>0.8024 [i,f]<br>0.3691 [i,g]
**2 weeks post second dose**<br>Seroconversion (%)<br>[95% CI]	235 (77.20)<br>[73.31, 81.08]	245 (77.51)<br>[73.77, 81.26]	246 (76.38)<br>[72.56, 80.19]	 -0.22<br>[-4.83, 4.38] [d,e]<br>0.81<br>[-4.10, 5.72] [d,f]<br>1.14<br>[-3.73, 6.01] [d,g]	0.8878 [i,j]<br>0.9240 [i,e]<br>0.7457 [i,f]<br>0.6467 [i,g]	221 (69.59)<br>[65.94, 73.25]	225 (68.69)<br>[64.84, 72.54]	224 (70.66)<br>[67.12, 74.20]	 0.92<br>[-3.38, 5.21] [d,e]<br>-1.07<br>[-4.89, 2.74] [d,f]<br>-1.92<br>[-5.82, 1.98] [d,g]	0.6250 [i,j]<br>0.6753 [i,e]<br>0.5817 [i,f]<br>0.3342 [i,g]

Abbreviations: CI=confidence interval; GMT=geometric mean titers; N=number of participants in per-protocol analysis set with non-missing results.

[a] The GMT ratio and its 95% CI were calculated as the antilogarithmic of the difference between the mean of the log-transformed data in the test arm and that in the comparator arm with baseline titer adjustment (covariates) in a generalized linear model. Equivalence of GMT ratio between lots of Euvichol-S is confirmed using margin of [0.5, 2.0].

[b] The estimated seroconversion rate, 95% CI, and p-values were derived using the generalized linear model (link=identity) for binomial distribution with treatment group as covariate by adjustment for baseline titers. Equivalence of seroconversion rate between lots of Euvichol-S is confirmed using margin of [-10%, 10%].

[c] Comparison=$GMT_{\text{Group C}}/GMT_{\text{Group D}}$

[d] Comparison=Group C – Group D

[e] Comparisons between Groups A and B

[f] Comparisons between Groups A and C

[g] Comparisons between Groups B and C

[h] The p-value was testing of no significant difference (fold difference=1) in GMT values across the treatment groups using generalized linear model.

[i] The p-value was testing of no significant difference (difference=0%) in seroconversion rate across the treatment groups using generalized linear model (link=identity).

[j] Comparison across Groups A, B, and C

Table S4. Summary of overall adverse events (Safety analysis set)

Age Strata: Overall

	**Total (N=2529)**		**Group A (N=330)**		**Group B (N=331)**		**Group C (N=934)**		**Group D (N=934)**		
**Statistics**	**n (%)**	**m**	**n (%)**	**m**	**n (%)**	**m**	**n (%)**	**m**	**n (%)**	**m**	**p-value**
Any TEAE post 1st vaccination	138 (5.46%)	220	15 (4.55%)	21	16 (4.83%)	24	61 (6.53%)	98	46 (4.93%)	77	0.1353
Immediate Reaction within 30 mins post 1st vaccination	2 (0.08%)	2	1 (0.30%)	1	0 (0.00%)	0	1 (0.11%)	1	0 (0.00%)	0	>0.9999
Solicited AEs within 7 days post 1st vaccination	86 (3.40%)	118	11 (3.33%)	12	12 (3.63%)	18	37 (3.96%)	52	26 (2.78%)	36	0.1586
Unsolicited AEs within 28 days post 1st vaccination	69 (2.73%)	101	5 (1.52%)	8	5 (1.51%)	6	33 (3.53%)	46	26 (2.78%)	41	0.3544
SAEs post 1st vaccination	2 (0.08%)	2	1 (0.30%)	1	0 (0.00%)	0	1 (0.11%)	1	0 (0.00%)	0	>0.9999
Severity post 1st vaccination											
Mild	112 (4.43%)	162	10 (3.03%)	12	15 (4.53%)	20	49 (5.25%)	71	38 (4.07%)	59	0.2271
Moderate	38 (1.50%)	46	6 (1.82%)	7	4 (1.21%)	4	14 (1.50%)	18	14 (1.50%)	17	>0.9999
Severe	9 (0.36%)	12	2 (0.61%)	2	0 (0.00%)	0	6 (0.64%)	9	1 (0.11%)	1	0.1243
Potentially Life-threatening	0 (0.00%)	0	0 (0.00%)	0	0 (0.00%)	0	0 (0.00%)	0	0 (0.00%)	0	NA
Death	0 (0.00%)	0	0 (0.00%)	0	0 (0.00%)	0	0 (0.00%)	0	0 (0.00%)	0	NA
Relatedness post 1st vaccination											
Definitely Related	16 (0.63%)	19	4 (1.21%)	4	2 (0.60%)	5	7 (0.75%)	7	3 (0.32%)	3	0.2047
Probably Related	34 (1.34%)	46	4 (1.21%)	4	4 (1.21%)	6	18 (1.93%)	24	8 (0.86%)	12	0.0483
Possibly Related	40 (1.58%)	53	4 (1.21%)	4	5 (1.51%)	6	16 (1.71%)	21	15 (1.61%)	22	0.8563
Unlikely to be Related	50 (1.98%)	66	2 (0.61%)	2	1 (0.30%)	1	27 (2.89%)	35	20 (2.14%)	28	0.3011
Not Related	23 (0.91%)	36	5 (1.52%)	7	5 (1.51%)	6	6 (0.64%)	11	7 (0.75%)	12	0.7808
Any TEAE post 2nd vaccination	123 (4.86%)	183	8 (2.42%)	11	6 (1.81%)	9	54 (5.78%)	84	55 (5.89%)	79	0.9214
Immediate Reaction within 30 mins post 2nd vaccination	3 (0.12%)	4	0 (0.00%)	0	0 (0.00%)	0	1 (0.11%)	1	2 (0.21%)	3	>0.9999
Solicited AEs within 7 days post 2nd vaccination	55 (2.17%)	71	7 (2.12%)	9	2 (0.60%)	2	15 (1.61%)	21	31 (3.32%)	39	0.0169

Age Strata: Overall

Statistics	Total (N=2529) n (%)	m	Group A (N=330) n (%)	m	Group B (N=331) n (%)	m	Group C (N=934) n (%)	m	Group D (N=934) n (%)	m	p-value
Unsolicited AEs within 28 days post 2nd vaccination	88 (3.48%)	112	1 (0.30%)	2	5 (1.51%)	7	46 (4.93%)	63	36 (3.85%)	40	0.2587
SAEs post 2nd vaccination	1 (0.04%)	1	0 (0.00%)	0	0 (0.00%)	0	0 (0.00%)	0	1 (0.11%)	1	>0.9999
Severity post 2nd vaccination											
Mild	99 (3.91%)	137	6 (1.82%)	8	2 (0.60%)	2	47 (5.03%)	66	44 (4.71%)	61	0.7471
Moderate	30 (1.19%)	35	1 (0.30%)	1	4 (1.21%)	6	10 (1.07%)	12	15 (1.61%)	16	0.3141
Severe	9 (0.36%)	11	1 (0.30%)	2	1 (0.30%)	1	5 (0.54%)	6	2 (0.21%)	2	0.4522
Potentially Life-threatening	0 (0.00%)	0	0 (0.00%)	0	0 (0.00%)	0	0 (0.00%)	0	0 (0.00%)	0	NA
Death	0 (0.00%)	0	0 (0.00%)	0	0 (0.00%)	0	0 (0.00%)	0	0 (0.00%)	0	NA
Relatedness post 2nd vaccination											
Definitely Related	7 (0.28%)	9	2 (0.61%)	3	0 (0.00%)	0	3 (0.32%)	4	2 (0.21%)	2	>0.9999
Probably Related	12 (0.47%)	13	3 (0.91%)	3	0 (0.00%)	0	2 (0.21%)	2	7 (0.75%)	8	0.1786
Possibly Related	37 (1.46%)	49	3 (0.91%)	3	2 (0.60%)	2	10 (1.07%)	15	22 (2.36%)	29	0.0324
Unlikely to be Related	51 (2.02%)	64	0 (0.00%)	0	1 (0.30%)	1	28 (3.00%)	37	22 (2.36%)	26	0.3897
Not Related	38 (1.50%)	48	1 (0.30%)	2	4 (1.21%)	6	19 (2.03%)	26	14 (1.50%)	14	0.3798
Any TEAE post any vaccination	244 (9.65%)	403	22 (6.67%)	32	22 (6.65%)	33	107 (11.46%)	182	93 (9.96%)	156	0.2948
Immediate Reaction within 30 mins post any vaccination	4 (0.16%)	6	1 (0.30%)	1	0 (0.00%)	0	1 (0.11%)	2	2 (0.21%)	3	>0.9999
Solicited AEs within 7 days post any vaccination	136 (5.38%)	189	17 (5.15%)	21	14 (4.23%)	20	51 (5.46%)	73	54 (5.78%)	75	0.7631
Unsolicited AEs within 28 days post any vaccination	149 (5.89%)	213	6 (1.82%)	10	10 (3.02%)	13	75 (8.03%)	109	58 (6.21%)	81	0.1261
SAEs post any vaccination	3 (0.12%)	3	1 (0.30%)	1	0 (0.00%)	0	1 (0.11%)	1	1 (0.11%)	1	>0.9999
Severity post any vaccination											
Mild	196 (7.75%)	299	16 (4.85%)	20	17 (5.14%)	22	88 (9.42%)	137	75 (8.03%)	120	0.2865
Moderate	68 (2.69%)	81	7 (2.12%)	8	8 (2.42%)	10	24 (2.57%)	30	29 (3.10%)	33	0.4860

Age Strata: Overall

	Total (N=2529)		Group A (N=330)		Group B (N=331)		Group C (N=934)		Group D (N=934)		
**Statistics**	**n (%)**	**m**	**n (%)**	**m**	**n (%)**	**m**	**n (%)**	**m**	**n (%)**	**m**	**p-value**
Severe	18 (0.71%)	23	3 (0.91%)	4	1 (0.30%)	1	11 (1.18%)	15	3 (0.32%)	3	0.0319
Potentially Life-threatening	0 (0.00%)	0	0 (0.00%)	0	0 (0.00%)	0	0 (0.00%)	0	0 (0.00%)	0	NA
Death	0 (0.00%)	0	0 (0.00%)	0	0 (0.00%)	0	0 (0.00%)	0	0 (0.00%)	0	NA
Relatedness post any vaccination											
Definitely Related	21 (0.83%)	28	5 (1.52%)	7	2 (0.60%)	5	9 (0.96%)	11	5 (0.54%)	5	0.2832
Probably Related	46 (1.82%)	59	7 (2.12%)	7	4 (1.21%)	6	20 (2.14%)	26	15 (1.61%)	20	0.3936
Possibly Related	76 (3.01%)	102	7 (2.12%)	7	7 (2.11%)	8	26 (2.78%)	36	36 (3.85%)	51	0.1965
Unlikely to be Related	94 (3.72%)	130	2 (0.61%)	2	2 (0.60%)	2	52 (5.57%)	72	38 (4.07%)	54	0.1304
Not Related	60 (2.37%)	84	6 (1.82%)	9	9 (2.72%)	12	24 (2.57%)	37	21 (2.25%)	26	0.6508

Note: Percentages are based on safety analysis set. The safety analysis set (SAF) includes all participants who receive at least one dose of the OCV-S or Shanchol™.
A Treatment-Emergent AEs are defined as any AEs/SAEs that occur on or after Day 0 following OCV-S or Shanchol™ administration.

Group A, B and C are three different lots of OCV-S and Group D is Shanchol™, respectively.
"p-value" is the comparison between the number of subjects in Group C and Group D.
Chi-square test is used to calculate "p-value". Fisher's test is used if more than 20% of expected cell frequency is <5.

Abbreviations: n = Number of Subjects with Adverse Events; m = Number of Adverse Events; AE = Adverse Events; TEAE = Treatment Emergent Adverse Events; SAE = Serious Adverse Events.

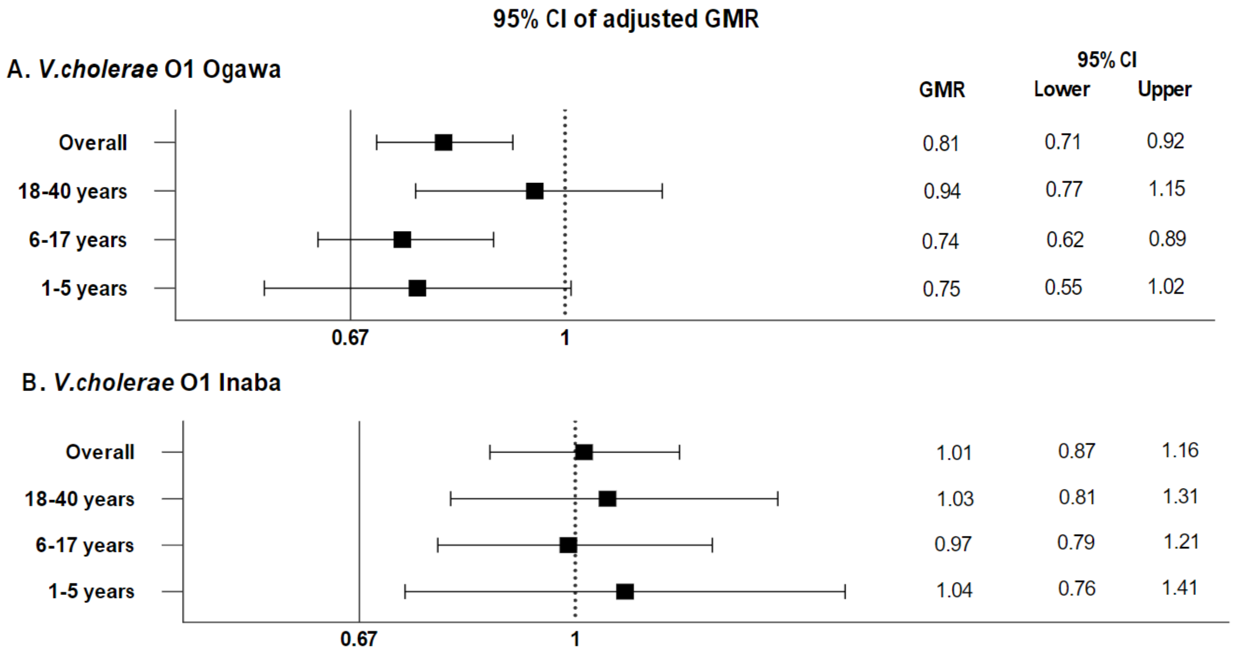

Figure S1. 95% Confidence Intervals (CI) of adjusted Geometric Mean Ratio (GMR) (Euvichol-S/ Shanchol™) for *V.cholerae* O1 Ogawa (A) and Inaba (B)

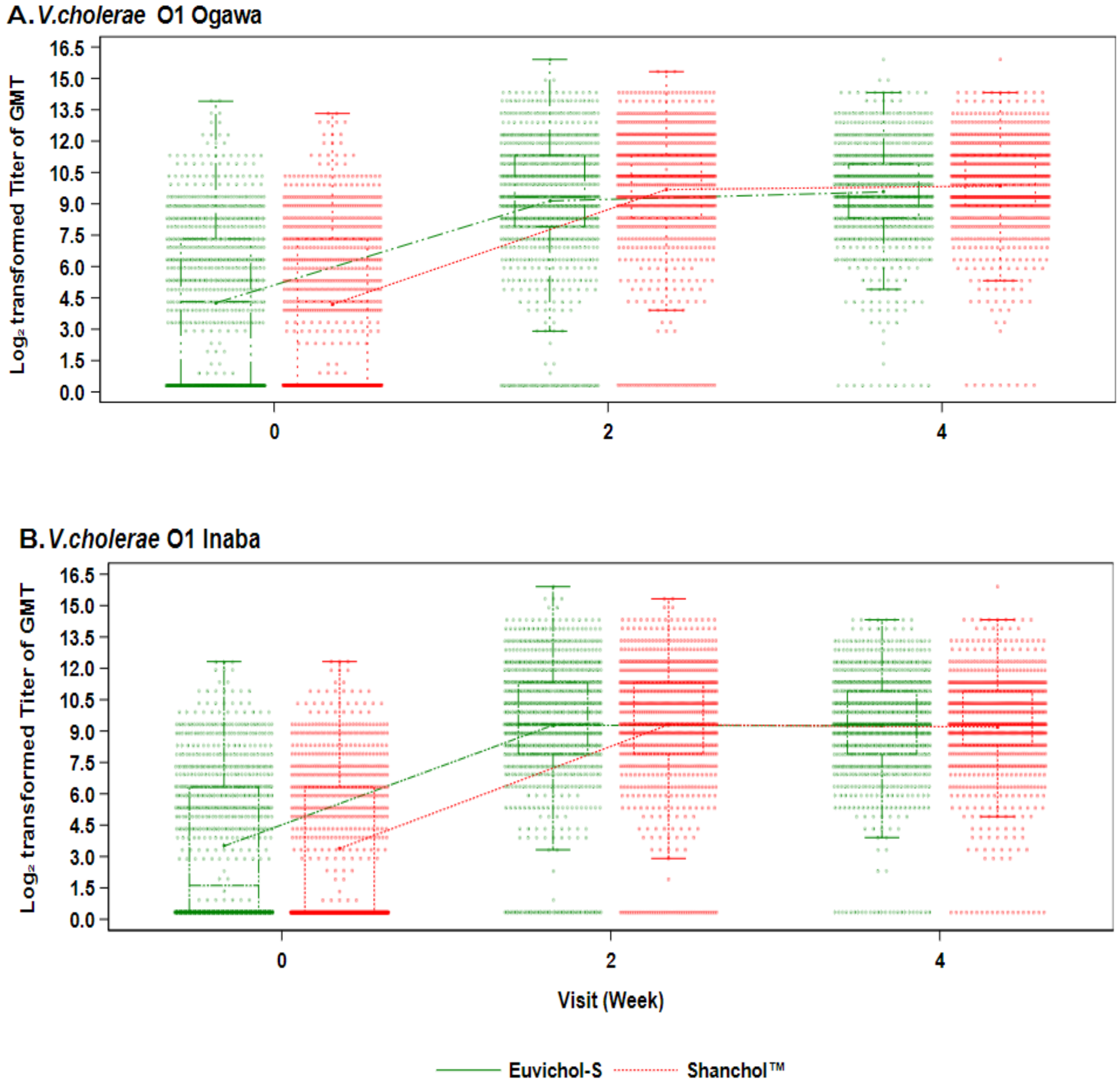

Figure S2. Log-transformed Geometric Mean Titer (GMT) of Euvichol-S (Group C) and Shanchol^{TM} (Group D) at Week 0, 2 and 4 for *V.cholerae* O1 Ogawa (A) and Inaba (B) in overall ages

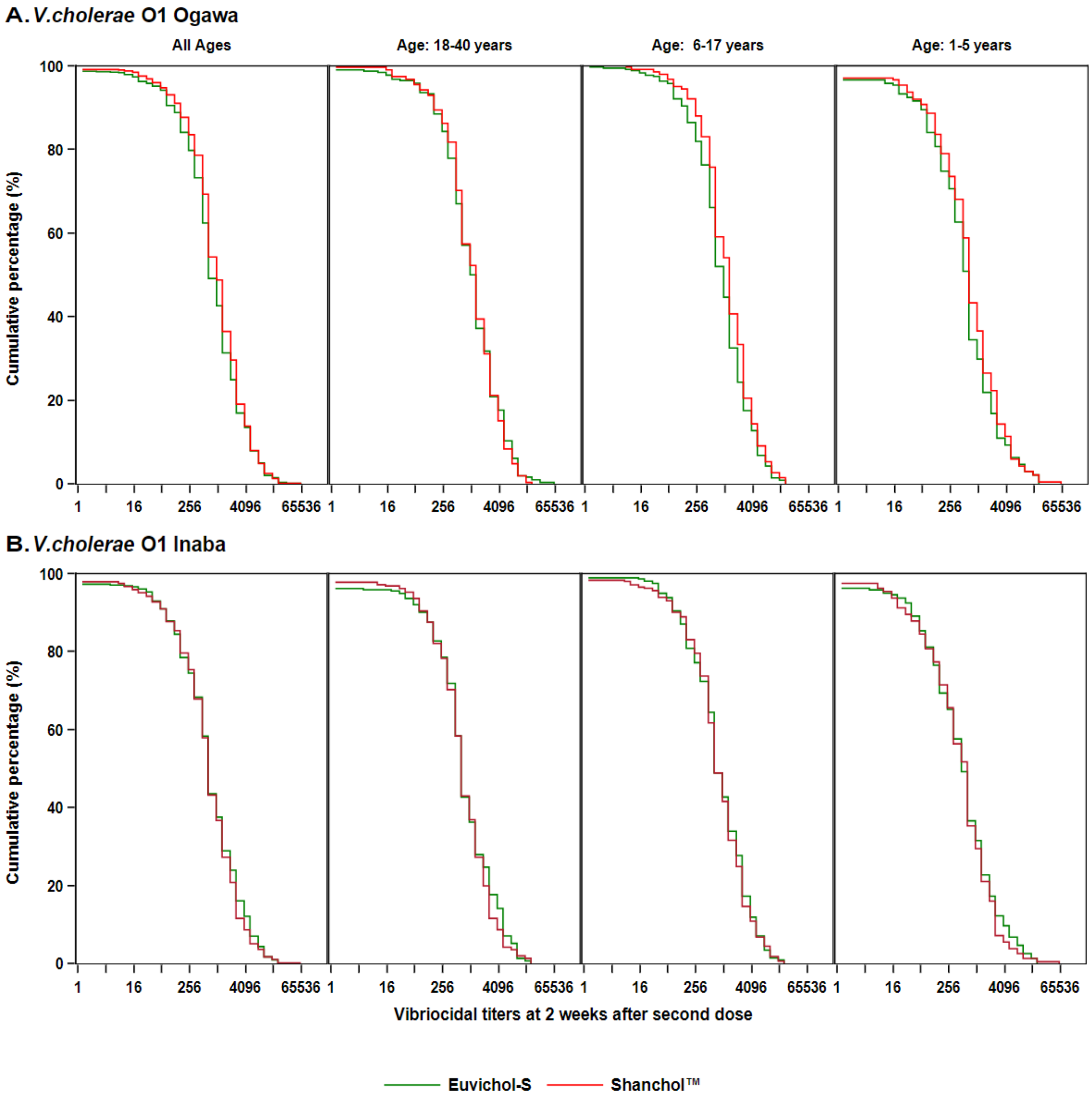

Figure S3. Reverse Cumulative Distribution Curve (RCDC) of cumulative distribution of anti-*V.cholerae* O1 Ogawa (A) and Inaba (B) vibriocidal titer at 2 weeks after the second dose in PPS analysis.
