# Supplementary material for: Safety and immunogenicity of the Euvichol-S oral cholera vaccine for prevention of Vibrio cholerae O1 infection in Nepal: an observer-blind, active-controlled, randomised, non-inferiority, phase 3 trial
Source: Lancet Glob Health. 2024 Apr 11;12(5):e826–37. doi: 10.1016/S2214-109X(24)00059-7 (PMC11027156; doi:10.1016/S2214-109X(24)00059-7)
Supplement: Equitable Partnership Declaration [file mmc3.pdf]

THE LANCET
Global Health

Supplementary appendix 3

This Equitable Partnership Declaration (EPD) was submitted by the authors, and we reproduce it as supplied. It has not been peer reviewed. *The Lancet*'s editorial processes have not been applied to the EPD.

Supplement to: Song KR, Chapagain RH, Tamrakar D, et al. Safety and immunogenicity of the Euvichol-S oral cholera vaccine for prevention of *Vibrio cholerae* O1 infection in Nepal: an observer-blind, active-controlled, randomised, non-inferiority, phase 3 trial. *Lancet Glob Health* 2024; **12:** e826–37.

Equitable Partnership Declaration questions

Researcher considerations

1. Please detail the involvement that researchers who are based in the region(s) of study had during a) study design; b) clinical study processes, such as processing blood samples, prescribing medication, or patient recruitment; c) data interpretation; and d) manuscript preparation, commenting on all aspects. If they were not involved in any of these aspects, please explain why.

   This question is intended for international partnerships; if all your authors are based in the area of study, this question is not applicable.

   This should include a thorough description of their leadership role(s) in the study. Are local researchers named in the author list or the acknowledgements, or are they not mentioned at all (and, if not, why)? Please also describe the involvement of early career researchers based in the location of the study. Some of this information might be repeated from the Contributors section in the manuscript. Note: we adhere to ICMJE authorship criteria when deciding who should be named on a paper.

**a) Study design:**<br>The study was conducted at four sites across Nepal. The four site principal investigators (PI) reviewed and contributed to the study protocol from scientific perspective as well as from regional perspective for the trial to be well implemented at the sites.
**b) Clinical study processes:**<br>Each of the study sites obtained site ethics committee approvals, enrolled over 600 participants, vaccinated, and followed all the participants. The data generated from the sites were captured in the source notes as well as in the electronic database by the site staff.<br>The coordinating PI supported obtaining the national ethics committee approval as well as regulatory approval of IND submitted. He was responsible for reporting to the regulators as per need.
**c) Data interpretation:**<br>The data analysis and interpretation for this pivotal trial was done by study team at International Vaccine Institute (IVI) including the Department of Biostatistics and Data Management.
**d) Manuscript preparation:**<br>The four site PIs contributed to reviewing of the manuscript. The coordinating PI contributed to the manuscript as a co-first author.

2. Were the data used in your study collected by authors named on the paper, or have they been extracted from a source such as a national survey? ie, is this a secondary analysis of data that were not collected by the authors of this paper. If the authors of this paper were not involved in data collection, how were data interpreted with sufficient contextual knowledge?

   The Lancet Global Health *believe contextual understanding is crucial for informed data analysis and interpretation.*

The data used in the study were collected by those who are included as authors, the PIs from the four sites.

3. How was funding used to remunerate and enhance the skills of researchers and institutions based in the area(s) of study? And how was funding used to improve research infrastructure in the area of study?

   Potentially effective investments into long-term skills and opportunities within institutions could include training or mentorship in analytical techniques and manuscript writing, opportunities to lead all or specific aspects of the study, financial remuneration rather than requiring volunteers, and other professional development and educational opportunities.

   Improvements to research infrastructure could be funding of extended trial designs (such as platform trials) and use of master protocols to enable these designs, establishment of long-term contracts for research staff, building research facilities, and local control of funding allocation.

**Skills:** With the funding received, the study staff completed the mandatory courses for the clinical trials such as GCP training, informed consent obtaining process, inspection readiness etc, and also received the study specific trainings. The study team developed the process for SOP preparation, management and reporting of serious adverse events, protocol deviations and other reports to the regulators and IRB/ethics committees. PIs and study staff received many trainings and built on the job experience through the sponsors monitoring/QC visit, regulator inspection etc.<br>The funds were utilized to set up and maintain the facility and human resources for the operation of the study at each site. It included the overhead cost to the participating institute for their contribution to physical space, water, electricity, and waste disposal. The fund was also used to pay for the cost related process and approval of local/national ethics committees, travel compensation to the participants etc.
**Research infrastructure:** Fund was also used to equip freezers for the blood sample storage and investigational product management. Computers and printers were purchased for the office management and data entry, and laboratory equipment were procured. The research physical infrastructure was supported at all four participating sites. As the study was conducted during COVID-19 pandemic era, we tried to ensure that the healthy study participants' flow did not overlap with COVID-19 patients. A new building to support clinical research was funded at BPKIHS site to ensure that the site staff can secure safe and appropriate space for the study participants and research documents.

4. How did you safeguard the researchers who implemented the study?

   Please describe how you guaranteed safe working conditions for study staff, including provision of appropriate personal protective equipment, protection from violence, and prevention of overworking.

We had SOPs for research site and strictly followed the SOP. As the trial was conducted during the COVID-19 pandemic era, all staff were thoroughly trained on the procedures and personal protective equipment were provided to all staff.<br>We had SOPs for natural disasters and any form of violence, and all staff were adequately trained. Study team regularly updated the study status to the relevant hospital administrations. The hospital administrations frequent monitoring and local IRCs also ensured the safety and protection from violence.<br>We had the fixed working hours per day. The CRO was closely observing the daily recruitments and monitoring working hours.

Benefits to the communities and regions of study

5. How does the study address the research and policy priorities of its location?

How were the local priorities determined and then used to inform the research question? Who decided which priorities to take forward? Which elements of the study address those priorities?

Diarrhoeal disease is one of the major causes of morbidity and mortality in Nepal. Nepal is a cholera endemic country and has conducted cholera vaccination campaigns in response to cholera outbreaks using the current oral cholera vaccine from the Gavi funded stockpile. The Government of Nepal, Ministry of health and population decide the research priorities. Nepal Health Research Council (NHRC) has the priorities for health research which include diarrhoeal disease and health economics as research priorities.<br>As the Euvichol-S demonstrated its safety and immune non-inferiority compared to the currently available WHO PQ vaccine, Shanchol^{TM}, we consider that Euvichol-S will contribute to the vaccine stockpile with lesser cost and improved supply which will increase the access to the vaccine for countries such as Nepal and so they can plan for preventive vaccination of their high risk communities.

6. How will research products be shared in the community of study?

For instance, will you be providing written or oral layperson summaries for non-academic information sharing? Will study data be made available to institutions in the region(s) of study? The Lancet Global Health *encourages authors to translate the summary (abstract) into relevant languages after paper editing; do you intend to translate your summary?*

We will translate the abstract to the Nepali language and publish as per Lancet Global health. And based on this Euvichol-S Phase 3 study experience, the site PIs prepared and/or published several sub-study results titled, “Conducting a Phase III Clinical Trial in Children during the COVID-19 Pandemic: Experience and Lessons Learnt from a Clinical Research Facility of Nepal”, “Parental motivation and perceived barriers for participating in pediatric vaccine clinical trial: findings from a clinical research site in Nepal” and “Knowledge and Perception of Clinical trial among the Participants: An experience from Oral Cholera Vaccine Study in eastern Nepal".

7. How were individuals, communities, and environments protected from harm?

a) *How did you ensure that sensitive patient data was handled safely and respectfully? Was there any potential for stigma or discrimination against participants arising from any of the procedures or outcomes of the study?*

The consent forms were kept separately and safe in the research site with appropriate handling independently monitored. All other data were analysed using an anonymous subject identifier. The patient population was recruited from a general population and there is no perceived stigma or discrimination against participants by the local study staff.

b) *Might any of the tests be experienced as invasive or culturally insensitive?*

There was blood sampling during the study. This can be an invasive procedure but not culturally sensitive.

c) *How did you determine that work was sensitive to traditions, restrictions, and considerations of all cultural and religious groups in the study population?*

We discussed if there would be any study procedure that might be considered culturally insensitive during the protocol development stage with IVI study team, but this was not applicable.

d) *Were biowaste and radioactive waste disposed of in accordance with local laws?*

Yes. the biowaste was disposed according to the hospital waste management guideline issued by Ministry of health and population, Nepal.

e) *Were any structures built that would have impacted members of the community or the environment (such as handwashing facilities in a public space)? If so, how did you ensure that you had appropriate community buy-in?*

We conducted the study in hospital setting and no public structure was built.

f) *How might the study have impacted existing health-care resources (such as staff workloads, use of equipment that is typically employed elsewhere, or reallocation of public funds)?*

We hired extra human resources to support the study so that the hospital staff were not overloaded with the work. Most of the main equipment for the study such as freezer, centrifuge, etc. were solely used for the research purpose, the equipment was newly purchased for this study and was handed over to respective hospital so that the equipment can be used for future research in the hospital.

8. Finally, please provide the title (eg, Dr/Prof, Mr/Mrs/Ms/Mx), name, and email address of an author who can be contacted about this statement. This can be the corresponding author.

Name: Dr Ram Hari Chapagain (the co-first author/Coordinating PI in Nepal)
Email: Chapagainrh2007@gmail.com
